# Advances in genomic hepatocellular carcinoma research

**DOI:** 10.1093/gigascience/giy135

**Published:** 2018-12-06

**Authors:** Weitai Huang, Anders Jacobsen Skanderup, Caroline G Lee

**Affiliations:** 1 Computational and Systems Biology, Agency for Science Technology and Research, Genome Institute of Singapore, 60 Biopolis Street, Singapore 138672, Singapore; 2 Graduate School of Integrative Sciences and Engineering, National University of Singapore, 5 Lower Kent Ridge Road, Singapore 117456, Singapore; 3 Department of Biochemistry, Yong Loo Lin School of Medicine, National University of Singapore, Singapore 119077, Singapore; 4 Division of Medical Sciences, Humphrey Oei Institute of Cancer Research, National Cancer Center Singapore, Singapore 169610, Singapore; 5 Duke-NUS Graduate Medical School Singapore, Singapore 169547, Singapore

**Keywords:** hepatocellular carcinoma, next-generation sequencing, somatic mutations, viral integration

## Abstract

**Background:**

Hepatocellular carcinoma (HCC) is the cancer with the second highest mortality in the world due to its late presentation and limited treatment options. As such, there is an urgent need to identify novel biomarkers for early diagnosis and to develop novel therapies. The availability of next-generation sequencing (NGS) data from tumors of liver cancer patients has provided us with invaluable resources to better understand HCC through the integration of data from different sources to facilitate the identification of promising biomarkers or therapeutic targets.

**Findings:**

Here, we review key insights gleaned from more than 20 NGS studies of HCC tumor samples, comprising approximately 582 whole genomes and 1,211 whole exomes mainly from the East Asian population. Through consolidation of reported somatic mutations from multiple studies, we identified genes with different types of somatic mutations, including single nucleotide variations, insertion/deletions, structural variations, and copy number alterations as well as genes with multiple frequent viral integration. Pathway analysis showed that this curated list of somatic mutations is critically involved in cancer-related pathways, viral carcinogenesis, and signaling pathways. Lastly, we addressed the future directions of HCC research as more NGS datasets become available.

**Conclusions:**

Our review is a comprehensive resource for the current NGS research in HCC, consolidating published articles, potential gene candidates, and their related biological pathways.

## Introduction

Based on GLOBOCAN 2012, liver cancer is the second most common cause of death from cancer worldwide. Liver cancer is the fifth most common cancer in males (554,000 cases) and the ninth most common cancer in females (228,000 cases) [1]. The incidence rate is higher in males than females at a male-to-female ratio of 2.4 worldwide, and the mortality-to-incidence rate is as high as 0.94 and 0.98 for males and females, respectively. Hepatocellular carcinoma (HCC) is the most dominant form of primary liver cancer. Geographically, there is a high incidence rate in Africa (northern and western) and Asia (eastern and southeastern), particularly in China, which accounts for 50 percent of all HCC cases [2].

HCC is commonly associated with risk factors such as hepatitis B (HBV) and hepatitis C (HCV) infection, alcohol, mycotoxin Aflatoxin, obesity, and non-alcoholic fatty liver disease; the risk varies depending on gender, geographic region, and ethnicity [2–4]. Early evidence shows the association of HBV and HCV infection with the development of liver cirrhosis and HCC [5, 6]. The HBV vaccine has been available since the early 1980s; and implementation of HBV vaccination programs in 177 of 193 World Health Organization member states are successful in decreasing HCC incidence rates in children [7, 8].

While environmental factors play a role in HCC, multiple recurrent genetic aberrations and the disruption of the host genome due to HBV DNA integration in HBV-associated HCC are reported to cause the dysregulation of genes important for the hallmarks of cancer. Initial studies identified HBV integration sites via HBV DNA probes or polymerase chain reaction assay followed by Sanger sequencing [9–13]. Subsequently, somatic alterations such as mutations, gene copy number changes, and chromosomal rearrangements detected in the HCC-derived cell lines were found to affect the expression of oncogenes and tumor suppressor genes [14, 15]. Progress in the mapping of each viral integration site and genetic aberration in HCC patients was ad hoc and slow before the advent of next-generation sequencing (NGS).

NGS technologies, including RNA-sequencing (RNA-seq), whole-exome sequencing (WXS), and whole-genome sequencing (WGS), form the foundation of today's discovery-based genomics research. With the reduced cost of massively parallel sequencing technologies over the last decade [16], there has been an increasing number of genomic liver cancer studies providing new insights about liver cancer. Pioneering NGS studies conducted on patient samples have shown a tremendous increase in our understanding of HBV viral integration patterns [17–19] as well as somatic alterations found in liver cancer [20–22]. The large amount of sequencing data generated has been archived on data servers worldwide, enabling researchers to perform integrative analyses that will lead to new findings. However, maneuvering through literature and data repositories to locate and access this information remains a tedious process.

**Figure 1:**
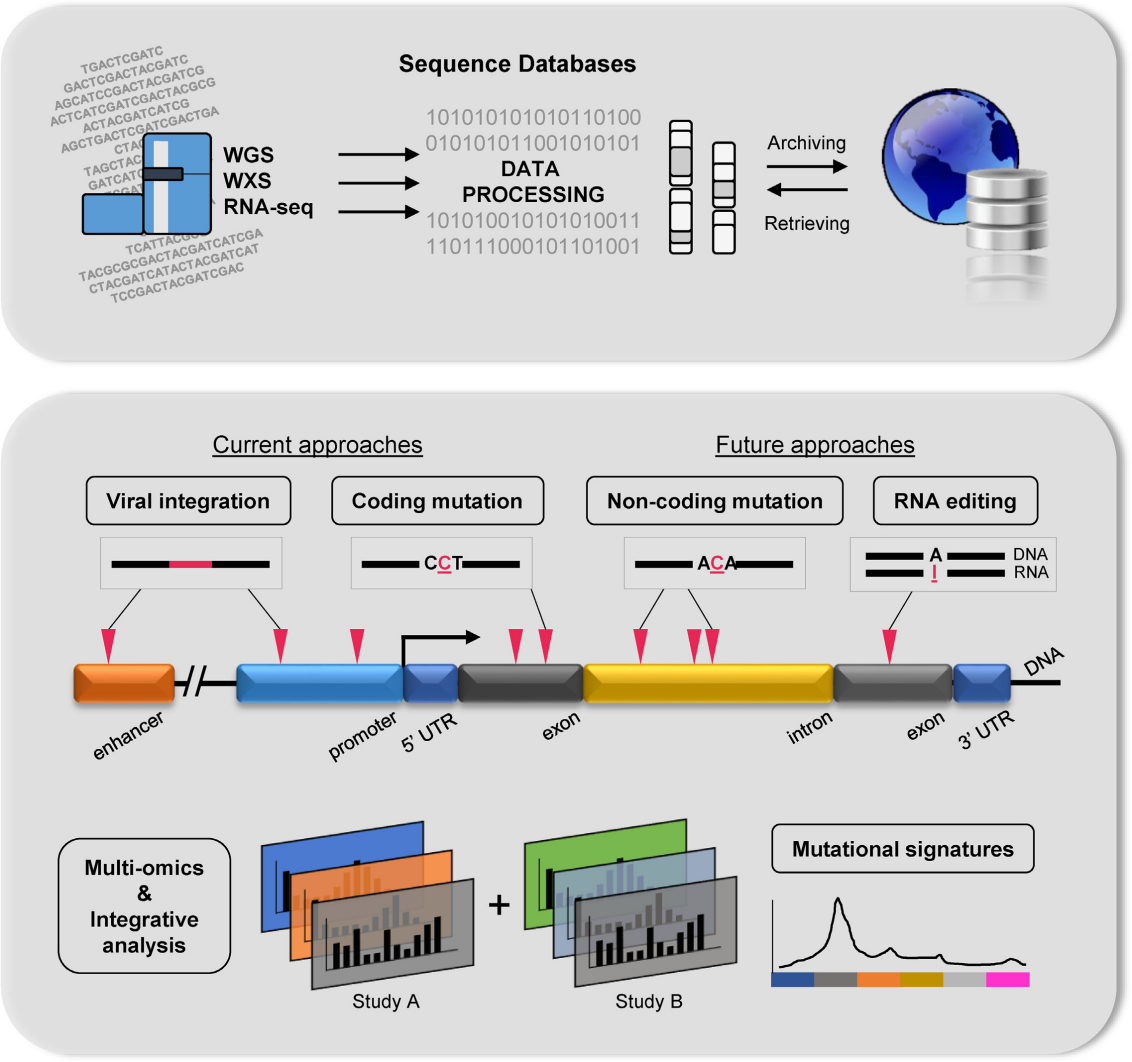
Summary of NGS databases in liver cancer showing its current and potential research direction.

Here, we introduce and consolidate all existing NGS-based studies on liver cancer (Fig. 1). Only the most relevant studies, conducted using NGS in HCC, have been listed in a recent review [23]. Our NGS-based resource is a complete list of data samples of approximately 582 whole genomes and 1,211 whole exomes. It summarizes the key research and clinical findings from each article with direct links to all publicly available WGS/WXS liver cancer datasets to promote better knowledge and data facilitation. The key findings of somatic mutations, HBV integrations, and mutational signatures reported from recent high-throughput studies and related integrative studies are discussed. We highlight key genes reported across multiple studies found to have recurrence of somatic mutations or HBV integration events. Additionally, we provide a meta-analysis of the pathways that these alterations dysregulate. Finally, we discuss future directions and trends in liver cancer research via the analysis of high-throughput data.

## NGS Resources

Raw sequencing data, read alignment, and annotations from NGS platforms can be accessed via National Center for Biotechnology Information (NCBI)-Sequence Read Archive (SRA) [24], European Molecular Biology Laboratory - European Bioinformatics Institute (EMBL-EBI), European Nucleotide Archive [25], and DNA Data Bank of Japan-SRA [26]. The National Cancer Institute's Genomic Data Commons [27] currently hosts genomic data from the Cancer Genome Atlas (TCGA) project that consists of multiple cancer types. There are currently 377 liver hepatocellular carcinoma samples with data from WXS, single nucleotide polymorphism (SNP)-array, methylation, mRNA, and microRNA profiling. Gigadb [28] is a repository for open-access data associated with the *GigaScience* journal [29], which currently holds an HCC dataset from 88 individuals [30]. The International Cancer Genome Consortium (ICGC) [31] is a global effort to coordinate large-scale cancer genome studies by providing a comprehensive catalogue of somatic mutations across 50 cancer types, which generates approximately 500 samples each [32]. While primary data files are stored on NCBI and/or EBI, ICGC provides interpreted datasets for somatic mutation calls and incorporates transcriptomic and DNA methylation analyses from the same tumor samples.

We reviewed and consolidated a comprehensive list of liver cancer studies that have analyzed high-throughput genomics data (Table 1). The majority of the studies have their raw and/or processed data available on the above-mentioned public databases (Table 1, Data URL). These studies are mainly focused on liver cancer patients from a single country of the East Asian population (Table 1, Population). Genomics data from the Japanese population constitutes the largest sample size [21, 33–37], including a collection of 300 whole genomes reported in a recent study [33]. NGS studies were also performed with HCC patients from China [38–40], Hong Kong [18, 39, 41, 42], Korea [43–45], Taiwan [46, 47], Singapore [19], and Europe [48–51]. Several studies have a collection of samples from various ethnicities (TCGA) or multiple sources [17, 22, 52–54].

**Table 1:** Summary of NGS resources and their key findings from liver cancer studies

No.	Reference	Data URL	Sample type/total cases	Population	Viral status	Key findings
1	TCGA	https://dcc.icgc.org/projects/LIHC-US	54 WGS (52 HCC, 1 ICC, 1 FC)	39 White, 9 Asian, 3 African American, 3 Unknown	7 HCV, 7 HBV, 40 NBNC	TCGA-LIHC-WGS
2	TCGA	https://portal.gdc.cancer.gov/projects/TCGA-LIHC	376 WXS (366 HCC, 7 cHCC/ICC, 3 FC) + 371 RNA-seq (361 HCC, 7 cHCC/ICC, 3 FC)	187/184 White, 160/158 Asian, 17 African American, 2 American Indian/Native, 10 Unknown	49 HCV, 102 HBV, 8 HBV/HCV, 217 NBNC	TCGA-LIHC-WXS
3	Letouze et al. (2017) Nature Comm. https://doi.org/10.1038/s41467-017-01358-x	https://www.ebi.ac.uk/ega/studies/EGAS00001002408	44 WGS (35 HCC, 5 HCA, 4 FC)	40 European, 4 African	4 HCV, 5 HBV, 35 NBNC	1. Analysis of more than 300 genomes highlighted 10 mutational signatures, including ubiquitous as well as sporadic signatures.2. Reconstruction of the temporal evolution in driver mutations and signatures revealed the clonal architecture in each tumor.
4	Ng et al. (2017) Sci. Transl. Med. https://doi.org/10.1126/scitranslmed.aan6446	https://www.ebi.ac.uk/ega/studies/EGAS00001002301	98 WXS (HCC)	Asian (Taiwan)	21 HCV, 56 HBV, 3 HBV/HCV, 10 NBNC, 8 N.D.	1. Distinct mutational signatures were identified in the whole exomes of HCC patients with aristolochic acid exposure.2. The aristolochic acid signature also revealed known cancer driver genes, TP53 and CTNNB1, mutated 54 and 24 percent of the total HCC cases respectively.
5	Zhang et al., (2017) Gastroenterology. https://doi.org/10.1053/j.gastro.2017.03.024	Unknown	49 WGS + 13 WXS (HCC)	Asian (China)	38 HBV, 9 NB, 2 N.D.	1. Aflatoxin-associated HCCs were reported to frequently contain C>A transversions, sequence motif GCN, and strand bias.2. Frequent mutations identified in the adhesion G protein-coupled receptor B1 gene (ADGRB1) were found to be associated with increased capillary density of the tumor tissue.
6	Fujimoto et al. (2016) Nature Genetics. https://doi.org/10.1038/ng.3547	https://dcc.icgc.org/projects/LIRI-JP https://www.ebi.ac.uk/ega/studies/EGAS00001000671	300 WGS (268 HCC, 24 ICC, 8 cHCC/ICC) + 254 RNA-seq	Asian (Japan)	159 HCV, 82 HBV, 4 HBV/HCV, 55 NBNC	1. Coding and noncoding regions (including NEAT1 and MALAT1) were identified to have significant mutations.2. Structural variation analysis revealed cancer-related genes (e.g., TERT and NCOR1) that led to altered expression.
7	Hirotsu et al. (2016) Hepatology Research https://doi.org/10.1111/hepr.12663	http://trace.ddbj.nig.ac.jp/DRASearch/submission?acc=DRA003210	9 WXS (HCC)	Asian (Japan)	1 HBV, 5 HCV, 3 NBNC	1. Targeted deep sequencing analysis showed that TP53 (3/9 cases) and CTNNB1 (2/9 cases) were recurrent missense mutations in HCCs.2. Functional analysis of the β-catenin H36P mutant was observed to be resistant to protein degradation and to promote HCC cell proliferation.
8	Fujimoto et al. (2015) Nature Comm. https://doi.org/10.1038/ncomms7120	https://dcc.icgc.org/projects/LIRI-JP	90 WGS (60 HCC, 7 cHCC/ICC, 22 ICC, 1 CoCC) + 69 RNA-seq	Asian (Japan)	60 HCC: 23 HBV, 29 HCV, 3 HBV/HCV, 5 NBNC	1. cHCC/ICC and CoCC showing biliary epithelial differentiation (LCB) have recurrent mutations in the TERT promoter and chromatin regulators.2. Hepatitis-positive HCC and cHCC/CC had a larger frequency of TERT promoter mutations and a lower frequency of KRAS and IDH1/2 mutations than hepatitis-negative LCB.
9	Kang et al. (2015) Genomics https://doi.org/10.1016/j.ygeno.2014.11.005	http://www.ebi.ac.uk/ena/data/view/ERP001196 http://gigadb.org/dataset/100034	9 WGS + RNA-seq (HCC)	Asian (Hong Kong)	HBV	1. An improved bioinformatics pipeline detects RNA-editing events in HCC tumor and matched adjacent tissues.2. Varying editing degrees were significant in 13 cancer-related genes from 18 editing sites and one gene with editing in the CDS region between normal and tumor tissues.
10	Schulze et al. (2015) Nature Geneticshttps://doi.org/10.1038/ng.3252	https://dcc.icgc.org/projects/LICA-FR https://www.ebi.ac.uk/ega/studies/EGAS00001000217	236 WXS (HCC)	European (193 France, 9 Spain, 41 Italy)	57 HCV, 29 HBV, 4 HBV/HCV, 142 NBNC	1. Mutational signatures were significantly associated with demographic, etiological, molecular features.2. Signature 23 that contained predominantly C>T mutations is consistent with the study by Totoki et al. (2011).
11	Nault et al. (2015) Nature Geneticshttps://doi.org/10.1038/ng.3389	https://www.ebi.ac.uk/ega/studies/EGAS00001000217	193 WXS (HCC)	European (France)	36 HCV, 22 HBV, 135 NBNC	1. Clonal integration of the adeno-associated virus type 2 (AAV2) were identified in 11of 193 HCCs.2. AAV2 integrations occurred in known cancer driver genes including TERT, CCNA2, CCNE1, KMT2B, and TNFSF10.
12	Dong et al. (2015) PLoS Onehttps://doi.org/10.1371/journal.pone.0123175	http://www.ncbi.nlm.nih.gov/bioproject/279878	55 RNA-seq (HCC)	Asian (China)	49 HBV, 7 NBNC	1. MLL4 was identified as the most frequent HBV integration site (8/44 cases).2. Gene expression levels of the 8 MLL4-integration-positive samples were significantly higher than wild-type tumor and adjacent tissues.
13	Totoki et al. (2014) Nature Geneticshttps://doi.org/10.1038/ng.3126	http://www.ncbi.nlm.nih.gov/gap/?term=phs000509 https://www.ebi.ac.uk/ega/studies/EGAS00001000389	503 WXS (488 HCC, 2 cHCC/ICC, 13 ICC)	414 Asian (Japan), 50 Caucasian, 14 US-Asian, 11 African American, 14 N.D.	212 HCV, 117 HBV, 12 HBV/HCV, 150 NBNC, 9 N.D.	1. Thirty candidate driver genes, including non-recurring mutated genes BRD7, MEN1, TSC2, SCRAP, and NCOR1 were identified.2. Distinct substitution signatures were detected between the various ancestries and gender but not associated with viral status.
14	Shirashi et al. (2014) PLoS Onehttps://doi.org/10.1371/journal.pone.0114263	https://www.ebi.ac.uk/ega/datasets/EGAD00001001035	22 WGS + RNA-seq (HCC)	Asian (Japan)	HBV	1. Comparison of genomic and transcriptomic reads identified 292 genomic mutation-related splicing aberrations.2. Twenty-three of 33 HBV-human fusions were reported to affect TERT, FN1, MLL4, as well as concentrated around the HBx genes.
15	Fernandez-Banet et al. (2014) Genomics https://doi.org/10.1016/j.ygeno.2014.01.003	http://www.ebi.ac.uk/ena/data/view/ERP001196 http://gigadb.org/dataset/100034	88 WGS (HCC)	Asian (Hong Kong)	81 HBV, 7 NBNC	1. A total of 4,314 somatic genomic rearrangement (GR) events were detected and annotated at the single-nucleotide resolution.2. Five HCC tumors harbored chromothripsis on chromosomal arms 1q, 8q, and 5p; 13 genes, including CEBPB, MCL1, and AXIN1, were significantly affected by GR.
16	Jhunjhunwala et al. (2014) Genome Biology https://doi.org/10.1186/s13059-014-0436-9	https://www.ebi.ac.uk/ega/studies/EGAS00001000824	12 WGS + RNA-seq (HCC)	Samples obtained from commercial sources	11 HBV, 1 NBNC	1. Recurrent mutations in TP53, AXIN1, and CTNNB1 were detected as well as a rare find in LAMA2 (6/42 cases) and IDH1 (2/42 cases).2. The activation of TERT was either due to viral integrations in its promoter or its translocation to another chromosomal region.
17	Ahn et al. (2014) Hepatology https://doi.org/10.1002/hep.27198	Unknown	231 WXS (HCC)	Asian (Korea)	167 HBV, 22 HCV, 42 NBNC	1. Nine significantly mutated genes and cellular pathways such as p53, Wnt, PIK3/Ras, cell cycle, and chromatin remodeling account for ∼80% of the mutations identified in the 231 tumors.2. Genetic aberrations in the cell cycle pathway genes (RB1, MYC, CCND1, RBL2) were associated with cancer-specific and recurrence-free survival.
18	Woo et al. (2014) PLoS One https://doi.org/10.1371/journal.pone.0115152	Unknown	12 WXS (HCC)	Asian (Korea)	HBV	1. Tumor-specific genes such as CTNNB1, TTN, SETD2, and ALK have been identified.2. The T>A transversions were present significantly and exclusively in tumor-specific variants.
19	Ouyang et al. (2014) BMC Medical Genomics https://doi.org/10.1186/1755-8794-7-2	https://trace.ddbj.nig.ac.jp/DRASearch/submission?acc=SRA076160	4 WGS (HCC)	Asian (Korea)	HBV	1. Analysis of the mutational spectrum showed that C>T transition rates within the coding regions were the highest.2. Altered pathways in primary tumor were Wnt, JAK-STAT, cell cycle, and focal adhesion pathways, while tight junction, focal adhesion, and ErbB/MAPK pathways were affected in the metastases.
20	Kan et al. (2013) Genome Research https://doi.org/10.1101/gr.154492.113	http://www.ebi.ac.uk/ena/data/view/ERP001196 http://gigadb.org/dataset/100034	88 WGS (HCC)	Asian (Hong Kong)	81 HBV, 7 NBNC	1. The study reveals recurrent mutations in TP53, CTNNB1 and AXIN1, two genes (JAK1, LRPB1) commonly mutated in other cancers as well as six genes previously not reported.2. Pathways affected include Wnt, cytokine-induced JAK/STAT, G1/S cell cycle, and apoptosis.
21	Toh et al. (2013) Carcinogenesis https://doi.org/10.1093/carcin/bgs406	Unknown	48 FLX-Seq (HCC)	Asian (Singapore)	48 HBV	1. Preferential integration of HBV into the TERT promoter (6/97 cases).2. The 3ʹ-end of the HBV X protein is the preferred HBV genomic region detected in the integration events.
22	Cleary et al. (2013) Hepatology https://doi.org/10.1002/hep.26540	http://www.ncbi.nlm.nih.gov/projects/gap/cgi-bin/study.cgi?study_id=phs000627.v1.p1	87 WXS (HCC)	Samples obtained from Canada, North Carolina, and CHTN	19 HCV, 38 HBV, 30 NBNC	1. Thirteen significantly mutated genes identified include CTNNB1, TP53, CPA2, IGSF3, and KEAP1 as well as four significantly mutated gene families.2. Further validation of the MLL gene family revealed MLL4 (6/13 missense mutations) to be a potential driver gene of HCC.
23	Lin et al. (2013) Oncogene https://doi.org/10.1038/onc.2013.424	https://trace.ddbj.nig.ac.jp/DRASearch/study?acc=SRP007560	55 RNA-seq (HCC)	Asian (Taiwan)	20 HBV, 18 HCV, 17 NBNC	1. Putative mRNA sequences filtered via Cufflinks de novo assembly identified, DUNQU1, a 101-amino-acid peptide encoded by 3 exons.2. Analysis of alternative splicing in transcripts revealed three cancer-related events in FGFR2, EXOC7, and ADAM15.
24	Fujimoto et al. (2012) Nature Genetics https://doi.org/10.1038/ng.2291	https://dcc.icgc.org/projects/LINC-JP	27 WGS (HCC)	Asian (Japan)	11 HBV, 14 HCV, 2 NBNC	1. TP53 and CTNNB1, as well as ATM, ARID1A, ERRF11, WWP1 mutations were detected in the tumors.2. Gene-set enrichment analysis identified several genes associated with chromatin regulation.
25	Sung et al. (2012) Nature Genetics https://doi.org/10.1038/ng.2295	http://www.ebi.ac.uk/ena/data/view/ERP001196 http://gigadb.org/dataset/100034	88 WGS (HCC)	Asian (Hong Kong)	81 HBV, 7 NBNC	1. A total of 179 of the 399 HBV integration breakpoints were identified in known coding genes.2. HBV integrations led to increased gene expression of TERT, MLL4, and CCNE1.
26	Guichard et al. (2012) Nature Genetics https://doi.org/10.1038/ng.2256	https://www.ebi.ac.uk/ega/studies/EGAS00001000217	24 WXS (HCC)	European (France)	4 HCV, 1 HBV, 19 NBNC	1. A total of 850 mutations corresponded to single-nucleotide variants, particularly C>T changes that occur more frequently in non-cirrhotic liver HCC tumors.2. Major pathways with frequently altered genes identified include Wnt and p53 pathways as well as four recurrent mutations (ARID1A, RPS6KA3, NFE2L2, and IRF2) previously not reported.
27	Jiang et al. (2012) Genome Research https://doi.org/10.1101/gr.133926.111	http://www.ncbi.nlm.nih.gov/projects/gap/cgi-bin/study.cgi?study_id=phs000384.v1.p1	4 WGS + RNA-seq (HCC)	Samples obtained from commercial sources	3 HBV, 1 NBNC	1. RNA-seq expression analysis revealed the impact of HBV integrations on adjacent transcription activation of MLL4 and ANGPT1 in different patients.2. There is a strong bias of viral-fusion transcripts containing HBV genome sequences near its direct repeat 1 (DR1) region.
28	Huang et al. (2012) Nature Genetics https://doi.org/10.1038/ng.2391	http://www.ncbi.nlm.nih.gov/bioproject/PRJNA167270	10 WXS (HCC)	Asian (China)	8 HBV, 2 NBNC	1. The comparison between matched samples of HBV-associated HCC individuals (primary tumor vs. portal vein tumor thromboses) reveals 65 mutations including TP53 and ARID1A.2. ARID1A mutations were also identified in four HCC cell lines with high metastatic potential.
29	Totoki et al. (2011) Nature Genetics https://doi.org/10.1038/ng.804	https://dcc.icgc.org/projects/LINC-JP	1 WGS (HCC)	Asian (Japan)	HCV	1. The study identified somatic substitutions patterns predominantly from T>C and C>T transitions.2. Somatic alterations include well-known tumor suppressors TP53 and AXIN1 as well as five other genes found commonly mutated in other cancers.
30	Li et al. (2011) Nature Genetics https://doi.org/10.1038/ng.903	Unknown	139 WXS (HCC)	US (44 White, 15 Black, 9 Asian, 1 Hispanic, 1 Arabic, 8 Unknown), China (61 Asian)	43 HCV, 50 HBV, 2 HBV/HCV, 44 NBNC	1. Somatic mutations were found in five genes (CTNNB1, TP53, ARID2, DMXL1, and NLRP1).2. Six of nine of the samples containing ARID2 mutations also contained CTNNB1 mutations but none of them contained TP53 mutations.
			Total		Total	
			582 WGS; 1211 WXS; 778 RNA-seq; 48 FLX-seq		43.71% HBV; 21.13% HCV; 34.48% NBNC	

Multiple findings have already been reported on the patient samples from Japan [33–35], Hong Kong [18, 41, 42], and Europe [48–51], as well as integrative studies from multiple sources [54, 55] or commercial sources [17, 53]. Here, we review approximately 582 whole genomes, 1,211 exome, and 778 RNA-seq samples of liver cancer patients (Table 1, Total cases). Of patients with known viral status, 44 percent are infected with HBV, 21 percent with HCV, while 35 percent are not infected by either HBV or HCV (NBNC) (Table 1, Viral status). Several of the groups have also employed NGS to examine HBV integrations in HCC patients [17–19, 56].

## Key Findings

### Somatic genomic alterations

By comparing matched normal and tumor samples, computational algorithms have identified a number of likely cancer-causing point mutations and insertions/deletions (indels). Somatic alterations such as point mutations, indels, structural variants, and copy number alterations have been identified in 1 or more of the 85 genes that we have included in Table 2. Recurrent mutations in 12 genes (*TP53, CTNNB1, AXIN1, ALB, ARID2, ARID1A, RPS6KA3, APOB, RB1, CDKN2A, LRP1B*, and *PTEN*) were reported in multiple studies. In this section, we discuss five genes (*ALB, ARID2, RB1, BRD7*, and RPL22) that were reported to show all four types of somatic alterations. To gain further insights into the genes with reported somatic mutations, their gene expression (tumor/normal fold-change) and clinic-pathological clinical information (histologic grade and survival) from the TCGA HCC cohort are also presented.

**Table 2:** Summary of mutations in liver cancer identified through high-throughput genomics data including their association with gene expression and clinical phenotype

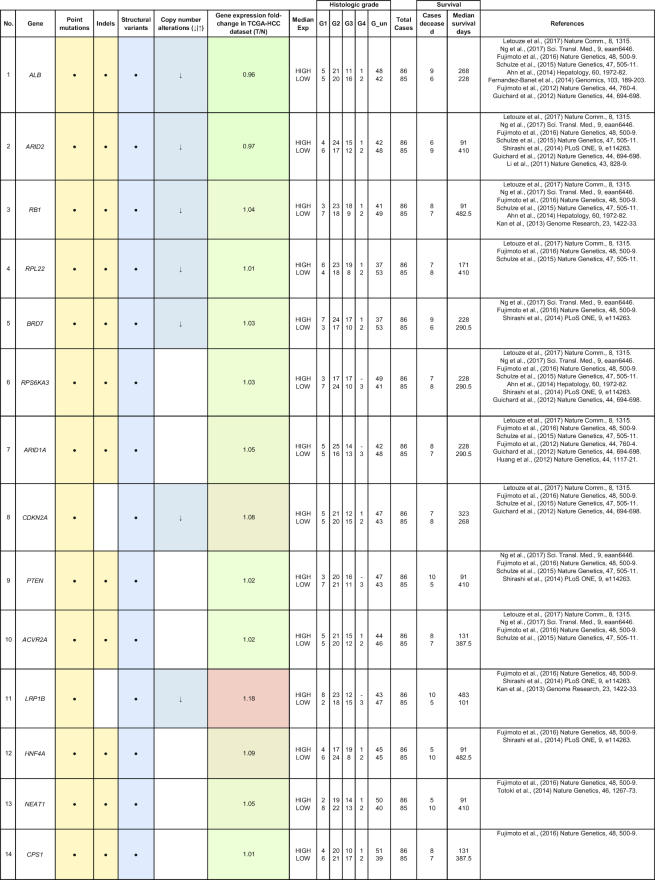
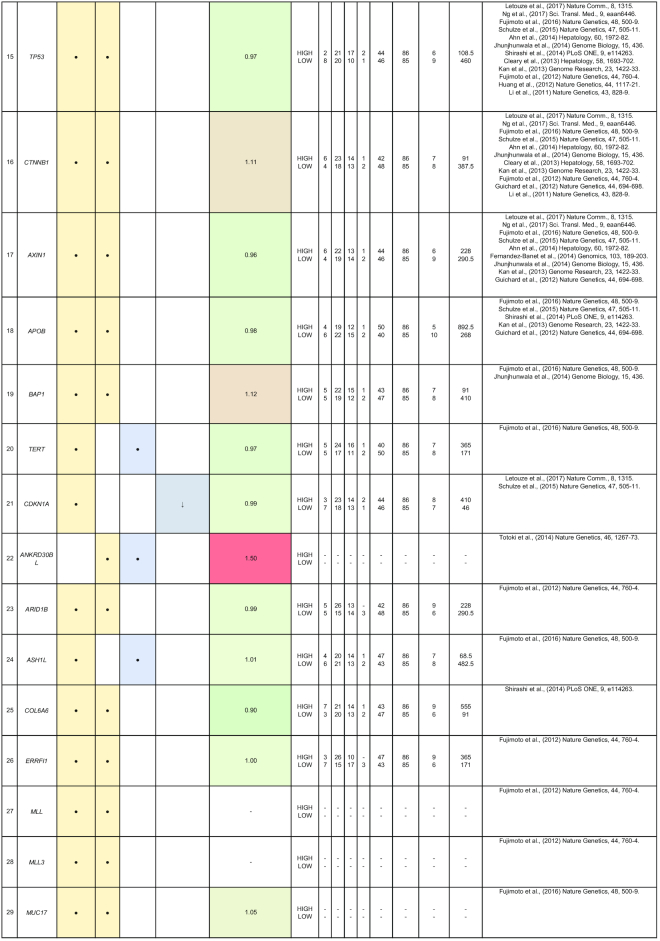
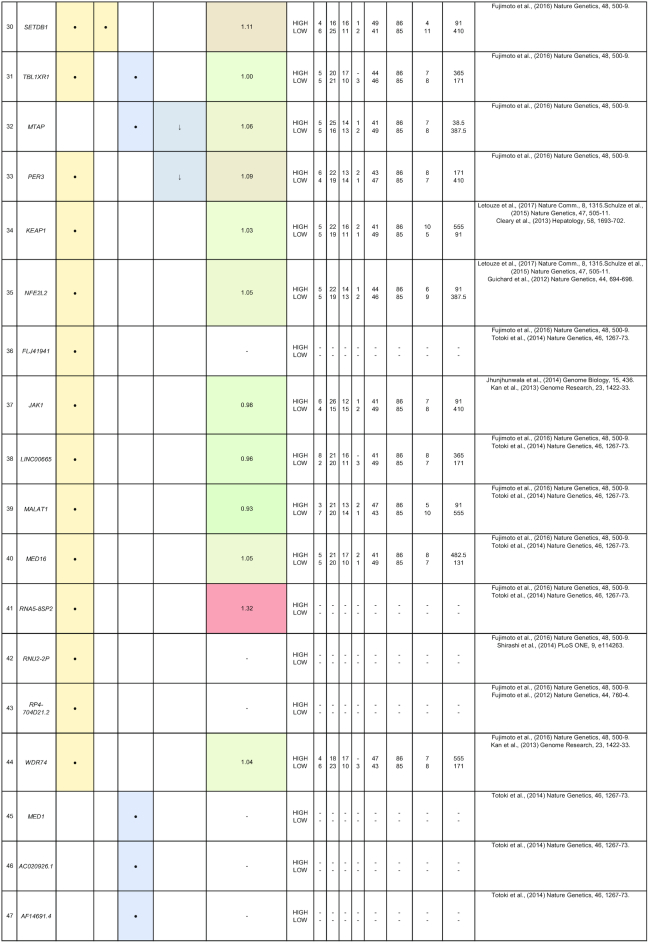
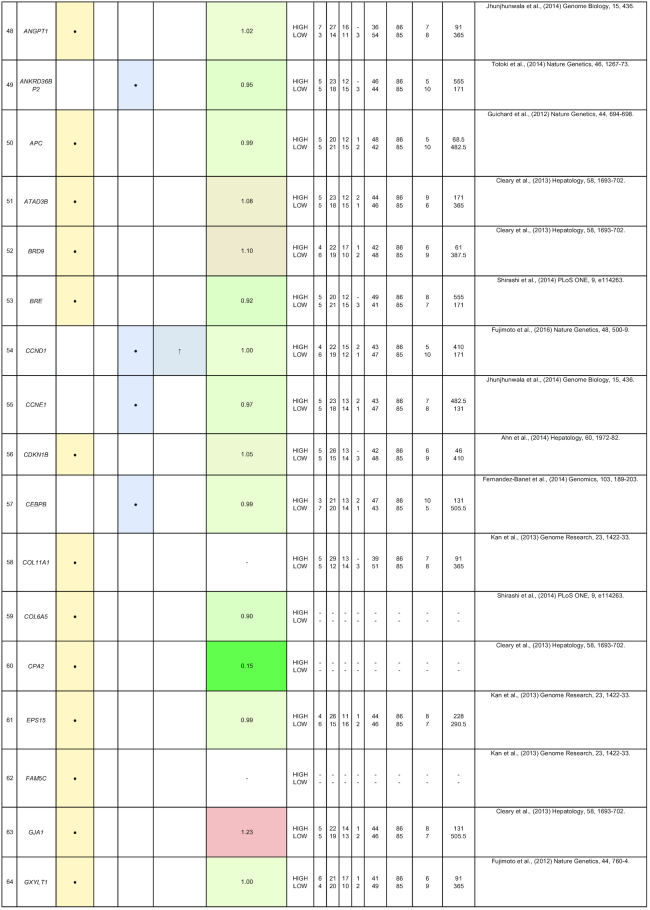
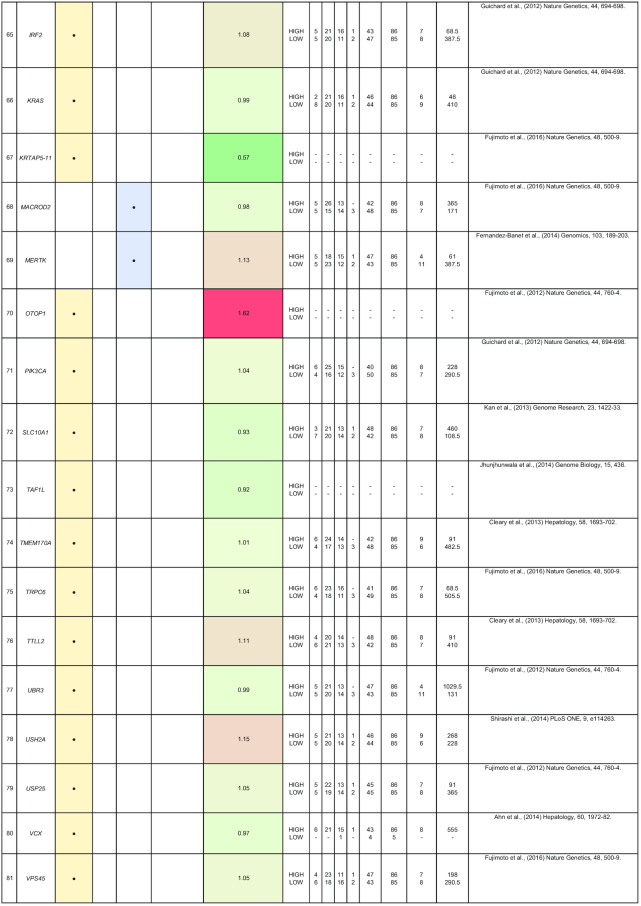
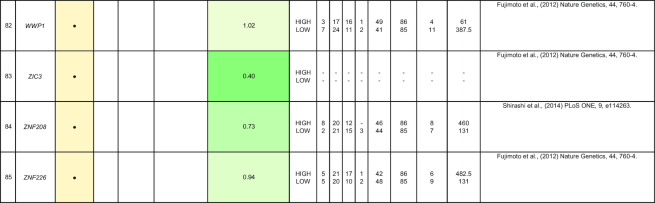

The table indicates the nature of the mutation (single-nucleotide variant [SNV], indels, structural variants, or copy number alterations) in the coding regions. The fold-change of the gene is obtained from the TCGA microarray analysis on HCC patient samples. Histologic grade refers to degree of tumor grade: G1 to G4; G_un indicate cases with unidentified histologic grading. The cases are segregated into HIGH or LOW based on their median gene expression (Median Exp). SNVs and indel mutations are indicated by the yellow box (●), structural variants by the blue box (●), and copy number alterations by the gray box (↓|↑).

*ARID2* belongs to the SWI/SNF-related chromatin remodeling complexes and is identified as a tumor suppressor that is frequently mutated in HCC patients [22, 42, 48]. In addition, gene expression profiling of *ARID2*-deficient HCC cell lines reveals negative regulation of UV-response gene sets, suggesting that *ARID2* may be involved in DNA repair processes [57]. *ARID2* is also involved in HCC via the effects of hepatitis B and C infection. In HBV-related HCC, the HBV X protein is reported to suppress *ARID2* expression, leading to increased hepatoma tumorigenesis [58]. *ARID2* mutations are also significantly associated (*P* = 0.046) with HCV-related HCC [22]. These findings suggest that *ARID2* is a critical tumor suppressor in hepatitis virus-related HCC progression.

Similar to *ARID2, BRD7* is also a component of the SWI/SNF remodeling machinery and a putative tumor suppressor reported with significant truncating mutations in HCC [55]. Loss-of-function mutations at the *BRD7* gene locus are frequently observed (7/268) in HBV-associated HCC patients [33]. *BRD7* expression is also reported to be associated with the clinical characteristics in HCC (tumor size, tumor stage, and survival) [59]. HCV infections repress *BRD7* expression *in vitro*, resulting in the dysregulation of hepatoma cell proliferation [60]. *BRD7* also negatively regulates PI3K signaling by binding to the inter-SH2 (iSH2) domain of p85, leading to the impairment of p88/p110 complex formation [61].

The *ALB* gene encodes for the most abundant plasma protein, albumin, synthesized exclusively by hepatocytes [41]. Blood albumin tests that deviate from the normal healthy range often indicate dysregulation of protein production in the liver and other liver-associated issues. Somatic mutations at the *ALB* gene locus were reported in multiple studies, including genomic rearrangements in 10% (9/88) of Chinese HCC patients [41] as well as point mutations clusters and indels in Japanese HCC patients [33]. *ALB* is touted as a liver cancer driver gene as it is significantly enriched with damaging mutations in the European population [50]. Highly expressed genes such as *ALB* and *APOB* have been shown to be strongly enriched with indels, which are characteristic of replication slippage errors resulting from conflicts between the replication and transcription machineries [51]. Hence, low albumin levels may contribute to liver cancer progression.

*RB1* is a key inhibitor of cell cycle progression that harbors multiple nonsense mutations and genomic deletions in HCC patients [33, 42, 43, 50]. *RB1* is found to be predominantly mutated in Asian Americans (10/53 patients) as compared to European Americans (2/101 patients) [62]. The inactivation of the RB pathway in Rb family triple knockout mice resulted in the development of HCC [63]. A study reveals that in 16/40 HCC patients, DNA methylation abnormalities were observed in CpG island 85 (CpG85) located within intron 2 of the *RB1* gene, which can potentially regulate the expression of the *RB1-E2B* alternative transcript [64]. In addition, *RB1* mutations are also significantly associated with reduced cancer-specific and recurrence-free survival after resection in HCC patients [43, 50]. It is thus worthwhile to further characterize *RB1* mutations, as they are reported to have a significantly higher mutation rate in HBV-related HCCs [42, 43].

*RPL22*, another gene that is reported to exhibit all 4 different types of mutations (single-nucleotide variant, indels, structural and copy number variation), encodes for a ribosomal 60S subunit protein. It was reported to be significantly mutated in Japanese (5/268 patients) and European (7/242) HCC patients [33, 50]. *RPL22* was identified through pan-genomic characterization as a driver gene with significant somatic alterations in adenocortical carcinoma [65]. A study of microsatellite instability-positive gastric cancers also identified *RPL22* as a recurrently mutated gene with single base deletions [66]. Therefore, there is potential for more research to be conducted to fully determine the functional roles of *RPL22* in HCC.

### HBV integration

The HBV genome often integrates into the chromosomes of liver cells, resulting in alterations of the host genome. Recent findings have confirmed that the viral transcription/replication initiation site, DR1 (located near the 3′ end of the *HBx* gene and the beginning of the Precore/Core gene), is the preferred region to be integrated into the host chromosome [11, 17, 19]. More HBV integration events were identified in tumor as compared to their matched normal samples [18]. In HCC tumors, studies show that HBV integration was randomly distributed throughout the human genome [17, 18, 33]. In a group of 48 HCC patients from the Singapore cohort, HBV integrations were significantly enriched in the q arm of chromosome 10 and correlated with poorly differentiated tumors [19].

From the NGS studies, we have consolidated a comprehensive table of viral integration events that occurred in HCC patients (Table 3). There are multiple integration events in the promoter, 3′UTR, coding sequence and/or intronic region of the *CCNE1* [67]*, TERT* [19, 35, 37]*, CDK15* [37]*, ROCK1 [**18*], *FN1* [*68*], *APOA2* [67], and *MLL4* [17, 18, 67] genes. HBV was reported in several studies to integrate into the *CCNE1* and *TERT* genes [18, 33, 53]. *CDK15, ROCK1, FN1, APOA2*, and *MLL4* are less frequently reported to be sites of integration for HBV.

**Table 3:** Summary of HBV viral integration events occurring in HCC patients identified through high-throughput genomics data

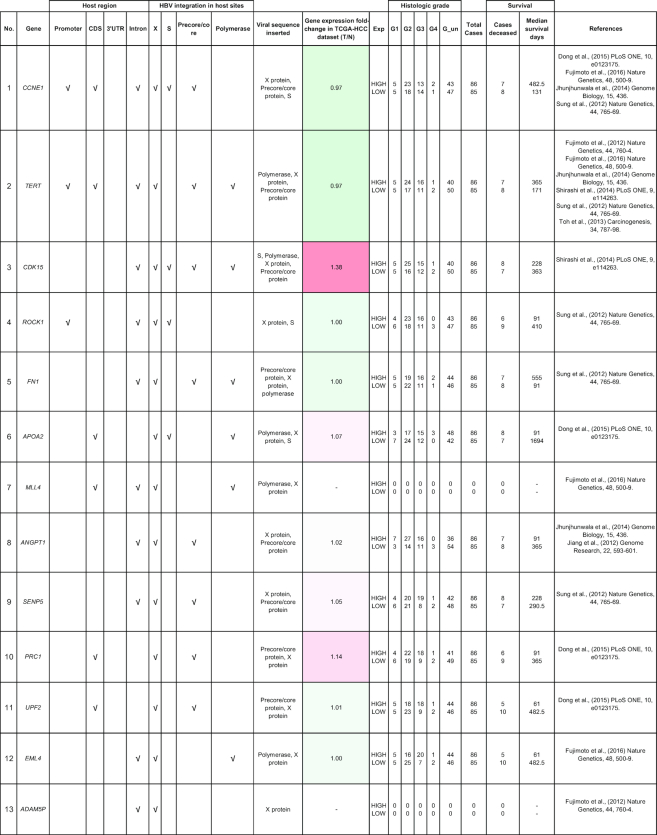
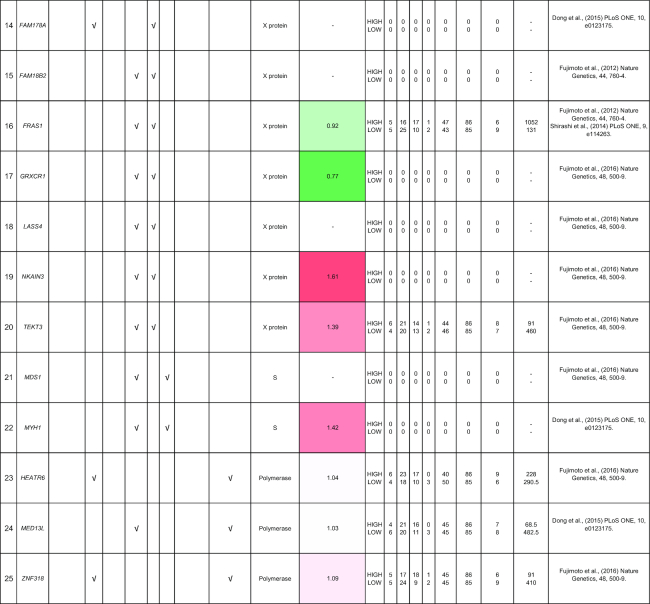

The table indicates the genes and where the integration events occur. The fold-change of the gene is obtained from the TCGA microarray analysis on HCC patient samples. Histologic grade refers to degree of tumor grade: G1 to G4; G_un indicates cases with unidentified histologic grading. The cases are segregated into HIGH or LOW based on their median gene expression (Median_Exp).

*CCNE1* encodes for the cyclin E1 protein that is a regulatory subunit of *CDK2* involved in the G1/S phase of the cell cycle. *CCNE1* amplification has been reported to be the mechanism of resistance in *ER*-positive and *HER2*-positive breast cancers as well as high-grade serous ovarian cancer [69–72]. HBV integrations within the *CCNE1* have been reported in 4 of 76 HBV-positive HCC samples and resulted in significantly increased expression of *CCNE1* [18]. The molecular mechanism of *CCNE1* mutations in HCC patients has yet to be fully elucidated.

The previously reported recurrent integration site at the *TERT* promoter was found by several high-throughput genomic studies to be the most frequent site for integration [19, 33, 73, 74]. Disruption of the *TERT* promoter is likely to cause the dysregulation of the telomerase reverse transcriptase (TERT) expression, which plays important roles in cancer development due to its diverse telomere-independent functions in Wnt pathway signaling, cell proliferation, and DNA-damage repair [75]. Viral sequences may act as enhancers where the closer the HBV is integrated to the transcription start site of *TERT*, the higher the mRNA expression of TERT [19].

Chimeric *HBx/MLL4* fusion transcripts containing the *HBx* promoter and Open Reading Frame (ORF)fused to the exon 4 and 5 of *MLL4* were initially detected in 4 of 10 HCC patients [76] and subsequently confirmed in later studies and reported to lead to increased *MLL4* expression [17, 18, 67]. In a Chinese cohort, 8 of 44 patients were found to have *HBx/MLL4* fusion transcripts, resulting in a higher expression of *MLL4* gene [67]. The chimeric transcript lacks the AT-hook DNA-binding domain of *MLL4*, hence, it may act as a dominant negative allele [17].

*CDK15* encodes for the cyclin-dependent kinase 15 and is a serine/threonine protein kinase. In one study, *CDK15* contributed to the effects of tumor necrosis factor-related apoptosis-inducing ligand resistance by possibly regulating the phosphorylation of survivin (Thr34) [77]. Interestingly, multiple HBV-*CDK15* fusion transcripts were detected in an HCC patient, including one in-frame fusion, which caused CDK15 over-expression [37]. However, like many of the other genes where HBV integrations have been identified, the function of *CDK15* in HCC remains unclear. Hence, there is great potential to further investigate HBV integrations in HCC.

It is noteworthy that *CCNE1, TERT, and ANGPT1* not only harbor somatic mutations (Table 2), they are also reported to be sites for viral integrations (Table 3). *CCNE1* has been reported with structural variant alterations and HBV integrations, while *TERT* has been reported with point mutations, structural variant alterations, and HBV integrations, suggesting that deregulation of these genes may play important roles in tumorigenesis. *ANGPT1* (Angiopoietin-1), a ligand for Tie2 vascular endothelial-specific receptor tyrosine kinase, involved in the induction of HCC neovascularization and disease progression [78–80], was reported to harbor point mutations and HBV integrations in its intronic regions. *ANGPT1* and Angiopoietin-2 (*ANGPT2*) were over-expressed in 68 and 81 percent of poorly differentiated HCC tumors, respectively [81]. However, high *ANGPT2* expression, but not *ANGPT1*, showed correlation in the disease-free survival of 60 HCC patients [82]. The role of *ANGPT1* in tumor angiogenesis remains unclear.

### Pathways of somatic mutated genes and mutation signatures

**Figure 2:**
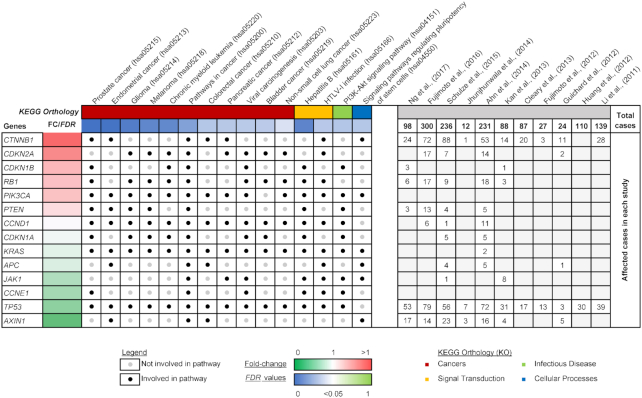
Reported genes with somatic mutations that are significantly involved in KEGG pathways.

Pathway analysis based on the Kyoto Encyclopedia of Genes and Genomes (KEGG) was performed using the Database for Annotation, Visualization and Integrated Discovery (DAVID v6.8) to identify pathways that were altered by somatic mutations in the TCGA HCC cohort [83, 84]. Seventy-nine of the 85 genes in our list of somatic mutations have identifiable DAVID IDs, of which 45 genes can be categorized in KEGG pathways. Fifteen significant pathways were identified (*FDR <0.05*) from the 45 genes, of which 14 genes are found to be involved in more than one of the pathways (Fig. 2). All 14 genes are involved in pathways in cancer, including other significant cancer types: prostate, endometrial, glioma, melanoma, chronic myeloid leukemia, colorectal, pancreatic, bladder, and non-small lung cancer. The association of the genes with the PI3K-Akt signaling pathway and the regulation of pluripotent stem cells also reflect the importance of these somatic mutations. Lastly, the analysis also reported viral-associated pathways such as hepatitis B, viral carcinogenesis, and Human T Lymphotropic Virus Type 1 (HTLV-1) infection, where the interplay between somatic mutations in genes and viral integration events come together to give a bigger picture represented by overall changes in the biological pathways.

Mutational signatures are well-categorized somatic mutations with distinct nucleotide substitutions. These signatures are often identified through principal-component analysis of the trinucleotide mutation context, with 96 possible combinations of the mutated nucleotide including the bases 5′ and 3′ to each site [33]. There are currently 30 mutational signatures listed in the Catalogue of Somatic Mutations in Cancer (COSMIC), where some of these signatures represent exposure to mutagens, errors in the DNA replication machinery, or defective DNA repair [85].

Fujimoto et al. (2016) was able to identify seven distinct mutational signatures (W1-W7) in HCC patients. Three of the seven signatures (W1, W4, and W5) were found in multiple studies [33, 50, 55]. These recurrent signatures correspond well to COSMIC Signature 1, Signature 4, and Signature 16, which are proposed to be caused by the spontaneous deamination of 5-methylcytosine, tobacco mutagens, or unknown factors, respectively [85]. Other COSMIC signatures identified include Signature 9, Signature 12, and Signature 19, which are linked to somatic hypermutation, liver cancer, and unknown factors, respectively [86]. Signature W6 was not associated with any COSMIC signatures and, thus, represents a new mutational signature. A further meta-analysis performed by Letouze et al. (2017) identified 10 mutational signatures including COSMIC Signatures 1, 4, 5, 6, 12, 16, 17, 22, 23, and 24 [51]. A mutational signature characterized with increased C>A transversions was a major contributor to the driver mutations found in HCC patients exposed to aflatoxin B1 [40]. A high proportion of Taiwanese HCC patients marked with aristolochic acid mutagen exposure had T>A mutations that corresponded to COSMIC signature 22 [47]. The AA signature was also found to be higher in HCC patients from China and Southeast Asia and much lower in Japan, America, and Europe. A prominent mutational signature was also identified after cisplatin treatment in human liver cancer cell line HepG2 [87]. Mutational signatures not only allow us to appreciate the mechanisms underlying somatic mutations in HCC tumors but they could relate to mutational processes in other cancer types with related etiology.

Multi-omics analysis combine results from more than one type of data to give us a more comprehensive view of biological profiles. Boyault et al. (2007) conducted an unsupervised transcriptome analysis to identify six subgroups of HCC, G1-G6, where G1-G3 are associated with chromosomal instability, G5-G6 are related to β-catenin mutations, and G4 is a heterogenous group [88]. The association between HCC transcriptome subclasses, G5-G6, involved in Wnt pathway activation and *CTNNB1* mutations has been validated using WXS data in a later study [48, 88]. In addition, multi-omics analysis shows that there is a correlation between gene expression profiles from RNA-seq data and allele frequencies of somatic mutations from WGS, highlighting 252 genomic mutations that cause transcriptomic aberrations [37].

With the large number of available NGS-based HCC studies, there is an opportunity to integrate data across studies to provide greater statistical power and elimination of potential biases from a single cohort study. Zhang et al. (2014) collected four datasets containing 99, 88, 10, and 10 HCC samples to identify known and also novel mutated genes and pathways [89]. This study illustrated that larger sample sizes can identify mutations at lower frequencies in HCC than in smaller sample cohorts. As a second example of data integration, using combined liver cancer data from ICGC and TCGA to analyze the association of ancestry to HCC mutational signatures, an increase in T>C substitutions (in the ATA context) in Japanese males and an increase in T>A substitutions (in the CTG context) in US-Asian males and females were also reported [55].

## Future

### Mutations in the non-coding regulatory regions of the genome

Non-coding DNA makes up more than 98 percent of the human genome and include crucial transcription factor binding sites that regulate the transcription of RNA. Non-coding RNA includes introns, 3′ and 5′ UTR located in pre-mRNAs as well as microRNAs and long non-coding RNAs (lincRNAs) [90, 91]. The functional annotation of non-coding elements from the Encyclopedia of DNA Elements consortium and the US National Institutes of Health Roadmap Epigenomics project have provided support for the study of non-coding regions of human DNA [92, 93]. Cancer whole-genome data from TCGA have been intensively analyzed to identify mutations in the non-coding regions. For example, two pan-cancer studies have shown that *TERT* promoter mutations are present in at least six cancer types including glioblastoma, bladder, low-grade glioma, melanoma, and lung (and liver which is analyzed in one of the studies) [68, 94].

*TERT* promoter mutations are detected in 254 of 469 cases of HCC (54%) and more frequently detected in HCV-positive and non-viral cases than HBV-positive cases [55]. A more in-depth study reveals other noncoding mutations in *NEAT1, MALAT1, WDR*74 promoter, *BCL6* promoter, and *TFPI2* promoter [33]. Non-coding DNA analysis is challenging because many of the non-coding mutations are reported at lower mutation frequencies and at DNA locus with limited information regarding its function. We may overcome limitations in sample size and statistical power of patient datasets by analyzing an increased number of liver cancer whole genomes. Hence, there is potential to better characterize non-coding regions in the future.

### AAV2 viral integration events

In addition to HBV integration, recent reports of the observation of integration of the wild-type adeno-associated virus 2 (AAV2) in 11 of 193 cases of HCC via deep sequencing [49, 95] have sparked a debate regarding the safety issues of using AAV2 as a gene delivery vector in gene therapy [96–99]. Coincidently, the AAV2 integrations were detected in several recurrent mutation sites in HCC including the *TERT* promoter, *MLL4, CCNE1, CCNA2*, and *TNFSF10* [49, 100].

In an independent study, Fujimoto et al. (2016) detected AAV genome sequences in three liver cancer and three non-cancer liver cases. These three liver cancer cases were also infected with either HBV or HCV, and the AAV2 integration sites were located at *MLL4, CCNE1*, and an intergenic region of chromosome 5, respectively [33]. HBV integration sites were detected at the *CCNA2 locus* in one patient in this study as well as an early, well-differentiated HCC patient [12]. With these observations, additional analyses are necessary to evaluate the prevalence and effects of AAV2 integration events in liver cancer and in gene therapy. The extensiveness of WGS data is therefore applicable to the detection of foreign genomic material present in the human genome that may influence the development and the treatment of liver cancer.

### RNA editing

RNA editing caused by the deamination of nucleotide bases on an RNA sequence is catalyzed by the nucleotide-specific deaminases. Historically, transgenic mice and rabbits expressing mRNA editing enzyme APOBEC-1 (C-to-U editing) resulted in unexpected liver dysplasia, with a few of the mice developing HCC [101]. The main form of RNA editing is A-to-I editing catalyzed by the adenosine deaminase acting on RNA (ADAR) (A-to-I editing) family [102].

A genome-wide study that used both WGS and RNA-seq data reported normal and tumor-specific RNA editing sites in HCC as well as the positive correlation between editing degree ratio and gene expression ratio [39]. Results show that the increased expression of ADAR1 resulted in the over-editing of the *AZIN1* gene in HCC tumors, confirming the findings from a previous study [103]. Another genome-wide study showed that in addition to *AZIN1*, the *BLCAP* RNA has been over-edited (A-to-I editing) in HCC, and functional analysis suggests that the over-edited *BLCAP* resulted in enhanced cell proliferation and the activation of the AKT/mTOR signal pathway [104]. Two pan-cancer studies involving A-to-I RNA editing using data from TCGA reported no significant differences between matched normal and tumor samples, although a high Alu editing index in HCC has been significantly associated with poor survival [105, 106].

### Expanding the cancer genome database

With rapidly falling costs and newer technologies, the number of whole genomes sequenced in the next 10 years is projected to increase dramatically [107]. Larger sample sizes will provide better statistical power to detect rare variants and subgroups of liver cancer, particularly in HCC. For example, a large-scale whole-genome study conducted on the Icelandic population identified missense SNP variants in *ABCB4* to be associated with gallstone disease, liver cancer, liver cirrhosis, and other liver-specific traits [108, 109]. There are currently several international collaborations to generate more cancer whole genomes. The Pan-Cancer Analysis of Whole Genomes is an international collaboration between ICGC and TCGA to analyze more than 2,800 whole genomes across different cancer types to identify genetic alterations, beginning with 12 tumor types profiled by TCGA, although HCC was not included [110]. Additionally, the 100,000 Genomes Project by Genomics England in the United Kingdom will consist of samples from 25,000 cancer patients [111].

## Conclusion

In this review, we have discussed the key findings from WGS information (Fig. 1) and future directions of HCC. WGS is a promising approach that provides genomic information for discovery-based genomic analyses in the future. Hence, it holds great potential for liver cancer research as we seek to understand more about the genetic characteristics of HCC, which is influenced by gender, ethnicity, geolocation, and many risk factors. This review identified genes with somatic mutations (Table 2), many of which are involved in cancer-related pathways (Fig. 2). Many of the mutated genes are yet to be characterized for their molecular function and roles in cancer, presenting great opportunity for future research in this direction. With improved clinical annotation and the automation of data analysis, more genomic sequences can be translated into valuable biological insights.

## Abbreviations

AAV2: adeno-associated virus 2; COSMIC: Catalogue of Somatic Mutations in Cancer; DAVID: Database for Annotation, Visualization and Integrated Discovery; HBV: hepatitis B virus; HCC: hepatocellular carcinoma; HCV: hepatitis C virus; ICGC: International Cancer Genome Consortium; indel: insertions and deletions; KEGG: Kyoto Encyclopedia of Genes and Genomes; NGS: next-generation sequencing; RNA-seq: RNA sequencing; SNP: single nucleotide polymorphism; SNV: single-nucleotide variant; SRA: Sequence Read Archive; TCGA: the Cancer Genome Atlas; TERT: telomerase reverse transcriptase; WGS: whole-genome sequencing; WXS: whole-exome sequencing; EMBL-EBI: European Molecular Biology Laboratory - European Bioinformatics Institute;

### Competing interests

The authors declare that they have no competing interests.

### Funding

This work was supported by a grant from the Singapore Ministry of Health's National Medical Research Council (NMRC/CBRG/0095/2015) as well as some block funding from the National Cancer Centre Singapore and Duke-NUS Graduate Medical School to C.G.L. The funders had no role in study design, data collection and analysis, decision to publish, or preparation of the manuscript.

### Author contributions

Conceptualization: C.L., A.S.; data curation: W.H.; formal analysis: W.H.; funding acquisition: C.L.; supervision: C.L.; writing the original draft: .W.H; review and editing: C.L., A.S., W.H.

## Supplementary Material

GIGA-D-18-00339_Original_Submission.pdf

GIGA-D-18-00339_Revision_1.pdf

GIGA-D-18-00339_Revison_2.pdf

Response_to_Reviewer_Comments_Original_Submission.pdf

Response_to_Reviewer_Comments_Revision_1.pdf

Reviewer_1_Report_(Original_Submission) -- Eric Letouze 8/10/2018 Reviewed

## References

[1] Ferlay J, Soerjomataram I, Ervik M GLOBOCAN 2012 v1.0, Cancer Incidence and Mortality Worldwide: IARC CancerBase No. 11. http://globocan.iarc.fr 2013 Accessed 19 May 2018.

[2] El-Serag HB, Rudolph KL Hepatocellular carcinoma: epidemiology and molecular carcinogenesis. Gastroenterology. 2007;132(7):2557–76. 17570226 10.1053/j.gastro.2007.04.061

[3] El-Serag HB Hepatocellular carcinoma. N Engl J Med. 2011;365(12):1118–27. 21992124 10.1056/NEJMra1001683

[4] El-Serag HB Epidemiology of viral hepatitis and hepatocellular carcinoma. Gastroenterology. 2012;142(6):1264–73 e1. 22537432 10.1053/j.gastro.2011.12.061 PMC3338949

[5] Di Bisceglie AM, Simpson LH, Lotze MT Development of hepatocellular carcinoma among patients with chronic liver disease due to hepatitis C viral infection. J Clin Gastroenterol. 1994;19(3):222–6. 7528758 10.1097/00004836-199410000-00011

[6] Takano S, Yokosuka O, Imazeki F, Incidence of hepatocellular carcinoma in chronic hepatitis B and C: a prospective study of 251 patients. Hepatology (Baltimore, Md). 1995;21(3):650–5. 7875662

[7] Chang MH, Chen CJ, Lai MS, Universal hepatitis B vaccination in Taiwan and the incidence of hepatocellular carcinoma in children. Taiwan Childhood Hepatoma Study Group. N Engl J Med. 1997;336(26):1855–9. 9197213 10.1056/NEJM199706263362602

[8] Aspinall EJ, Hawkins G, Fraser A Hepatitis B prevention, diagnosis, treatment and care: a review. Occup Med (Lond). 2011;61(8):531–40. 22114089 10.1093/occmed/kqr136

[9] Paterlini-Brechot P, Saigo K, Murakami Y Hepatitis B virus-related insertional mutagenesis occurs frequently in human liver cancers and recurrently targets human telomerase gene. Oncogene. 2003;22(25):3911–6. 12813464 10.1038/sj.onc.1206492

[10] Tamori A, Yamanishi Y, Kawashima S, Alteration of gene expression in human hepatocellular carcinoma with integrated hepatitis B virus DNA. Clinical Cancer Research. 2005;11(16):5821–6. 16115921 10.1158/1078-0432.CCR-04-2055

[11] Nagaya T, Nakamura T, Tokino T The mode of hepatitis B virus DNA integration in chromosomes of human hepatocellular carcinoma. Genes & Development. 1987;1(8):773–82. 2828171 10.1101/gad.1.8.773

[12] Wang J, Chenivesse X, Henglein B Hepatitis B virus integration in a cyclin A gene in a hepatocellular carcinoma. Nature. 1990;343(6258):555–7. 1967822 10.1038/343555a0

[13] Dejean A, Bougueleret L, Grzeschik KH Hepatitis B virus DNA integration in a sequence homologous to v-erb-A and steroid receptor genes in a hepatocellular carcinoma. Nature. 1986;322(6074):70–2. 3014347 10.1038/322070a0

[14] Satoh S, Daigo Y, Furukawa Y, AXIN1 mutations in hepatocellular carcinomas, and growth suppression in cancer cells by virus-mediated transfer of AXIN1. Nat Genet. 2000;24(3):245–50. 10700176 10.1038/73448

[15] Murakami Y, Hayashi K, Hirohashi S, Aberrations of the tumor suppressor p53 and retinoblastoma genes in human hepatocellular carcinomas. Cancer Res. 1991;51(20):5520–5. 1655254

[16] Goodwin S, McPherson JD, McCombie WR Coming of age: ten years of next-generation sequencing technologies. Nat Rev Genet. 2016;17(6):333–51. 27184599 10.1038/nrg.2016.49 PMC10373632

[17] Jiang Z, Jhunjhunwala S, Liu J The effects of hepatitis B virus integration into the genomes of hepatocellular carcinoma patients. Genome Res. 2012;22(4):593–601. 22267523 10.1101/gr.133926.111 PMC3317142

[18] Sung WK, Zheng H, Li S Genome-wide survey of recurrent HBV integration in hepatocellular carcinoma. Nat Genet. 2012;44(7):765–9. 22634754 10.1038/ng.2295

[19] Toh ST, Jin Y, Liu L Deep sequencing of the hepatitis B virus in hepatocellular carcinoma patients reveals enriched integration events, structural alterations and sequence variations. Carcinogenesis. 2013;34(4):787–98. 23276797 10.1093/carcin/bgs406

[20] Tao Y, Ruan J, Yeh SH Rapid growth of a hepatocellular carcinoma and the driving mutations revealed by cell-population genetic analysis of whole-genome data. PNAS. 2011;108(29):12042–7. 21730188 10.1073/pnas.1108715108 PMC3141952

[21] Totoki Y, Tatsuno K, Yamamoto S, High-resolution characterization of a hepatocellular carcinoma genome. Nat Genet. 2011;43(5):464–9. 21499249 10.1038/ng.804

[22] Li M, Zhao H, Zhang X, Inactivating mutations of the chromatin remodeling gene ARID2 in hepatocellular carcinoma. Nat Genet. 2011;43(9):828–9. 21822264 10.1038/ng.903 PMC3163746

[23] Schulze K, Nault JC, Villanueva A Genetic profiling of hepatocellular carcinoma using next-generation sequencing. J Hepatol. 2016;65(5):1031–42. 27262756 10.1016/j.jhep.2016.05.035

[24] Leinonen R, Sugawara H, Shumway M The sequence read archive. Nucleic Acids Res. 2011;39(Database Issue):D19–21. 21062823 10.1093/nar/gkq1019 PMC3013647

[25] EBML-EBI European Nucleotide Archive (ENA) http://www.ebi.ac.uk/ena 2018 Accessed 31 Oct 2018.

[26] Kaminuma E, Mashima J, Kodama Y, DDBJ launches a new archive database with analytical tools for next-generation sequence data. Nucleic Acids Res. 2010;38(Database Issue):D33–8. 19850725 10.1093/nar/gkp847 PMC2808917

[27] National Cancer Institute - Genomic Data Commons. https://gdc.cancer.gov/ 2018 Accessed 31 Oct 2018.

[28] Gigadb. http://gigadb.org/ 2018 Accessed 31 Oct 2018.

[29] Sneddon TP, Li P, Edmunds SC GigaDB: announcing the GigaScience database. GigaScience. 2012;1(1):1–2. 23587345 10.1186/2047-217X-1-11 PMC3626507

[30] Kan Z, Zheng H, Liu X Hepatocellular carcinoma genomic data from the Asian Cancer Research Group. GigaScience. 2012.

[31] International Cancer Genome Consortium (ICGC). http://icgc.org/ 2018 Accessed 31 Oct 2018.

[32] Hudson TJ, Anderson W, Artez A International network of cancer genome projects. Nature. 2010;464(7291):993–8. 20393554 10.1038/nature08987 PMC2902243

[33] Fujimoto A, Furuta M, Totoki Y Whole-genome mutational landscape and characterization of noncoding and structural mutations in liver cancer. Nat Genet. 2016;48(5):500–9. 27064257 10.1038/ng.3547

[34] Fujimoto A, Furuta M, Shiraishi Y Whole-genome mutational landscape of liver cancers displaying biliary phenotype reveals hepatitis impact and molecular diversity. Nat Commun. 2015;6:6120 doi:10.1038/ncomms7120. 25636086 10.1038/ncomms7120

[35] Fujimoto A, Totoki Y, Abe T, Whole-genome sequencing of liver cancers identifies etiological influences on mutation patterns and recurrent mutations in chromatin regulators. Nat Genet. 2012;44(7):760–4. 22634756 10.1038/ng.2291

[36] Hirotsu Y, Zheng TH, Amemiya K Targeted and exome sequencing identified somatic mutations in hepatocellular carcinoma. Hepatology Research. 2016; doi:10.1111/hepr.12663. 10.1111/hepr.12663 26850916

[37] Shiraishi Y, Fujimoto A, Furuta M, Integrated analysis of whole genome and transcriptome sequencing reveals diverse transcriptomic aberrations driven by somatic genomic changes in liver cancers. PLoS One. 2014;9(12):e114263. 25526364 10.1371/journal.pone.0114263 PMC4272259

[38] Huang J, Deng Q, Wang Q Exome sequencing of hepatitis B virus-associated hepatocellular carcinoma. Nat Genet. 2012;44(10):1117–21. 22922871 10.1038/ng.2391

[39] Kang L, Liu X, Gong Z, Genome-wide identification of RNA editing in hepatocellular carcinoma. Genomics. 2015;105(2):76–82. 25462863 10.1016/j.ygeno.2014.11.005

[40] Zhang W, He H, Zang M Genetic features of aflatoxin-associated hepatocellular carcinoma. Gastroenterology. 2017;153(1):249–62.e2. 28363643 10.1053/j.gastro.2017.03.024

[41] Fernandez-Banet J, Lee NP, Chan KT, Decoding complex patterns of genomic rearrangement in hepatocellular carcinoma. Genomics. 2014;103(2–3):189–203. 24462510 10.1016/j.ygeno.2014.01.003

[42] Kan Z, Zheng H, Liu X Whole-genome sequencing identifies recurrent mutations in hepatocellular carcinoma. Genome Res. 2013;23(9):1422–33. 23788652 10.1101/gr.154492.113 PMC3759719

[43] Ahn SM, Jang SJ, Shim JH, Genomic portrait of resectable hepatocellular carcinomas: implications of RB1 and FGF19 aberrations for patient stratification. Hepatology (Baltimore, Md). 2014;60(6):1972–82. 10.1002/hep.27198 24798001

[44] Ouyang L, Lee J, Park CK Whole-genome sequencing of matched primary and metastatic hepatocellular carcinomas. BMC Med Genomics. 2014;7:2. 24405831 10.1186/1755-8794-7-2 PMC3896667

[45] Woo HG, Kim SS, Cho H Profiling of exome mutations associated with progression of HBV-related hepatocellular carcinoma. PLoS One. 2014;9(12):e115152. 25521761 10.1371/journal.pone.0115152 PMC4270755

[46] Lin KT, Shann YJ, Chau GY Identification of latent biomarkers in hepatocellular carcinoma by ultra-deep whole-transcriptome sequencing. Oncogene. 2014;33(39):4786–94. 24141781 10.1038/onc.2013.424

[47] Ng AWT, Poon SL, Huang MN Aristolochic acids and their derivatives are widely implicated in liver cancers in Taiwan and throughout Asia. Sci Transl Med. 2017;9(412) doi:10.1126/scitranslmed.aan6446. 10.1126/scitranslmed.aan6446 29046434

[48] Guichard C, Amaddeo G, Imbeaud S Integrated analysis of somatic mutations and focal copy-number changes identifies key genes and pathways in hepatocellular carcinoma. Nat Genet. 2012;44(6):694–8. 22561517 10.1038/ng.2256 PMC3819251

[49] Nault JC, Datta S, Imbeaud S Recurrent AAV2-related insertional mutagenesis in human hepatocellular carcinomas. Nat Genet. 2015;47(10):1187–93. 26301494 10.1038/ng.3389

[50] Schulze K, Imbeaud S, Letouze E, Exome sequencing of hepatocellular carcinomas identifies new mutational signatures and potential therapeutic targets. Nat Genet. 2015;47(5):505–11. 25822088 10.1038/ng.3252 PMC4587544

[51] Letouzé E, Shinde J, Renault V, Mutational signatures reveal the dynamic interplay of risk factors and cellular processes during liver tumorigenesis. Nature Communications. 2017;8(1):1315. 10.1038/s41467-017-01358-x PMC5670220 29101368

[52] Cleary SP, Jeck WR, Zhao X, Identification of driver genes in hepatocellular carcinoma by exome sequencing. Hepatology (Baltimore, Md). 2013;58(5):1693–702. 10.1002/hep.26540 PMC3830584 23728943

[53] Jhunjhunwala S, Jiang Z, Stawiski EW, Diverse modes of genomic alteration in hepatocellular carcinoma. Genome Biol. 2014;15(8):436. 25159915 10.1186/s13059-014-0436-9 PMC4189592

[54] Chaudhary K, Poirion OB, Lu L, Multi-modal meta-analysis of 1494 hepatocellular carcinoma samples reveals significant impact of consensus driver genes on phenotypes. Clinical Cancer Research. 2018; doi:10.1158/1078-0432.ccr-18-0088. 10.1158/1078-0432.CCR-18-0088 PMC6542354 30242023

[55] Totoki Y, Tatsuno K, Covington KR, Trans-ancestry mutational landscape of hepatocellular carcinoma genomes. Nat Genet. 2014;46(12):1267–73. 25362482 10.1038/ng.3126

[56] Ding D, Lou X, Hua D Recurrent targeted genes of hepatitis B virus in the liver cancer genomes identified by a next-generation sequencing-based approach. PLoS Genet. 2012;8(12):e1003065. 23236287 10.1371/journal.pgen.1003065 PMC3516541

[57] Oba A, Shimada S, Akiyama Y, ARID2 modulates DNA damage response in human hepatocellular carcinoma cells. J Hepatol. 2017;66(5):942–51. 28238438 10.1016/j.jhep.2016.12.026

[58] Gao Q, Wang K, Chen K HBx protein-mediated ATOH1 downregulation suppresses ARID2 expression and promotes hepatocellular carcinoma. Cancer Sci. 2017;108(7):1328–37. 28498550 10.1111/cas.13277 PMC5497798

[59] Chen CL, Wang Y, Pan QZ Bromodomain-containing protein 7 (BRD7) as a potential tumor suppressor in hepatocellular carcinoma. Oncotarget. 2016;7(13):16248–61. 26919247 10.18632/oncotarget.7637 PMC4941311

[60] Zhang Q, Wei L, Yang H Bromodomain containing protein represses the Ras/Raf/MEK/ERK pathway to attenuate human hepatoma cell proliferation during HCV infection. Cancer Lett. 2016;371(1):107–16. 26620707 10.1016/j.canlet.2015.11.027

[61] Chiu YH, Lee JY, Cantley LC BRD7, a tumor suppressor, interacts with p85alpha and regulates PI3K activity. Mol Cell. 2014;54(1):193–202. 24657164 10.1016/j.molcel.2014.02.016 PMC4004185

[62] Yao S, Johnson C, Hu Q Differences in somatic mutation landscape of hepatocellular carcinoma in Asian American and European American populations. Oncotarget. 2016;7(26):40491–9. 27246981 10.18632/oncotarget.9636 PMC5130022

[63] Viatour P, Ehmer U, Saddic LA, Notch signaling inhibits hepatocellular carcinoma following inactivation of the RB pathway. J Exp Med. 2011;208(10):1963–76. 21875955 10.1084/jem.20110198 PMC3182062

[64] Anwar SL, Krech T, Hasemeier B, Deregulation of RB1 expression by loss of imprinting in human hepatocellular carcinoma. J Pathol. 2014;233(4):392–401. 24838394 10.1002/path.4376

[65] Zheng S, Cherniack AD, Dewal N Comprehensive Pan-genomic characterization of adrenocortical carcinoma. Cancer Cell. 2016;29(5):723–36. 27165744 10.1016/j.ccell.2016.04.002 PMC4864952

[66] Nagarajan N, Bertrand D, Hillmer AM Whole-genome reconstruction and mutational signatures in gastric cancer. Genome Biol. 2012;13(12):R115. 23237666 10.1186/gb-2012-13-12-r115 PMC4056366

[67] Dong H, Zhang L, Qian Z Identification of HBV-MLL4 integration and its molecular basis in Chinese hepatocellular carcinoma. PLoS One. 2015;10(4):e0123175. 25901726 10.1371/journal.pone.0123175 PMC4406717

[68] Fredriksson NJ, Ny L, Nilsson JA, Systematic analysis of noncoding somatic mutations and gene expression alterations across 14 tumor types. Nat Genet. 2014;46(12):1258–63. 25383969 10.1038/ng.3141

[69] Herrera-Abreu MT, Palafox M, Asghar U Early adaptation and acquired resistance to CDK4/6 Inhibition in estrogen receptor-positive breast cancer. Cancer Res. 2016;76(8):2301–13. 27020857 10.1158/0008-5472.CAN-15-0728 PMC5426059

[70] Scaltriti M, Eichhorn PJ, Cortes J, Cyclin E amplification/overexpression is a mechanism of trastuzumab resistance in HER2+ breast cancer patients. PNAS. 2011;108(9):3761–6. 21321214 10.1073/pnas.1014835108 PMC3048107

[71] Au-Yeung G, Lang F, Azar WJ, Selective targeting of cyclin E1-amplified high-grade serous ovarian cancer by cyclin-dependent kinase 2 and AKT inhibition. Clinical Cancer Research. 2017;23(7):1862–74. 27663592 10.1158/1078-0432.CCR-16-0620 PMC5364079

[72] Patch AM, Christie EL, Etemadmoghadam D Whole-genome characterization of chemoresistant ovarian cancer. Nature. 2015;521(7553):489–94. 26017449 10.1038/nature14410

[73] Ferber MJ, Montoya DP, Yu C Integrations of the hepatitis B virus (HBV) and human papillomavirus (HPV) into the human telomerase reverse transcriptase (hTERT) gene in liver and cervical cancers. Oncogene. 2003;22(24):3813–20. 12802289 10.1038/sj.onc.1206528

[74] Khoury JD, Tannir NM, Williams MD Landscape of DNA virus associations across human malignant cancers: analysis of 3,775 cases using RNA-Seq. J Virol. 2013;87(16):8916–26. 23740984 10.1128/JVI.00340-13 PMC3754044

[75] Hanahan D, Weinberg Robert A Hallmarks of cancer: the next generation. Cell. 2011;144(5):646–74. 21376230 10.1016/j.cell.2011.02.013

[76] Saigo K, Yoshida K, Ikeda R Integration of hepatitis B virus DNA into the myeloid/lymphoid or mixed-lineage leukemia (MLL4) gene and rearrangements of MLL4 in human hepatocellular carcinoma. Hum Mutat. 2008;29(5):703–8. 18320596 10.1002/humu.20701

[77] Park MH, Kim SY, Kim YJ, ALS2CR7 (CDK15) attenuates TRAIL induced apoptosis by inducing phosphorylation of survivin Thr34. Biochem Biophys Res Commun. 2014;450(1):129–34. 24866247 10.1016/j.bbrc.2014.05.070

[78] Tanaka S, Sugimachi K, Yamashita Yi Y, Tie2 vascular endothelial receptor expression and function in hepatocellular carcinoma. Hepatology (Baltimore, Md). 2002;35(4):861–7. 10.1053/jhep.2002.32535 11915032

[79] Tanaka S, Mori M, Sakamoto Y, Biologic significance of angiopoietin-2 expression in human hepatocellular carcinoma. J Clin Invest. 1999;103(3):341–5. 9927494 10.1172/JCI4891 PMC407900

[80] Mitsuhashi N, Shimizu H, Ohtsuka M Angiopoietins and Tie-2 expression in angiogenesis and proliferation of human hepatocellular carcinoma. Hepatology (Baltimore, Md). 2003;37(5):1105–13. 10.1053/jhep.2003.50204 12717391

[81] Sugimachi K, Tanaka S, Taguchi K Angiopoietin switching regulates angiogenesis and progression of human hepatocellular carcinoma. J Clin Pathol. 2003;56(11):854–60. 14600132 10.1136/jcp.56.11.854 PMC1770094

[82] Wada H, Nagano H, Yamamoto H Expression pattern of angiogenic factors and prognosis after hepatic resection in hepatocellular carcinoma: importance of angiopoietin-2 and hypoxia-induced factor-1 alpha. Liver International. 2006;26(4):414–23. 16629644 10.1111/j.1478-3231.2006.01243.x

[83] Huang da W, Sherman BT, Lempicki RA Systematic and integrative analysis of large gene lists using DAVID bioinformatics resources. Nat Protoc. 2009;4(1):44–57. 19131956 10.1038/nprot.2008.211

[84] Huang da W, Sherman BT, Lempicki RA Bioinformatics enrichment tools: paths toward the comprehensive functional analysis of large gene lists. Nucleic Acids Res. 2009;37(1):1–13. 19033363 10.1093/nar/gkn923 PMC2615629

[85] Forbes SA, Beare D, Boutselakis H, COSMIC: somatic cancer genetics at high-resolution. Nucleic Acids Res. 2017;45(D1):D777–D83. 27899578 10.1093/nar/gkw1121 PMC5210583

[86] Alexandrov LB, Nik-Zainal S, Wedge DC Signatures of mutational processes in human cancer. Nature. 2013;500(7463):415–21. 23945592 10.1038/nature12477 PMC3776390

[87] Boot A, Huang MN, Ng AWT In-depth characterization of the cisplatin mutational signature in human cell lines and in esophageal and liver tumors. Genome Res. 2018;28(5):654–65. 29632087 10.1101/gr.230219.117 PMC5932606

[88] Boyault S, Rickman DS, de Reynies A Transcriptome classification of HCC is related to gene alterations and to new therapeutic targets. Hepatology (Baltimore, Md). 2007;45(1):42–52. 10.1002/hep.21467 17187432

[89] Zhang Y, Qiu Z, Wei L Integrated analysis of mutation data from various sources identifies key genes and signaling pathways in hepatocellular carcinoma. PLoS One. 2014;9(7):e100854. 24988079 10.1371/journal.pone.0100854 PMC4079600

[90] Ghidini M, Braconi C Non-coding RNAs in primary liver cancer. Front Med (Lausanne). 2015;2:36. 26131450 10.3389/fmed.2015.00036 PMC4469108

[91] He Y, Meng XM, Huang C, Long noncoding RNAs: novel insights into hepatocelluar carcinoma. Cancer Lett. 2014;344(1):20–7. 24183851 10.1016/j.canlet.2013.10.021

[92] The-ENCODE-Project-Consortium. An integrated encyclopedia of DNA elements in the human genome. Nature. 2012;489(7414):57–74. 22955616 10.1038/nature11247 PMC3439153

[93] Bernstein BE, Stamatoyannopoulos JA, Costello JF The NIH roadmap epigenomics mapping consortium. Nat Biotechnol. 2010;28(10):1045–8. 20944595 10.1038/nbt1010-1045 PMC3607281

[94] Weinhold N, Jacobsen A, Schultz N, Genome-wide analysis of noncoding regulatory mutations in cancer. Nat Genet. 2014;46(11):1160–5. 25261935 10.1038/ng.3101 PMC4217527

[95] Nault J-C, Datta S, Imbeaud S Adeno-associated virus type 2 as an oncogenic virus in human hepatocellular carcinoma. Molecular & Cellular Oncology. 2016;3(2):e1095271. 27308626 10.1080/23723556.2015.1095271 PMC4905403

[96] Berns KI, Byrne BJ, Flotte TR, Adeno-associated virus type 2 and hepatocellular carcinoma?. Hum Gene Ther. 2015;26(12):779–81. 26690810 10.1089/hum.2015.29014.kib PMC4809064

[97] Buning H, Schmidt M Adeno-associated vector toxicity-to be or not to be?. Mol Ther. 2015;23(11):1673–5. 26606658 10.1038/mt.2015.182 PMC4817949

[98] Gil-Farina I, Fronza R, Kaeppel C Recombinant AAV integration is not associated with hepatic genotoxicity in nonhuman primates and patients. Mol Ther. 2016; doi:10.1038/mt.2016.52. 10.1038/mt.2016.52 PMC4923321 26948440

[99] Schmidt M, Gil-Farina I, Buning H Reply to “Wild-type AAV insertions in hepatocellular carcinoma do not inform debate over genotoxicity risk of vectorized AAV.”. Mol Ther. 2016;24(4):661–2. 27081718 10.1038/mt.2016.48 PMC4886948

[100] Nault JC, Datta S, Imbeaud S AAV2 and hepatocellular carcinoma. Hum Gene Ther. 2016;27(3):211–3. 26935016 10.1089/hum.2016.002

[101] Yamanaka S, Balestra ME, Ferrell LD Apolipoprotein B mRNA-editing protein induces hepatocellular carcinoma and dysplasia in transgenic animals. PNAS. 1995;92(18):8483–7. 7667315 10.1073/pnas.92.18.8483 PMC41181

[102] Brennicke A, Marchfelder A, Binder S RNA editing. FEMS Microbiol Rev. 1999;23(3):297–316. 10371035 10.1111/j.1574-6976.1999.tb00401.x

[103] Chen L, Li Y, Lin CH, Recoding RNA editing of AZIN1 predisposes to hepatocellular carcinoma. Nat Med. 2013;19(2):209–16. 23291631 10.1038/nm.3043 PMC3783260

[104] Hu X, Wan S, Ou Y, RNA over-editing of BLCAP contributes to hepatocarcinogenesis identified by whole-genome and transcriptome sequencing. Cancer Lett. 2015;357(2):510–9. 25499081 10.1016/j.canlet.2014.12.006

[105] Ding SL, Yang ZW, Wang J Integrative analysis of aberrant Wnt signaling in hepatitis B virus-related hepatocellular carcinoma. World Journal of Gastroenterology. 2015;21(20):6317–28. 26034368 10.3748/wjg.v21.i20.6317 PMC4445110

[106] Paz-Yaacov N, Bazak L, Buchumenski I Elevated RNA editing activity is a major contributor to transcriptomic diversity in tumors. Cell Reports. 2015;13(2):267–76. 26440895 10.1016/j.celrep.2015.08.080

[107] Eisenstein M Big data: the power of petabytes. Nature. 2015;527(7576):S2–4. 26536222 10.1038/527S2a

[108] Gudbjartsson DF, Helgason H, Gudjonsson SA Large-scale whole-genome sequencing of the Icelandic population. Nat Genet. 2015;47(5):435–44. 25807286 10.1038/ng.3247

[109] Lammert F, Hochrath K A letter on ABCB4 from Iceland: on the highway to liver disease. Clin Res Hepatol Gastroenterol. 2015;39(6):655–8. 26410236 10.1016/j.clinre.2015.08.004

[110] Weinstein JN, Collisson EA, Mills GB The cancer genome atlas Pan-cancer analysis project. Nat Genet. 2013;45(10):1113–20. 24071849 10.1038/ng.2764 PMC3919969

[111] Marx V The DNA of a nation. Nature. 2015;524(7566):503–5. 26310768 10.1038/524503a

