# Supplementary material for: Advances in genomic hepatocellular carcinoma research
Source: Gigascience. 2018 Dec 6;7(12):giy135. doi: 10.1093/gigascience/giy135 (PMC6335342; doi:10.1093/gigascience/giy135)
Supplement: GIGA-D-18-00339_Revision_1.pdf [file giy135_giga-d-18-00339_revision_1.pdf]

GigaScience

Advances in Genomic Hepatocellular Carcinoma Research

--Manuscript Draft--

**Manuscript Number:**	GIGA-D-18-00339R1
**Full Title:**	Advances in Genomic Hepatocellular Carcinoma Research
**Article Type:**	Review
**Funding Information:**	National Medical Research Council (NMRC/CBRG/0095/2015) — Dr. Caroline G.L. Lee<br>National Cancer Centre of Singapore (Block funding) — Dr. Caroline G.L. Lee<br>Duke-NUS Graduate Medical School (Block funding) — Dr. Caroline G.L. Lee
**Abstract:**	Background: Hepatocellular carcinoma (HCC) is the cancer with the second highest mortality in the world due to its late presentation and limited treatment options. As such, there is an urgent need to identify novel biomarkers for early diagnosis and develop novel therapies. The availability of Next Generation Sequencing (NGS) data from tumors of liver cancer patients has provided us with invaluable resources to better understand HCC through the integration of data from different sources to facilitate the identification of promising biomarkers or therapeutic targets.<br><br>Main findings: Here, we review key insights gleaned from over 20 NGS studies of HCC tumor samples, comprising approximately 582 whole genomes and 1211 whole exomes mainly from the East Asian population. Through consolidation of reported somatic mutations from multiple studies, we identified genes with different types of somatic mutations including single nucleotide variations, insertion/deletions, structural variations and copy number alterations as well as genes with multiple frequent viral integration. Pathway analysis showed that this curated list of somatic mutations are critically involved in cancer-related pathways, viral carcinogenesis and signalling pathways. Lastly, we addressed the future directions of HCC research as more NGS datasets become available.<br><br>Conclusion: Our review is a comprehensive resource for the current NGS research in HCC consolidating published articles, potential gene candidates and their related biological pathways.
**Corresponding Author:**	Caroline G.L. Lee, Ph.D<br>National University Singapore Yong Loo Lin School of Medicine<br>Singapore, SINGAPORE
**Corresponding Author Secondary Information:**	
**Corresponding Author's Institution:**	National University Singapore Yong Loo Lin School of Medicine
**Corresponding Author's Secondary Institution:**	
**First Author:**	Weitai HUANG
**First Author Secondary Information:**	
**Order of Authors:**	Weitai HUANG<br>Anders Jacobsen SKANDERUP, Ph.D<br>Caroline G.L. Lee, Ph.D
**Order of Authors Secondary Information:**	
**Response to Reviewers:**	Point-by-point Response Letter<br><br>Reviewer 1, Summary<br>Reviewer comment

Powered by Editorial Manager® and ProduXion Manager® from Aries Systems Corporation

In this work, Huang et al. review and summarize most of the NGS studies related to HCC in the last few years.

The study is nicely conducted and well written, and will be very useful to easily access and integrate the high number of data sets generated by many teams around the world.
I have a few comments below.

Author response:
We would like to thank our reviewer for his appreciation of our work and for providing constructive suggestions to improve the manuscript.

Reviewer 1, Comment 1
Reviewer comment
The following references are missing and should be added :
- Zhang et al., Gastroenterology 2017, 49 WGS of aflatoxin B1 -related HCC: https://www.ncbi.nlm.nih.gov/pubmed/28363643
- Ng et al., Sci Transl Med 2017, WES of 98 HCC from Taiwan with a high contribution of aristolochic acid:
https://www.ncbi.nlm.nih.gov/pubmed/29046434
- Letouzé et al., Nat Commun 2017, WGS of 45 liver cancers from Europe with diverse etiological backgrounds:
https://www.ncbi.nlm.nih.gov/pubmed/29101368
- Chaudhary et al., Clin Cancer Res 2018, Meta-analysis of 1,494 HCC:
https://www.ncbi.nlm.nih.gov/pubmed/30242023

Author response:
We have cited and discussed about the above articles in the text of the manuscript as well as added the information to Table 1 and Table 2 accordingly.

Reviewer 1, Comment 2
Reviewer comment
The precise criteria used by the authors to include a gene in Table 2 should be specified, especially if the authors present this list as a reference of HCC driver genes. I would suggest to order genes according to a criteria (e.g. alteration frequency in HCC) to see the main drivers in the first lines.

Also, the reason why the authors discuss in detail ALB, ARID2, RB1, BRD7 and RPL22 rather than other genes is not clear to me.

Finally, although several genes harbor both mutations, SVs and CNAs, it may be worth to present separately genes that are primarily affected by SVs and CNAs (e.g. FGF19/CCND1, CCNE1, CDKN2A…). Otherwise they are a bit "drowned" in the sea of candidate drivers from mutations.

Author response:
Noting that different types of mutations (SNVs, indels, SVs and CNAs) can be caused by different mechanisms, we are interested to identify HCC driver genes affected by more than one type of mutation.

In this order, it has brought to our attention that ALB, ARID2, RB1, BRD7 and RPL22 represent a set of HCC driver genes that are (1) affected by different mechanisms including SVs (5 out of 23) and CNAs (5 out of 10), (2) reported in multiple independent studies and (3) gives more appreciation to HCC driver genes found to harbor SVs and CNAs as compared to the well discussed HCC driver genes (TP53, CTNNB1 and AXIN1).

To help readers appreciate genes primarily affected by SVs and CNAs, we have added colour-tagged boxes to quickly differentiate them from SNVs and indels.

Reviewer 1, Comment 3
Reviewer comment
It has been shown that very highly expressed genes (like ALB or APOB) display a striking accumulation of indels, likely due to the collision of the transcription and

Powered by Editorial Manager® and ProduXion Manager® from Aries Systems Corporation

	replication machineries (Letouzé et al., Nat Commun 2017).<br><br>The authors should mention this alternate hypothesis to explain the recurrence of alterations in highly expressed liver genes.<br><br>Author response:<br>We thank this reviewer for highlighting this alternate hypothesis and we have included in the revised manuscript on (Page 7, lines 151-153).<br><br>Reviewer 1, Comment 4<br>Reviewer comment<br>HBV insertions in CCNA2 were also reported (Wang et al., Nature 1990 and Fujimoto et al., Nat Genet 2016) and should be mentioned.<br><br>Author response:<br>We have addressed these findings under the Future, "AAV2 viral integration events" section, (Page 13, lines 315-316).<br><br>Reviewer 1, Comment 5<br>Reviewer comment<br>Fujimoto et al. identified 7 signatures but 10 have been identified in a meta-analysis (Letouzé et al., Nat Commun 2017) and another signature related to cisplatin treatment was recently described (Boot et al., Genome Res 2018). I would suggest to mention these studies in the "mutational signature" paragraph, as well as the in-depth analyses of the aflatoxin B1 (Zhang et al., Gastroenterology 2017) and aristolochic acid (Ng et al., Sci Transl Med 2017) signatures.<br><br>Author response:<br>We have further elaborated in the "Mutational signature" paragraph with regards to these relevant findings important for our review (Page 11, lines 255-263).
**Additional Information:**	
**Question**	**Response**
Are you submitting this manuscript to a special series or article collection?	No
**Experimental design and statistics**<br><br>Full details of the experimental design and statistical methods used should be given in the Methods section, as detailed in our Minimum Standards Reporting Checklist. Information essential to interpreting the data presented should be made available in the figure legends.<br><br>Have you included all the information requested in your manuscript?	Yes
**Resources**<br><br>A description of all resources used, including antibodies, cell lines, animals and software tools, with enough information to allow them to be uniquely	Yes

identified, should be included in the Methods section. Authors are strongly encouraged to cite Research Resource Identifiers (RRIDs) for antibodies, model organisms and tools, where possible.<br><br>Have you included the information requested as detailed in our Minimum Standards Reporting Checklist?	
**Availability of data and materials**<br><br>All datasets and code on which the conclusions of the paper rely must be either included in your submission or deposited in publicly available repositories (where available and ethically appropriate), referencing such data using a unique identifier in the references and in the “Availability of Data and Materials” section of your manuscript.<br><br>Have you have met the above requirement as detailed in our Minimum Standards Reporting Checklist?	Yes

Manuscript
Click here to access/download;Manuscript;NGSReviewDraft_HW_r1cl_hw.doc
Click here to view linked References

Advances in Genomic Hepatocellular Carcinoma Research

Weitai HUANG[1,2,3], Anders Jacobsen SKANDERUP[1], Caroline G. LEE[2,3,4,5,6]

[1] Computational and Systems Biology, Agency for Science Technology and Research, Genome Institute of Singapore, 60 Biopolis Street, Singapore 138672, Singapore;
[2] Graduate School of Integrative Sciences and Engineering, National University of Singapore, 5 Lower Kent Ridge Road, Singapore 117456, Singapore;
[3]Department of Biochemistry, Yong Loo Lin School of Medicine, National University of Singapore, Singapore 119077, Singapore;
[4]Division of Medical Sciences, Humphrey Oei Institute of Cancer Research, National Cancer Center Singapore, Singapore 169610, Singapore;
[5]Duke-NUS Graduate Medical School Singapore, Singapore 169547, Singapore

[6]**Corresponding author.**

EMAIL caroline_lee@nuhs.edu.sg; **TEL** (65) 6436-8353; **FAX** (65) 6372-0161.

Abstract

Background: Hepatocellular carcinoma (HCC) is the cancer with the second highest mortality in the world due to its late presentation and limited treatment options. As such, there is an urgent need to identify novel biomarkers for early diagnosis and develop novel therapies. The availability of Next Generation Sequencing (NGS) data from tumors of liver cancer patients has provided us with invaluable resources to better understand HCC through the integration of data from different sources to facilitate the identification of promising biomarkers or therapeutic targets.

Main findings: Here, we review key insights gleaned from over 20 NGS studies of HCC tumor samples, comprising approximately 582 whole genomes and 1211 whole exomes mainly from the East Asian population. Through consolidation of reported somatic mutations from multiple studies, we identified genes with different types of somatic mutations including single nucleotide variations, insertion/deletions, structural variations and copy number alterations as well as genes with multiple frequent viral integration. Pathway analysis showed that this curated list of somatic mutations is critically involved in cancer-related pathways, viral carcinogenesis and signalling pathways. Lastly, we addressed the future directions of HCC research as more NGS datasets become available.

Conclusion: Our review is a comprehensive resource for the current NGS research in HCC consolidating published articles, potential gene candidates and their related biological pathways.

Keywords: Hepatocellular Carcinoma, Next-generation Sequencing, Somatic Mutations, Viral Integration

Introduction

Based on GLOBOCAN 2012, liver cancer is the second most common cause of death from cancer worldwide. Liver cancer is the 5th most common cancer in males (554,000 cases) and 9th most common cancer in females (228,000 cases) [1]. The incidence rate is higher in males than females at a male-to-female ratio of 2.4 worldwide and the mortality-to-incidence rate is as high as 0.94 and 0.98 for males and females respectively. Hepatocellular carcinoma (HCC) is the most dominant form of primary liver cancer. Geographically, there is a high incidence rate in Africa (Northern and Western) and Asia (Eastern and Southeast), particularly in China which accounts for 50 percent of all HCC cases [2].

HCC is commonly associated with risk factors such as hepatitis B (HBV), hepatitis C (HCV) infection, alcohol, mycotoxin Aflatoxin, obesity and non-alcoholic fatty liver disease; and the risk varies depending on gender, geographic region and ethnicity [2-4]. Early evidence shows the association of HBV and HCV infection to the development of liver cirrhosis and HCC [5, 6]. HBV vaccine is available since early 1980s and implementation of HBV vaccination programs in 177 of 193 WHO member states are successful in decreasing HCC incidence rates in children [7, 8].

While environmental factors play a role in HCC, multiple recurrent genetic aberrations and the disruption of the host genome due to HBV DNA integration in HBV-associated HCC are reported to cause the dysregulation of genes important for the hallmarks of cancer. Initial studies identified HBV integration sites via HBV DNA probes or PCR followed by Sanger sequencing [9-13]. Subsequently, somatic alterations such as mutations, gene copy number changes and chromosomal rearrangements detected in the HCC-derived cell lines were found to affect the expression of oncogenes and tumor suppressor genes [14, 15]. Progress in the mapping of each viral integration site and genetic aberration in HCC patients, was ad-hoc and slow before the advent of Next generation sequencing (NGS).

NGS technologies, including RNA-sequencing (RNA-seq), whole exome sequencing (WXS) and whole genome sequencing (WGS), forms the foundation of today's discovery-based genomics research. With the reduced cost of massively parallel sequencing technologies over the last decade [16],

there has been an increasing number of genomic liver cancer studies providing new insights about liver cancer. Pioneering NGS studies conducted on patient samples have shown a tremendous leap in our understanding of HBV viral integration patterns [17-19] as well as somatic alterations found in liver cancer [20-22]. The large amount of sequencing data generated have been archived on data servers worldwide, enabling researchers to perform integrative analyses that would lead to new findings. However, maneuvering through literature and data repositories to locate and access these information remains a tedious process.

This review takes the opportunity to introduce and consolidate all existing NGS-based studies on liver cancer (Fig. 1). Only the most relevant studies, conducted using NGS in HCC, have been listed in a recent review [23]. Our NGS-based resource is a complete list of approximately 582 whole genomes and 1211 whole exomes data samples. It summarizes the key research and clinical findings from each article with direct links to all publicly available WGS/WXS liver cancer datasets to promote better knowledge and data facilitation. The key findings of somatic mutations, HBV integrations and mutational signatures reported from recent high-throughput studies and related integrative studies are discussed. We highlight key genes reported across multiple studies found to have recurrence of somatic mutations or HBV integration events. Additionally, we provide a meta-analysis of the pathways that these alterations dysregulate. Finally, we will discuss future directions and trends in liver cancer research via the analysis of high-throughput data.

NGS Resources

Raw sequencing data, read alignment and annotations from NGS platforms can be accessed via NCBI-Sequence Read Archive (SRA) (http://www.ncbi.nlm.nih.gov/sra), EBML-EBI European Nucleotide Archive (ENA) (http://www.ebi.ac.uk/ena) or DNA Data Bank of Japan-SRA (DRA) (http://www.ddbj.nig.ac.jp) [24, 25]. The National Cancer Institute's Genomic Data Commons (https://gdc.cancer.gov/) currently hosts genomic data from The Cancer Genome Atlas (TCGA) project that consist of multiple cancer types. There are currently 377 Liver Hepatocellular Carcinoma (LIHC)

samples with data from WXS, SNP-array, methylation, mRNA and microRNA profiling. Gigadb (http://gigadb.org/) is a repository for open-access data associated with the GigaScience journal [26] which currently holds a HCC dataset from 88 individuals [27]. The International Cancer Genome Consortium (ICGC) (http://icgc.org/) is a global effort to coordinate large-scale cancer genome studies by providing a comprehensive catalogue of somatic mutations across 50 cancer types which generates approximately 500 samples each [28]. While primary data files are stored on NCBI and/or EBI, ICGC provides interpreted datasets for somatic mutation calls as well as incorporate transcriptomic and DNA methylation analyses from the same tumor samples.

We reviewed and consolidated a comprehensive list of liver cancer studies, which have analysed high-throughput genomics data (Table 1). The majority of the studies have their raw and/or processed data available on the above-mentioned public databases (Table 1, Data URL). These studies are, mainly focused on liver cancer patients from a single country of the East Asian population (Table 1, Population). Genomics data from the Japanese population constitutes the largest sample size [21, 29-33], including a collection of 300 whole genomes reported in a recent study [29]. NGS studies were also performed with HCC patients from China [34-36], Hong Kong [18, 35, 37, 38], Korea [39-41], Taiwan [42, 43], Singapore [19] and Europe [44-47]. Several studies have a collection of samples from various ethnicities (TCGA) or multiple sources [17, 22, 48-50].

Multiple findings have already been reported on the patient samples from Japan [29-31], Hong Kong [18, 37, 38], Europe [44-47], as well as integrative studies from multiple sources [50, 51] or commercial sources [17, 49]. Here, we review approximately 582 whole genomes, 1211 exome and 778 RNA-sequencing samples of liver cancer patients (Table 1, Total cases). Of patients with known viral status, 44 percent are infected with HBV, 21 percent with HCV while 35 percent are not infected by either HBV or HCV (NBNC) (Table 1, Viral status). Several of the groups have also employed NGS to examine HBV integrations in HCC patients [17-19, 52].

Key findings

Somatic genomic alterations

By comparing matched normal and tumor samples, computational algorithms have identified a number of likely cancer-causing point mutations and insertions/deletions (indels). Somatic alterations such as point mutations, indels, structural variants and copy number alterations have been identified in one or more of the 85 genes that we have included in Table 2. Recurrent mutations in 12 genes (*TP53*, *CTNNB1*, *AXIN1*, *ALB*, *ARID2*, *ARID1A*, *RPS6KA3*, *APOB*, *RB1*, *CDKN2A*, *LRP1B* and *PTEN*) were reported in multiple studies. In this section, we will discuss five genes (*ALB*, *ARID2*, *RB1*, *BRD7*, and RPL22) which were reported to show all four types of somatic alterations. To gain further insights into the genes with reported somatic mutations, their gene expression (tumor/normal fold-change) and clinic-pathological clinical information (histologic grade and survival) from the TCGA HCC cohort are also presented.

ARID2 belongs to the SWI/SNF-related chromatin remodelling complexes, and is identified as a tumor suppressor that is frequently mutated in HCC patients [22, 38, 44]. In addition, gene expression profiling of *ARID2*-deficient HCC cell lines reveal negative regulation of UV-response gene sets suggesting that *ARID2* may be involved in DNA repair processes.[53]. *ARID2* is also involved in HCC via the effects of Hepatitis B and C infection. In HBV-related HCC, the HBV X protein is reported to suppress *ARID2* expression leading to increased hepatoma tumorigenesis [54]. *ARID2* mutations are also significantly associated (p=0.046) with HCV-related HCC [22]. These findings suggest that *ARID2* is a critical tumor suppressor in hepatitis virus related HCC progression.

Similar to *ARID2*, *BRD7* is also a component of the SWI/SNF remodelling machinery and a putative tumor suppressor reported with significant truncating mutations in HCC [51]. Loss of function mutations at the *BRD7* gene locus are frequently observed (7/268) in HBV-associated HCC patients

[29]. BRD7 expression is also reported to be associated with the clinical characteristics in HCC (tumor size, tumor stage and survival) [55]. HCV infections repress *BRD7* expression *in vitro* resulting in the dysregulation of hepatoma cell proliferation [56]. BRD7 also negatively regulate PI3K signalling by binding to the inter-SH2 (iSH2) domain of p85, leading to the impairment of p88/p110 complex formation [57].

The *ALB* gene encodes for the most abundant plasma protein, albumin, synthesized exclusively by hepatocytes [37]. Blood albumin tests that deviate from the normal healthy range often indicate dysregulation of protein production in the liver and other liver-associated issues. Somatic mutations at the *ALB* gene locus were reported in multiple studies including genomic rearrangements in 10% (9/88) of Chinese HCC patients [37] as well as point mutations clusters and indels in Japanese HCC patients [29]. *ALB* is touted as a liver cancer driver gene as it is significantly enriched with damaging mutations in the European population [46]. Highly expressed genes such as *ALB* and *APOB* have been shown to be strongly enriched with indels, which are characteristic of replication slippage errors resulting from conflicts between the replication and transcription machineries [47]. Hence, low albumin levels may contribute to liver cancer progression.

RB1 is a key inhibitor of cell cycle progression that harbours multiple nonsense mutations and genomic deletions in HCC patients [29, 38, 39, 46]. *RB1* is found to be predominantly mutated in Asian Americans (10/53 patients) as compared to European Americans (2/101 patients) [58]. The inactivation of RB pathway in Rb family triple knockout mice resulted in the development of HCC [59]. A study reveals that in 16/40 HCC patients, DNA methylation abnormalities were observed in CpG island 85 (CpG85) located within intron 2 of the *RB1* gene, which can potentially regulate the expression of the *RB1-E2B* alternative transcript [60]. In addition, *RB1* mutations are also significantly associated with reduced cancer-specific and recurrence-free survival after resection in HCC patients [39, 46]. It is thus worthwhile to further characterize *RB1* mutations, as they are reported to have a significantly higher mutation rate in HBV-related HCCs [38, 39].

RPL22, another gene that is reported to exhibit all 4 different types of mutations (SNV, indels, structural and copy number variation), encodes for a ribosomal 60S subunit protein. It was reported to be significantly mutated in Japanese (5/268 patients) and European (7/242) HCC patients [29, 46]. *RPL22* was identified through pan-genomic characterization, as a driver gene with significant somatic alterations in adenocortical carcinoma [61]. A study of microsatellite instability-positive gastric cancers also identified *RPL22* as a recurrently mutated gene with single base deletions [62]. Therefore, there is potential for more research to be conducted to fully determine the functional roles of *RPL22* in HCC.

HBV integration

The HBV genome often integrates into the chromosomes of liver cells resulting in alterations of the host genome. Recent findings have confirmed that the viral transcription/replication initiation site, DR1, (located near the 3' end of the *HBx* gene and the beginning of the Precore/Core gene) is the preferred region to be integrated into the host chromosome [11, 17, 19]. More HBV integration events were identified in tumor as compared to their matched normal samples [18]. In HCC tumors, studies show that HBV integration were randomly distributed throughout the human genome [17, 18, 29]. In a group of 48 HCC patients from the Singapore cohort, HBV integrations were significantly enriched in the q arm of chromosome 10 and correlated with poorly differentiated tumors [19].

From the NGS studies, we have consolidated a comprehensive table of viral integration events that occurred in HCC patients (Table 3). There are multiple integration events in the promoter, 3'UTR, coding sequence and/or intronic region of the *CCNE1* [63], *TERT* [19, 31, 33], *CDK15* [33], *ROCK1 [18]*, *FN1 [64]*, *APOA2* [63] and *MLL4* [17, 18, 63] genes. HBV was reported in several studies to integrate into the *CCNE1* and *TERT* genes [18, 29, 49]. *CDK15, ROCK1, FN1,APOA2* and *MLL4* are less frequently reported to be sites of integration for HBV.

CCNE1 encodes for the cyclin E1 protein that is a regulatory subunit of *CDK2* involved in G1/S phase of the cell cycle. *CCNE1* amplification have been reported to be the mechanism of resistance in *ER*-positive and *HER2*-positive breast cancers as well as high grade serous ovarian cancer [65-68]. HBV integrations within the *CCNE1* have been reported in four of 76 HBV-positive HCC samples and resulted in significantly increased expression of *CCNE1* [18]. The molecular mechanism of *CCNE1* mutations in HCC patients has yet to be fully elucidated.

The previously reported recurrent integration site at the *TERT* promoter was found by several high-throughput genomic studies to be the most frequent site for integration [19, 29, 69, 70]. Disruption of the *TERT* promoter is likely to cause the dysregulation of the telomerase reverse transcriptase (TERT) expression which plays important roles in cancer development due to its diverse telomere-independent functions in Wnt pathway signaling, cell proliferation and DNA-damage repair [71]. Viral sequences may act as enhancers where the closer the HBV is integrated to the transcription start site (TSS) of *TERT*, the higher the mRNA expression of TERT [19].

Chimeric *HBx/MLL4* fusion transcripts containing the *HBx* promoter and ORF fused to the exon 4 and 5 of *MLL4* were initially, detected in four out of ten HCC patients [72] and subsequently confirmed in later studies and reported to lead to increased *MLL4* expression [17, 18, 63]. In a Chinese cohort, 8 out of 44 patients were found to contain *HBx/MLL4* fusion transcripts, resulting in a higher expression of *MLL4* gene [63]. The chimeric transcript lacks the AT-hook DNA-binding domain of *MLL4*, hence it may act as a dominant negative allele [17].

CDK15 encodes for the cyclin-dependent kinase 15 and is a serine/threonine protein kinase. In one study, CDK15 contributes to the effects of tumor necrosis factor-related apoptosis-inducing ligand resistance by possibly regulating the phosphorylation of survivin (Thr34) [73]. Interestingly, multiple HBV-*CDK15* fusion transcripts were detected in an HCC patient, including one in-frame fusion, which caused CDK15 over-expression [33]. However, like many of the other genes where HBV integrations have been identified, the function of *CDK15* in HCC remains unclear. Hence, there is great potential to further investigate HBV integrations in HCC.

It is noteworthy that *CCNE1, TERT, and ANGPT1* not only harbour somatic mutations (Table 2), they are also reported to be sites for viral integrations (Table 3). *CCNE1* has been reported with structural variant alterations and HBV integrations while *TERT* has been reported with point mutations, structural variant alterations and HBV integrations suggesting that deregulation of these genes may play important roles in tumorigenesis. *ANGPT1* (Angiopoietin-1), a ligand for Tie2 vascular endothelial-specific receptor tyrosine kinase, involved in the induction of HCC neovascularization and disease progression [74-76], was reported to harbor point mutations and HBV integrations in its intronic regions. *ANGPT1* and Angiopoietin-2 (*ANGPT2*) were over-expressed in 68 and 81 percent of poorly differentiated HCC tumors respectively [77]. However, high ANGPT2 expression but not ANGPT1 showed correlation in the disease-free survival of 60 HCC patients [78]. Role of *ANGPT1* in tumor angiogenesis remains unclear.

Pathways of Somatic Mutated Genes and Mutation Signatures

Pathway analysis based on the Kyoto Encyclopedia of Genes and Genomes (KEGG) was performed using the Database for Annotation, Visualization and Integrated Discovery (DAVID v6.8) to identify pathways that were altered by somatic mutations in the TCGA HCC cohort [79, 80]. Seventy-nine of the 85 genes in our list of somatic mutations have identifiable DAVID IDs of which 45 genes can be categorized in KEGG pathways. Fifteen significant pathways were identified ($FDR < 0.05$) from the 45 genes, of which 14 genes are found to be involved in more than one of the pathways (Figure 2). All 14 genes are involved in Pathways in cancer, including other significant cancer types: prostate, endometrial, glioma, melanoma, chronic myeloid leukemia, colorectal, pancreatic, bladder as well as non-small lung cancer. The association of the genes with PI3K-Akt signaling pathway and the regulation of pluripotent stem cells also reflect the importance of these somatic mutations. Lastly, the analysis also reported viral-associated pathways such as Hepatitis B, viral carcinogenesis and HTLV-I

infection, where the inter-play between somatic mutations in genes and viral integration events come together to give a bigger picture represented by overall changes in the biological pathways.

Mutational signatures are well-categorized somatic mutations with distinct nucleotide substitutions. These signature are often identified through principal-component analysis of the trinucleotide mutation context, with 96 possible combinations of the mutated nucleotide including the bases 5' and 3' to each site [29]. There are currently 30 mutational signatures listed in the Catalogue of Somatic Mutations in Cancer (COSMIC), where some of these signatures represent exposure to mutagens, errors in the DNA replication machinery, or defective DNA repair [81].

Fujimoto et al. (2016) was able to identify seven distinct mutational signatures (W1-W7) in HCC patients. 3 of the 7 signatures (W1, W4 and W5) were found in multiple studies [29, 46, 51]. These recurrent signatures correspond well to COSMIC Signature 1, Signature 4 and Signature 16, which are proposed to be caused by the spontaneous deamination of 5-methylcytosine, tobacco mutagens or due to unknown factors respectively [81]. Other COSMIC signatures identified include Signature 9, Signature 12 and Signature 19, which are linked to somatic hypermutation, liver cancer and unknown factors, respectively [82]. Signature W6 was not associated with any COSMIC signatures, thus, represents a new mutational signature. A further meta-analysis performed by Letouze et al. (2017) identified a total of ten mutational signatures including COSMIC Signatures: 1, 4, 5, 6, 12, 16, 17, 22, 23, and 24 [47]. A mutational signature characterized with increased C>A transversions was a major contribution to the driver mutations found in HCC patients exposed to aflatoxin B1 [36]. A high proportion of Taiwanese HCC patients marked with aristolochic acid (AA) mutagen exposure had T>A mutations that correspond to COSMIC signature 22 [43]. The AA signature was also found to be higher in HCC patients from China and Southeast Asia while much lower in Japan, America and Europe. A prominent mutational signature was also identified after cisplatin treatment in human liver cancer cell line HepG2 [83]. Mutational signatures not only allow us to appreciate the mechanisms underlying

somatic mutations in HCC tumors, but they could relate to mutational processes in other cancer types with related aetiology.

Multi-omics analysis combine results from more than one type of data to give us a more comprehensive view of biological profiles. Boyault *et al.* (2007) conducted an unsupervised transcriptome analysis to identify six subgroups of HCC, G1-G6, where G1-G3 are associated with chromosomal instability, G5-G6 are related to β-catenin mutations while G4 is a heterogenous group [84]. The association between HCC transcriptome subclasses, G5-G6, involved in Wnt pathway activation and *CTNNB1* mutations, has been validated using WXS data in a later study [44, 84]. In addition, multi-omics analysis show that there is a correlation between gene expression profiles from RNA-seq data and allele frequencies of somatic mutations from WGS , highlighting a total of 252 genomic mutations that causes transcriptomic aberrations [33].

With the large number of available NGS-based HCC studies, there is an opportunity to integrate data across studies to provide greater statistical power and elimination of potential biases from a single cohort study. A study by Zhang et al. (2014) collected four datasets containing 99, 88, 10 and 10 HCC samples respectively to identify known and also novel mutated genes and pathways [85]. This study illustrated that larger sample sizes can identify mutations at lower frequencies in HCC than in smaller sample cohorts. As a second example of data integration, using combined liver cancer data from ICGC and TCGA to analyse the association of ancestry to HCC mutational signatures, an increase in T>C substitutions (in the ATA context) in Japanese males and an increase in T>A substitutions (in the CTG context) in US-Asian males and females were also reported [51].

Future

Mutations in the non-coding regulatory regions of the genome

Non-coding DNA makes up over 98% of the human genome and include crucial transcription factor binding sites that regulate the transcription of RNA. Non-coding RNA includes introns, 3’ and 5’ UTR located in pre-mRNAs as well as microRNAs and long non-coding RNAs (lincRNAs) [86, 87]. The functional annotation of non-coding elements from the Encyclopedia of DNA elements (ENCODE) consortium and the US NIH Roadmap Epigenomics project have provided support for the study of non-coding regions of the human DNA [88, 89]. Cancer whole genome data from TCGA have been intensively analysed to identify mutations in the non-coding regions. For example, two pan-cancer studies have shown that *TERT* promoter mutations are present in at least 6 cancer types including glioblastoma, bladder, low-grade glioma, melanoma, lung (and liver which is analysed by one of the studies) [64, 90].

TERT promoter mutations are detected in 254 of 469 cases of HCC (54%) and more frequently detected in HCV-positive and non-viral cases than HBV-positive cases [51]. A more in-depth study reveals other noncoding mutations in *NEAT1*, *MALAT1*, *WDR*74 promoter, *BCL6* promoter and *TFPI2* promoter [29]. Non-coding DNA analysis is challenging because many of the non-coding mutations are reported at lower mutation frequencies and at DNA locus with limited information of its function. We may overcome limitations in sample size and statistical power of patient datasets by analysing an increased number of liver cancer whole genomes. Hence, there is potential to better characterize non-coding regions in the future.

AAV2 viral integration events

In addition to HBV integration, recent reports of the observation of integration of the wild-type adeno-associated virus 2 (AAV2) in 11 out of 193 cases of HCC via deep sequencing [45, 91] have sparked a debate regarding the safety issues of using AAV2 as a gene delivery vector in gene therapy

[92-95]. Coincidently, the AAV2 integrations were detected in several recurrent mutation sites in HCC including the *TERT* promoter, *MLL4*, *CCNE1*, *CCNA2* and *TNFSF10* [45, 96].

An independent study by Fujimoto et al. (2016) detected AAV genome sequences in 3 liver cancer and 3 non-cancer liver cases. These 3 liver cancer cases were also infected with either HBV or HCV and the AAV2 integration sites were located at *MLL4*, *CCNE1* and an intergenic region of chromosome 5 respectively [29]. HBV integration sites were detected at *CCNA2 locus* in one patient in this study as well as an early, well-differentiated HCC patient [12]. With these observations, additional analysis are necessary to evaluate the prevalence and effects of AAV2 integration events in liver cancer and in gene therapy. The extensiveness of WGS data is therefore applicable to the detection of foreign genomic material present in the human genome that may influence the development and the treatment of liver cancer.

RNA editing

RNA editing caused by the deamination of nucleotide bases on an RNA sequence is catalysed by the nucleotide-specific deaminases. Historically, transgenic mice and rabbits expressing mRNA editing enzyme APOBEC-1 (C-to-U editing) resulted in unexpected liver dysplasia with a few of the mice developing HCC [97]. The main form of RNA editing is A-to-I editing catalysed by the adenosine deaminase acting on RNA (ADAR) (A-to-I editing) family [98].

A genome-wide study that used both WGS and RNA-seq data reported normal- and tumor-specific RNA editing sites in HCC as well as the positive correlation between editing degree ratio and gene expression ratio [35]. Results show that the increased expression of ADAR1 resulted in the over-editing of the *AZIN1* gene in HCC tumors, confirming the findings from a previous study [99]. Another genome-wide study showed that besides *AZIN1*, the *BLCAP* RNA has been over-edited (A-to-I editing) in HCC and functional analysis suggest that the over-edited *BLCAP* resulted in enhanced cell proliferation and the activation of the AKT/mTOR signal pathway [100]. Two pan-cancer studies

involving A-to-I RNA editing using data from TCGA reported no significant differences between matched normal and tumor samples, although a high Alu editing index (AEI) in HCC has been significantly associated with poor survival [101, 102].

Expanding the cancer genome database

With rapidly falling costs and newer technologies, the number of whole genomes sequenced in the next ten years is projected to increase dramatically [103]. Larger sample sizes will provide better statistical power to detect rare variants and subgroups of liver cancer, particularly in HCC. For example, a large-scale whole genome study was conducted on the Icelandic population identified missense single nucleotide polymorphism (SNP) variants in *ABCB4* to be associated with gallstone disease, liver cancer, liver cirrhosis and other liver-specific traits [104, 105]. There are currently several international collaborations to generate more cancer whole genome. The Pan-cancer Analysis of Whole Genomes (PCAWG) is an international collaboration project between ICGC and TCGA to analyse more than 2,800 whole genomes across different cancer types to identify genetic alterations, beginning with 12 tumor types profiled by TCGA although HCC was not included [106]. Additionally, the 100,000 Genomes Project by Genomics England in UK will consist of samples from 25,000 cancer patients [107].

Conclusion

In this review, we have discussed about the key findings from WGS information (Fig. 1) and future directions of HCC. WGS is a promising approach that provides genomic information for discovery-based genomic analyses in the future. Hence, it holds great potential for liver cancer research as we seek to understand more about the genetic characteristics of HCC, which is influenced by gender, ethnicity, geolocation and many risk factors. This review identified genes with somatic mutations (Table 2), many of which are involved in cancer-related pathways (Fig. 2). Many of the mutated genes

are yet to be characterized for their molecular function and roles in cancer, presenting great opportunity for future research in this direction. With improved clinical annotation and the automation of data analysis, more genomic sequences can be translated into valuable biological insights.

Declarations

Ethics approval and consent to participate

Not applicable.

Consent for publication

Not applicable.

Availability of data and material

Competing interests

The authors declare that they have no competing interests.

Funding

This work was supported by a grant from the Singapore Ministry of Health's National Medical Research Council (NMRC) (NMRC/CBRG/0095/2015) as well as some block funding from National Cancer Centre Singapore and Duke-NUS Graduate Medical School to C.G.L. The funders had no role

in study design, data collection and analysis, decision to publish, or preparation of the manuscript. The authors declare no potential conflict of interest.

Authors' contributions

CL and AS conceived the project. WH coordinated and drafted the manuscript, and synthesized comments provided by all authors. All authors contributed critically important comments. All authors read and approved the final manuscript.

Acknowledgements

Not applicable

References

1. Ferlay J SI, Ervik M, Dikshit R, Eser S, Mathers C, Rebelo M, Parkin DM, Forman D, Bray, F.: GLOBOCAN 2012 v1.0, Cancer Incidence and Mortality Worldwide: IARC CancerBase No. 11 http://globocan.iarc.fr (2013). Accessed 19th May 2018.
2. El-Serag HB and Rudolph KL. Hepatocellular carcinoma: epidemiology and molecular carcinogenesis. Gastroenterology. 2007;132 7:2557-76. doi:10.1053/j.gastro.2007.04.061.
3. El-Serag HB. Hepatocellular carcinoma. N Engl J Med. 2011;365 12:1118-27. doi:10.1056/NEJMra1001683.
4. El-Serag HB. Epidemiology of viral hepatitis and hepatocellular carcinoma. Gastroenterology. 2012;142 6:1264-73 e1. doi:10.1053/j.gastro.2011.12.061.
5. Di Bisceglie AM, Simpson LH, Lotze MT and Hoofnagle JH. Development of hepatocellular carcinoma among patients with chronic liver disease due to hepatitis C viral infection. J Clin Gastroenterol. 1994;19 3:222-6.

6. Takano S, Yokosuka O, Imazeki F, Tagawa M and Omata M. Incidence of hepatocellular carcinoma in chronic hepatitis B and C: a prospective study of 251 patients. Hepatology (Baltimore, Md). 1995;21 3:650-5.

7. Chang MH, Chen CJ, Lai MS, Hsu HM, Wu TC, Kong MS, et al. Universal hepatitis B vaccination in Taiwan and the incidence of hepatocellular carcinoma in children. Taiwan Childhood Hepatoma Study Group. N Engl J Med. 1997;336 26:1855-9. doi:10.1056/nejm199706263362602.

8. Aspinall EJ, Hawkins G, Fraser A, Hutchinson SJ and Goldberg D. Hepatitis B prevention, diagnosis, treatment and care: a review. Occup Med (Lond). 2011;61 8:531-40. doi:10.1093/occmed/kqr136.

9. Paterlini-Brechot P, Saigo K, Murakami Y, Chami M, Gozuacik D, Mugnier C, et al. Hepatitis B virus-related insertional mutagenesis occurs frequently in human liver cancers and recurrently targets human telomerase gene. Oncogene. 2003;22 25:3911-6. doi:10.1038/sj.onc.1206492.

10. Tamori A, Yamanishi Y, Kawashima S, Kanehisa M, Enomoto M, Tanaka H, et al. Alteration of gene expression in human hepatocellular carcinoma with integrated hepatitis B virus DNA. Clinical cancer research : an official journal of the American Association for Cancer Research. 2005;11 16:5821-6. doi:10.1158/1078-0432.CCR-04-2055.

11. Nagaya T, Nakamura T, Tokino T, Tsurimoto T, Imai M, Mayumi T, et al. The mode of hepatitis B virus DNA integration in chromosomes of human hepatocellular carcinoma. Genes & development. 1987;1 8:773-82.

12. Wang J, Chenivesse X, Henglein B and Brechot C. Hepatitis B virus integration in a cyclin A gene in a hepatocellular carcinoma. Nature. 1990;343 6258:555-7. doi:10.1038/343555a0.

13. Dejean A, Bougueleret L, Grzeschik KH and Tiollais P. Hepatitis B virus DNA integration in a sequence homologous to v-erb-A and steroid receptor genes in a hepatocellular carcinoma. Nature. 1986;322 6074:70-2. doi:10.1038/322070a0.

14. Satoh S, Daigo Y, Furukawa Y, Kato T, Miwa N, Nishiwaki T, et al. AXIN1 mutations in hepatocellular carcinomas, and growth suppression in cancer cells by virus-mediated transfer of AXIN1. Nat Genet. 2000;24 3:245-50. doi:10.1038/73448.

15. Murakami Y, Hayashi K, Hirohashi S and Sekiya T. Aberrations of the tumor suppressor p53 and retinoblastoma genes in human hepatocellular carcinomas. Cancer research. 1991;51 20:5520-5.

16. Goodwin S, McPherson JD and McCombie WR. Coming of age: ten years of next-generation sequencing technologies. Nat Rev Genet. 2016;17 6:333-51. doi:10.1038/nrg.2016.49.

17. Jiang Z, Jhunjhunwala S, Liu J, Haverty PM, Kennemer MI, Guan Y, et al. The effects of hepatitis B virus integration into the genomes of hepatocellular carcinoma patients. Genome research. 2012;22 4:593-601. doi:10.1101/gr.133926.111.

18. Sung WK, Zheng H, Li S, Chen R, Liu X, Li Y, et al. Genome-wide survey of recurrent HBV integration in hepatocellular carcinoma. Nat Genet. 2012;44 7:765-9. doi:10.1038/ng.2295.

19. Toh ST, Jin Y, Liu L, Wang J, Babrzadeh F, Gharizadeh B, et al. Deep sequencing of the hepatitis B virus in hepatocellular carcinoma patients reveals enriched integration events, structural alterations and sequence variations. Carcinogenesis. 2013;34 4:787-98. doi:10.1093/carcin/bgs406.

20. Tao Y, Ruan J, Yeh SH, Lu X, Wang Y, Zhai W, et al. Rapid growth of a hepatocellular carcinoma and the driving mutations revealed by cell-population genetic analysis of whole-genome data. Proceedings of the National Academy of Sciences of the United States of America. 2011;108 29:12042-7. doi:10.1073/pnas.1108715108.

21. Totoki Y, Tatsuno K, Yamamoto S, Arai Y, Hosoda F, Ishikawa S, et al. High-resolution characterization of a hepatocellular carcinoma genome. Nat Genet. 2011;43 5:464-9. doi:10.1038/ng.804.

22. Li M, Zhao H, Zhang X, Wood LD, Anders RA, Choti MA, et al. Inactivating mutations of the chromatin remodeling gene ARID2 in hepatocellular carcinoma. Nat Genet. 2011;43 9:828-9. doi:10.1038/ng.903.

23. Schulze K, Nault JC and Villanueva A. Genetic profiling of hepatocellular carcinoma using next-generation sequencing. Journal of hepatology. 2016;65 5:1031-42. doi:10.1016/j.jhep.2016.05.035.

24. Kaminuma E, Mashima J, Kodama Y, Gojobori T, Ogasawara O, Okubo K, et al. DDBJ launches a new archive database with analytical tools for next-generation sequence data. Nucleic acids research. 2010;38 Database issue:D33-8. doi:10.1093/nar/gkp847.

25. Leinonen R, Sugawara H and Shumway M. The sequence read archive. Nucleic acids research. 2011;39 Database issue:D19-21. doi:10.1093/nar/gkq1019.

26. Sneddon TP, Li P and Edmunds SC. GigaDB: announcing the GigaScience database. GigaScience. 2012;1 1:1-2. doi:10.1186/2047-217x-1-11.

27. Kan Z, Zheng H, Liu X, Li S, Barber TD, Gong Z, et al. Hepatocellular carcinoma genomic data from the Asian Cancer Research Group. GigaScience, 2012.

28. Hudson TJ, Anderson W, Artez A, Barker AD, Bell C, Bernabe RR, et al. International network of cancer genome projects. Nature. 2010;464 7291:993-8. doi:10.1038/nature08987.

29. Fujimoto A, Furuta M, Totoki Y, Tsunoda T, Kato M, Shiraishi Y, et al. Whole-genome mutational landscape and characterization of noncoding and structural mutations in liver cancer. Nat Genet. 2016;48 5:500-9. doi:10.1038/ng.3547.

30. Fujimoto A, Furuta M, Shiraishi Y, Gotoh K, Kawakami Y, Arihiro K, et al. Whole-genome mutational landscape of liver cancers displaying biliary phenotype reveals hepatitis impact and molecular diversity. Nat Commun. 2015;6:6120. doi:10.1038/ncomms7120.

31. Fujimoto A, Totoki Y, Abe T, Boroevich KA, Hosoda F, Nguyen HH, et al. Whole-genome sequencing of liver cancers identifies etiological influences on mutation patterns and recurrent mutations in chromatin regulators. Nat Genet. 2012;44 7:760-4. doi:10.1038/ng.2291.

32. Hirotsu Y, Zheng TH, Amemiya K, Mochizuki H, Guleng B and Omata M. Targeted and exome sequencing identified somatic mutations in hepatocellular carcinoma. Hepatology research : the official journal of the Japan Society of Hepatology. 2016; doi:10.1111/hepr.12663.

33. Shiraishi Y, Fujimoto A, Furuta M, Tanaka H, Chiba K, Boroevich KA, et al. Integrated analysis of whole genome and transcriptome sequencing reveals diverse transcriptomic aberrations driven by somatic genomic changes in liver cancers. PloS one. 2014;9 12:e114263. doi:10.1371/journal.pone.0114263.

34. Huang J, Deng Q, Wang Q, Li KY, Dai JH, Li N, et al. Exome sequencing of hepatitis B virus-associated hepatocellular carcinoma. Nat Genet. 2012;44 10:1117-21. doi:10.1038/ng.2391.

35. Kang L, Liu X, Gong Z, Zheng H, Wang J, Li Y, et al. Genome-wide identification of RNA editing in hepatocellular carcinoma. Genomics. 2015;105 2:76-82. doi:10.1016/j.ygeno.2014.11.005.

36. Zhang W, He H, Zang M, Wu Q, Zhao H, Lu LL, et al. Genetic Features of Aflatoxin-Associated Hepatocellular Carcinoma. Gastroenterology. 2017;153 1:249-62.e2. doi:10.1053/j.gastro.2017.03.024.

37. Fernandez-Banet J, Lee NP, Chan KT, Gao H, Liu X, Sung WK, et al. Decoding complex patterns of genomic rearrangement in hepatocellular carcinoma. Genomics. 2014;103 2-3:189-203. doi:10.1016/j.ygeno.2014.01.003.

38. Kan Z, Zheng H, Liu X, Li S, Barber TD, Gong Z, et al. Whole-genome sequencing identifies recurrent mutations in hepatocellular carcinoma. Genome research. 2013;23 9:1422-33. doi:10.1101/gr.154492.113.

39. Ahn SM, Jang SJ, Shim JH, Kim D, Hong SM, Sung CO, et al. Genomic portrait of resectable hepatocellular carcinomas: implications of RB1 and FGF19 aberrations for patient stratification. Hepatology (Baltimore, Md). 2014;60 6:1972-82. doi:10.1002/hep.27198.

40. Ouyang L, Lee J, Park CK, Mao M, Shi Y, Gong Z, et al. Whole-genome sequencing of matched primary and metastatic hepatocellular carcinomas. BMC Med Genomics. 2014;7:2. doi:10.1186/1755-8794-7-2.

41. Woo HG, Kim SS, Cho H, Kwon SM, Cho HJ, Ahn SJ, et al. Profiling of exome mutations associated with progression of HBV-related hepatocellular carcinoma. PloS one. 2014;9 12:e115152. doi:10.1371/journal.pone.0115152.

42. Lin KT, Shann YJ, Chau GY, Hsu CN and Huang CY. Identification of latent biomarkers in hepatocellular carcinoma by ultra-deep whole-transcriptome sequencing. Oncogene. 2014;33 39:4786-94. doi:10.1038/onc.2013.424.

43. Ng AWT, Poon SL, Huang MN, Lim JQ, Boot A, Yu W, et al. Aristolochic acids and their derivatives are widely implicated in liver cancers in Taiwan and throughout Asia. Science translational medicine. 2017;9 412 doi:10.1126/scitranslmed.aan6446.

44. Guichard C, Amaddeo G, Imbeaud S, Ladeiro Y, Pelletier L, Maad IB, et al. Integrated analysis of somatic mutations and focal copy-number changes identifies key genes and pathways in hepatocellular carcinoma. Nat Genet. 2012;44 6:694-8. doi:10.1038/ng.2256.

45. Nault JC, Datta S, Imbeaud S, Franconi A, Mallet M, Couchy G, et al. Recurrent AAV2-related insertional mutagenesis in human hepatocellular carcinomas. Nat Genet. 2015;47 10:1187-93. doi:10.1038/ng.3389.

46. Schulze K, Imbeaud S, Letouze E, Alexandrov LB, Calderaro J, Rebouissou S, et al. Exome sequencing of hepatocellular carcinomas identifies new mutational signatures and potential therapeutic targets. Nat Genet. 2015;47 5:505-11. doi:10.1038/ng.3252.

47. Letouzé E, Shinde J, Renault V, Couchy G, Blanc J-F, Tubacher E, et al. Mutational signatures reveal the dynamic interplay of risk factors and cellular processes during liver tumorigenesis. Nature Communications. 2017;8 1:1315. doi:10.1038/s41467-017-01358-x.

48. Cleary SP, Jeck WR, Zhao X, Chen K, Selitsky SR, Savich GL, et al. Identification of driver genes in hepatocellular carcinoma by exome sequencing. Hepatology (Baltimore, Md). 2013;58 5:1693-702. doi:10.1002/hep.26540.

49. Jhunjhunwala S, Jiang Z, Stawiski EW, Gnad F, Liu J, Mayba O, et al. Diverse modes of genomic alteration in hepatocellular carcinoma. Genome Biol. 2014;15 8:436. doi:10.1186/s13059-014-0436-9.

50. Chaudhary K, Poirion OB, Lu L, Huang S, Ching T and Garmire LX. Multi-modal meta-analysis of 1494 hepatocellular carcinoma samples reveals significant impact of consensus driver genes on phenotypes. Clinical cancer research : an official journal of the American Association for Cancer Research. 2018; doi:10.1158/1078-0432.ccr-18-0088.

51. Totoki Y, Tatsuno K, Covington KR, Ueda H, Creighton CJ, Kato M, et al. Trans-ancestry mutational landscape of hepatocellular carcinoma genomes. Nat Genet. 2014;46 12:1267-73. doi:10.1038/ng.3126.

52. Ding D, Lou X, Hua D, Yu W, Li L, Wang J, et al. Recurrent targeted genes of hepatitis B virus in the liver cancer genomes identified by a next-generation sequencing-based approach. PLoS Genet. 2012;8 12:e1003065. doi:10.1371/journal.pgen.1003065.

53. Oba A, Shimada S, Akiyama Y, Nishikawaji T, Mogushi K, Ito H, et al. ARID2 modulates DNA damage response in human hepatocellular carcinoma cells. Journal of hepatology. 2017;66 5:942-51. doi:10.1016/j.jhep.2016.12.026.

54. Gao Q, Wang K, Chen K, Liang L, Zheng Y, Zhang Y, et al. HBx protein-mediated ATOH1 downregulation suppresses ARID2 expression and promotes hepatocellular carcinoma. Cancer science. 2017;108 7:1328-37. doi:10.1111/cas.13277.

55. Chen CL, Wang Y, Pan QZ, Tang Y, Wang QJ, Pan K, et al. Bromodomain-containing protein 7 (BRD7) as a potential tumor suppressor in hepatocellular carcinoma. Oncotarget. 2016;7 13:16248-61. doi:10.18632/oncotarget.7637.

56. Zhang Q, Wei L, Yang H, Yang W, Yang Q, Zhang Z, et al. Bromodomain containing protein represses the Ras/Raf/MEK/ERK pathway to attenuate human hepatoma cell proliferation during HCV infection. Cancer letters. 2016;371 1:107-16. doi:10.1016/j.canlet.2015.11.027.

57. Chiu YH, Lee JY and Cantley LC. BRD7, a tumor suppressor, interacts with p85alpha and regulates PI3K activity. Mol Cell. 2014;54 1:193-202. doi:10.1016/j.molcel.2014.02.016.

58. Yao S, Johnson C, Hu Q, Yan L, Liu B, Ambrosone CB, et al. Differences in somatic mutation landscape of hepatocellular carcinoma in Asian American and European American populations. Oncotarget. 2016;7 26:40491-9. doi:10.18632/oncotarget.9636.

59. Viatour P, Ehmer U, Saddic LA, Dorrell C, Andersen JB, Lin C, et al. Notch signaling inhibits hepatocellular carcinoma following inactivation of the RB pathway. The Journal of experimental medicine. 2011;208 10:1963-76. doi:10.1084/jem.20110198.

60. Anwar SL, Krech T, Hasemeier B, Schipper E, Schweitzer N, Vogel A, et al. Deregulation of RB1 expression by loss of imprinting in human hepatocellular carcinoma. The Journal of pathology. 2014;233 4:392-401. doi:10.1002/path.4376.

61. Zheng S, Cherniack AD, Dewal N, Moffitt RA, Danilova L, Murray BA, et al. Comprehensive Pan-Genomic Characterization of Adrenocortical Carcinoma. Cancer cell. 2016;29 5:723-36. doi:10.1016/j.ccell.2016.04.002.

62. Nagarajan N, Bertrand D, Hillmer AM, Zang ZJ, Yao F, Jacques PE, et al. Whole-genome reconstruction and mutational signatures in gastric cancer. Genome Biol. 2012;13 12:R115. doi:10.1186/gb-2012-13-12-r115.

63. Dong H, Zhang L, Qian Z, Zhu X, Zhu G, Chen Y, et al. Identification of HBV-MLL4 Integration and Its Molecular Basis in Chinese Hepatocellular Carcinoma. PloS one. 2015;10 4:e0123175. doi:10.1371/journal.pone.0123175.

64. Fredriksson NJ, Ny L, Nilsson JA and Larsson E. Systematic analysis of noncoding somatic mutations and gene expression alterations across 14 tumor types. Nat Genet. 2014;46 12:1258-63. doi:10.1038/ng.3141.

65. Herrera-Abreu MT, Palafox M, Asghar U, Rivas MA, Cutts RJ, Garcia-Murillas I, et al. Early Adaptation and Acquired Resistance to CDK4/6 Inhibition in Estrogen Receptor-Positive Breast Cancer. Cancer research. 2016;76 8:2301-13. doi:10.1158/0008-5472.can-15-0728.

66. Scaltriti M, Eichhorn PJ, Cortes J, Prudkin L, Aura C, Jimenez J, et al. Cyclin E amplification/overexpression is a mechanism of trastuzumab resistance in HER2+ breast cancer patients. Proceedings of the National Academy of Sciences of the United States of America. 2011;108 9:3761-6. doi:10.1073/pnas.1014835108.

67. Au-Yeung G, Lang F, Azar WJ, Mitchell C, Jarman KE, Lackovic K, et al. Selective Targeting of Cyclin E1-Amplified High-Grade Serous Ovarian Cancer by Cyclin-Dependent Kinase 2 and AKT Inhibition. Clinical cancer research : an official journal of the American Association for Cancer Research. 2017;23 7:1862-74. doi:10.1158/1078-0432.ccr-16-0620.

68. Patch AM, Christie EL, Etemadmoghadam D, Garsed DW, George J, Fereday S, et al. Whole-genome characterization of chemoresistant ovarian cancer. Nature. 2015;521 7553:489-94. doi:10.1038/nature14410.

69. Ferber MJ, Montoya DP, Yu C, Aderca I, McGee A, Thorland EC, et al. Integrations of the hepatitis B virus (HBV) and human papillomavirus (HPV) into the human telomerase reverse transcriptase (hTERT) gene in liver and cervical cancers. Oncogene. 2003;22 24:3813-20. doi:10.1038/sj.onc.1206528.

70. Khoury JD, Tannir NM, Williams MD, Chen Y, Yao H, Zhang J, et al. Landscape of DNA virus associations across human malignant cancers: analysis of 3,775 cases using RNA-Seq. J Virol. 2013;87 16:8916-26. doi:10.1128/JVI.00340-13.

71. Hanahan D and Weinberg Robert A. Hallmarks of Cancer: The Next Generation. Cell. 2011;144 5:646-74. doi:http://dx.doi.org/10.1016/j.cell.2011.02.013.

72. Saigo K, Yoshida K, Ikeda R, Sakamoto Y, Murakami Y, Urashima T, et al. Integration of hepatitis B virus DNA into the myeloid/lymphoid or mixed-lineage leukemia (MLL4) gene and

rearrangements of MLL4 in human hepatocellular carcinoma. Hum Mutat. 2008;29 5:703-8. doi:10.1002/humu.20701.

73. Park MH, Kim SY, Kim YJ and Chung YH. ALS2CR7 (CDK15) attenuates TRAIL induced apoptosis by inducing phosphorylation of survivin Thr34. Biochemical and biophysical research communications. 2014;450 1:129-34. doi:10.1016/j.bbrc.2014.05.070.

74. Tanaka S, Sugimachi K, Yamashita Yi Y, Ohga T, Shirabe K, Shimada M, et al. Tie2 vascular endothelial receptor expression and function in hepatocellular carcinoma. Hepatology (Baltimore, Md). 2002;35 4:861-7. doi:10.1053/jhep.2002.32535.

75. Tanaka S, Mori M, Sakamoto Y, Makuuchi M, Sugimachi K and Wands JR. Biologic significance of angiopoietin-2 expression in human hepatocellular carcinoma. The Journal of clinical investigation. 1999;103 3:341-5. doi:10.1172/jci4891.

76. Mitsuhashi N, Shimizu H, Ohtsuka M, Wakabayashi Y, Ito H, Kimura F, et al. Angiopoietins and Tie-2 expression in angiogenesis and proliferation of human hepatocellular carcinoma. Hepatology (Baltimore, Md). 2003;37 5:1105-13. doi:10.1053/jhep.2003.50204.

77. Sugimachi K, Tanaka S, Taguchi K, Aishima S, Shimada M and Tsuneyoshi M. Angiopoietin switching regulates angiogenesis and progression of human hepatocellular carcinoma. Journal of clinical pathology. 2003;56 11:854-60.

78. Wada H, Nagano H, Yamamoto H, Yang Y, Kondo M, Ota H, et al. Expression pattern of angiogenic factors and prognosis after hepatic resection in hepatocellular carcinoma: importance of angiopoietin-2 and hypoxia-induced factor-1 alpha. Liver international : official journal of the International Association for the Study of the Liver. 2006;26 4:414-23. doi:10.1111/j.1478-3231.2006.01243.x.

79. Huang da W, Sherman BT and Lempicki RA. Systematic and integrative analysis of large gene lists using DAVID bioinformatics resources. Nat Protoc. 2009;4 1:44-57. doi:10.1038/nprot.2008.211.

80. Huang da W, Sherman BT and Lempicki RA. Bioinformatics enrichment tools: paths toward the comprehensive functional analysis of large gene lists. Nucleic acids research. 2009;37 1:1-13. doi:10.1093/nar/gkn923.

81. Forbes SA, Beare D, Boutselakis H, Bamford S, Bindal N, Tate J, et al. COSMIC: somatic cancer genetics at high-resolution. Nucleic acids research. 2017;45 D1:D777-D83. doi:10.1093/nar/gkw1121.

82. Alexandrov LB, Nik-Zainal S, Wedge DC, Aparicio SA, Behjati S, Biankin AV, et al. Signatures of mutational processes in human cancer. Nature. 2013;500 7463:415-21. doi:10.1038/nature12477.

83. Boot A, Huang MN, Ng AWT, Ho SC, Lim JQ, Kawakami Y, et al. In-depth characterization of the cisplatin mutational signature in human cell lines and in esophageal and liver tumors. Genome research. 2018;28 5:654-65. doi:10.1101/gr.230219.117.

84. Boyault S, Rickman DS, de Reynies A, Balabaud C, Rebouissou S, Jeannot E, et al. Transcriptome classification of HCC is related to gene alterations and to new therapeutic targets. Hepatology (Baltimore, Md). 2007;45 1:42-52. doi:10.1002/hep.21467.

85. Zhang Y, Qiu Z, Wei L, Tang R, Lian B, Zhao Y, et al. Integrated analysis of mutation data from various sources identifies key genes and signaling pathways in hepatocellular carcinoma. PloS one. 2014;9 7:e100854. doi:10.1371/journal.pone.0100854.

86. Ghidini M and Braconi C. Non-Coding RNAs in Primary Liver Cancer. Front Med (Lausanne). 2015;2:36. doi:10.3389/fmed.2015.00036.

87. He Y, Meng XM, Huang C, Wu BM, Zhang L, Lv XW, et al. Long noncoding RNAs: Novel insights into hepatocelluar carcinoma. Cancer letters. 2014;344 1:20-7. doi:10.1016/j.canlet.2013.10.021.

88. The-ENCODE-Project-Consortinum. An integrated encyclopedia of DNA elements in the human genome. Nature. 2012;489 7414:57-74. doi:10.1038/nature11247.

89. Bernstein BE, Stamatoyannopoulos JA, Costello JF, Ren B, Milosavljevic A, Meissner A, et al. The NIH Roadmap Epigenomics Mapping Consortium. Nat Biotechnol. 2010;28 10:1045-8. doi:10.1038/nbt1010-1045.

90. Weinhold N, Jacobsen A, Schultz N, Sander C and Lee W. Genome-wide analysis of noncoding regulatory mutations in cancer. Nat Genet. 2014;46 11:1160-5. doi:10.1038/ng.3101.

91. Nault J-C, Datta S, Imbeaud S, Franconi A and Zucman-Rossi J. Adeno-associated virus type 2 as an oncogenic virus in human hepatocellular carcinoma. Molecular & Cellular Oncology. 2016;3 2:e1095271. doi:10.1080/23723556.2015.1095271.

92. Berns KI, Byrne BJ, Flotte TR, Gao G, Hauswirth WW, Herzog RW, et al. Adeno-Associated Virus Type 2 and Hepatocellular Carcinoma? Hum Gene Ther. 2015;26 12:779-81. doi:10.1089/hum.2015.29014.kib.

93. Buning H and Schmidt M. Adeno-associated Vector Toxicity-To Be or Not to Be? Mol Ther. 2015;23 11:1673-5. doi:10.1038/mt.2015.182.

94. Gil-Farina I, Fronza R, Kaeppel C, Lopez-Franco E, Ferreira V, D'Avola D, et al. Recombinant AAV Integration Is Not Associated With Hepatic Genotoxicity in Nonhuman Primates and Patients. Mol Ther. 2016; doi:10.1038/mt.2016.52.

95. Schmidt M, Gil-Farina I and Buning H. Reply to "Wild-type AAV Insertions in Hepatocellular Carcinoma Do Not Inform Debate Over Genotoxicity Risk of Vectorized AAV". Mol Ther. 2016;24 4:661-2. doi:10.1038/mt.2016.48.
96. Nault JC, Datta S, Imbeaud S, Franconi A, Mallet M, Couchy G, et al. AAV2 and Hepatocellular Carcinoma. Hum Gene Ther. 2016;27 3:211-3. doi:10.1089/hum.2016.002.
97. Yamanaka S, Balestra ME, Ferrell LD, Fan J, Arnold KS, Taylor S, et al. Apolipoprotein B mRNA-editing protein induces hepatocellular carcinoma and dysplasia in transgenic animals. Proceedings of the National Academy of Sciences of the United States of America. 1995;92 18:8483-7.
98. Brennicke A, Marchfelder A and Binder S. RNA editing. FEMS Microbiol Rev. 1999;23 3:297-316.
99. Chen L, Li Y, Lin CH, Chan TH, Chow RK, Song Y, et al. Recoding RNA editing of AZIN1 predisposes to hepatocellular carcinoma. Nat Med. 2013;19 2:209-16. doi:10.1038/nm.3043.
100. Hu X, Wan S, Ou Y, Zhou B, Zhu J, Yi X, et al. RNA over-editing of BLCAP contributes to hepatocarcinogenesis identified by whole-genome and transcriptome sequencing. Cancer letters. 2015;357 2:510-9. doi:10.1016/j.canlet.2014.12.006.
101. Ding SL, Yang ZW, Wang J, Zhang XL, Chen XM and Lu FM. Integrative analysis of aberrant Wnt signaling in hepatitis B virus-related hepatocellular carcinoma. World J Gastroenterol. 2015;21 20:6317-28. doi:10.3748/wjg.v21.i20.6317.
102. Paz-Yaacov N, Bazak L, Buchumenski I, Porath HT, Danan-Gotthold M, Knisbacher BA, et al. Elevated RNA Editing Activity Is a Major Contributor to Transcriptomic Diversity in Tumors. Cell reports. 2015;13 2:267-76. doi:10.1016/j.celrep.2015.08.080.
103. Eisenstein M. Big data: The power of petabytes. Nature. 2015;527 7576:S2-4. doi:10.1038/527S2a.
104. Gudbjartsson DF, Helgason H, Gudjonsson SA, Zink F, Oddson A, Gylfason A, et al. Large-scale whole-genome sequencing of the Icelandic population. Nat Genet. 2015;47 5:435-44. doi:10.1038/ng.3247.
105. Lammert F and Hochrath K. A letter on ABCB4 from Iceland: On the highway to liver disease. Clin Res Hepatol Gastroenterol. 2015;39 6:655-8. doi:10.1016/j.clinre.2015.08.004.
106. Weinstein JN, Collisson EA, Mills GB, Shaw KR, Ozenberger BA, Ellrott K, et al. The Cancer Genome Atlas Pan-Cancer analysis project. Nat Genet. 2013;45 10:1113-20. doi:10.1038/ng.2764.
107. Marx V. The DNA of a nation. Nature. 2015;524 7566:503-5. doi:10.1038/524503a.

700

Abbreviations:

HCC: Hepatocellular Carcinoma; ICC: Intrahepatic Cholangiocarcinoma; cHCC/ICC: combined Hepatocellular Carcinoma/ intrahepatic cholangiocarcinoma; HBV: Hepatitis B virus positive; HCV: Hepatitis C virus positive; NBNC: Negative for HBV and HCV; FC: Fibrolamellar Carcinoma; CoCC: Cholangiocellular Carcinoma; CHTN: Cooperative Human Tissue Network

Figure 1. Summary of NGS databases in liver cancer showing its current and potential research direction

Figure 2. Reported Genes with Somatic Mutations that are significantly involved in KEGG Pathways

Table 1. List of NGS resources and their key findings from liver cancer studies

Table 2. Summary of Mutations in Liver Cancer identified through High-Throughput Genomics Data including their association with Gene Expression and Clinical Phenotype. The table indicates the nature of the mutation (SNV, indels, structural variants or copy number changes) in the coding regions. The fold-change of the gene is obtained from the TCGA microarray analysis on HCC patient samples

Table 3. Summary of HBV Viral Integration Events Occuring in HCC Patients identified through High-Throughput Genomics Data. The table indicate the genes and where the integration events occur. The fold-change of the gene is obtained from the TCGA microarray analysis on HCC patient samples

Table 1 Click here to access/download;Table;Table 1_r1.pdf

Table 1. Summary of NGS resources and their key findings from liver cancer studies

No.	Reference	Data URL	Sample Type/Total cases	Population	Viral Status	Key Findings
1	TCGA	https://dcc.icgc.org/projects/LIHC-US	54 WGS (52 HCC, 1 ICC, 1 FC)	39 White, 9 Asian, 3 African American, 3 Unknown	7 HCV, 7 HBV, 40 NBNC	TCGA-LIHC-WGS
2	TCGA	https://portal.gdc.cancer.gov/projects/TCGA-LIHC	376 WXS (366 HCC, 7 cHCC/ICC, 3 FC) + 371 RNA-seq (361 HCC, 7 cHCC/ICC, 3 FC)	187/184 White, 160/158 Asian, 17 African American, 2 American Indian/Native, 10 Unknown	49 HCV, 102 HBV, 8 HBV/HCV, 217 NBNC	TCGA-LIHC-WXS
3	Letouze et al., (2017) Nature Comm. https://doi.org/10.1038/s41467-017-01358-x	https://www.ebi.ac.uk/ega/studies/EGAS00001002408	44 WGS (35 HCC, 5 HCA, 4 FC)	40 European, 4 African	4 HCV, 5 HBV, 35 NBNC	1. Analysis of more than 300 genomes highlighted 10 mutational signatures, including ubiquitous as well as sporadic signatures.<br>2. Reconstruction of the temporal evolution in driver mutations and signatures revealed the clonal architecture in each tumour.
4	Ng et al., (2017) Sci. Transl. Med. https://doi.org/10.1126/scitranslmed.aan6446	https://www.ebi.ac.uk/ega/studies/EGAS00001002301	98 WXS (HCC)	Asian (Taiwan)	21 HCV, 56 HBV, 3 HBV/HCV, 10 NBNC, 8 N.D.	1. Distinct mutational signatures were identified in the whole exomes of HCC patients with aristolochic acid exposure.<br>2. The aristolochic acid signature also revealed known cancer driver genes, TP53 and CTNNB1, mutated 54 and 24 percent of the total HCC cases respectively.
5	Zhang et al., (2017) Gastroenterology. https://doi.org/10.1053/j.gastro.2017.03.024	unknown	49 WGS + 13 WXS (HCC)	Asian (China)	38 HBV, 9 NB, 2 N.D.	1. Aflatoxin-associated HCCs were reported to frequently contain C>A transversions, sequence motif GCN and strand bias<br>2. Frequent mutations identified in the adhesion G protein-coupled receptor B1 gene (ADGRB1) were found to be associated with increased capillary density of the tumor tissue.
6	Fujimoto et al., (2016) Nature Genetics. https://doi.org/10.1038/ng.3547	https://dcc.icgc.org/projects/LIRI-JP https://www.ebi.ac.uk/ega/studies/EGAS00001000671	300 WGS (268 HCC, 24 ICC, 8 cHCC/ICC) + 254 RNA-seq	Asian (Japan)	159 HCV, 82 HBV, 4 HBV/HCV, 55 NBNC	1. Coding and noncoding regions (including NEAT1 and MALAT1) were identified to have signifcant mutations<br>2. Structural variation analysis reveal cancer-related genes (eg. TERT and NCOR1) that lead to altered expression
7	Hirotsu et al., (2016) Hepatology Research. https://doi.org/10.1111/hepr.12663	http://trace.ddbj.nig.ac.jp/DRASearch/submission?acc=DRA003210	9 WXS (HCC)	Asian (Japan)	1 HBV, 5 HCV, 3 NBNC	1. Targeted deep sequencing analysis showed that TP53 (3/9 cases) and CTNNB1 (2/9 cases) were recurrent missense muatations in HCCs.<br>2. Functional analysis of the β-catenin H36P mutant was observed to be resistant to protein degradation and promotes HCC cell proliferation.
8	Fujimoto et al., (2015) Nature Comm. https://doi.org/10.1038/ncomms7120	https://dcc.icgc.org/projects/LIRI-JP	90 WGS (60 HCC, 7 cHCC/ICC, 22 ICC, 1 CoCC) + 69 RNA-seq	Asian (Japan)	60 HCC: 23 HBV, 29 HCV, 3 HBV/HCV, 5 NBNC	1. cHCC/ICC and CoCC showing biliary epithelial differentiation (LCB) have recurrent mutations in the TERT promoter and chromatin regulators<br>2. Hepatitis-positive HCC and cHCC/CC had a larger frequency of TERT promoter mutations and a lower frequency of KRAS and IDH1/2 mutations than hepatitis-negative LCB
9	Kang et al., (2015) Genomics. https://doi.org/10.1016/j.ygeno.2014.11.005	http://www.ebi.ac.uk/ena/data/view/ERP001196 http://gigadb.org/dataset/100034	9 WGS + RNA-seq (HCC)	Asian (Hong Kong)	HBV	1. An improved bioinformatics pipeline detects RNA-editing events in HCC tumor and matched adjacent tissues.<br>2. Varying editing degrees were significant in 13 cancer related genes from 18 editing sites and one gene with editing in the CDS region between normal and tumor tissues.
10	Schulze et al., (2015) Nature Genetics. https://doi.org/10.1038/ng.3252	https://dcc.icgc.org/projects/LICA-FR https://www.ebi.ac.uk/ega/studies/EGAS00001000217	236 WXS (HCC)	European (193 France, 9 Spain, 41 Italy)	57 HCV, 29 HBV, 4 HBV/HCV, 142 NBNC	1. Mutational signatures were significantly associated with demographic, etiological and molecular features.<br>2. Signature 23 that contained predominantly C>T mutations is consistent with the study by Totoki et al. (2011).
11	Nault et al., (2015) Nature Genetics. https://doi.org/10.1038/ng.3389	https://www.ebi.ac.uk/ega/studies/EGAS00001000217	193 WXS (HCC)	European (France)	36 HCV, 22 HBV, 135 NBNC	1. Clonal integration of the adeno-associated virus type 2 (AAV2) were identified in 11of 193 HCCs<br>2. AAV2 integrations occurred in known cancer driver genes including TERT, CCNA2, CCNE1, KMT2B and TNFSF10.
12	Dong et al., (2015) PLoS ONE. https://doi.org/10.1371/journal.pone.0123175	http://www.ncbi.nlm.nih.gov/bioproject/279878	55 RNA-seq (HCC)	Asian (China)	49 HBV, 7 NBNC	1. MLL4 was identified as the most frequent HBV integration site (8/44 cases).<br>2. Gene expression levels of the 8 MLL4-integration-positive samples were significantly higher than wild-type tumor and adjacent tissues.
13	Totoki et al., (2014) Nature Genetics. https://doi.org/10.1038/ng.3126	http://www.ncbi.nlm.nih.gov/gap/?term=phs000509 https://www.ebi.ac.uk/ega/studies/EGAS00001000389	503 WXS (488 HCC, 2 cHCC/ICC, 13 ICC)	414 Asian (Japan), 50 Caucasian, 14 US-Asian, 11 African American, 14 N.D.	212 HCV, 117 HBV, 12 HBV/HCV, 150 NBNC, 9 N.D.	1. 30 candidate driver genes, including non recurring mutated genes BRD7, MEN1, TSC2, SCRAP and NCOR1 were identified<br>2. Distinct substitution signatures were detected between the various ancestries and gender and but not associated with viral status
14	Shirashi et al., (2014) PLoS ONE. https://doi.org/10.1371/journal.pone.0114263	https://www.ebi.ac.uk/ega/datasets/EGAD00001001035	22 WGS + RNA-seq (HCC)	Asian (Japan)	HBV	1. Comparison of genomic and transcriptomic reads identified 292 genomic mutation-related splicing abberations<br>2. 23 of 33 HBV-human fusions were reported to affect TERT, FN1, MLL4 as well as concentrated around the HBx genes.
15	Fernandez-Banet et al., (2014) Genomics. https://doi.org/10.1016/j.ygeno.2014.01.003	http://www.ebi.ac.uk/ena/data/view/ERP001196 http://gigadb.org/dataset/100034	88 WGS (HCC)	Asian (Hong Kong)	81 HBV, 7 NBNC	1. 4314 somatic genomic rearrangement (GR) events were detected and annotated at the single-nucleotide resolution.<br>2. 5 HCC tumors harbored chromothripsis on chromosomal arms 1q, 8q and 5p. 13 genes, including CEBPB, MCL1 and AXIN1, were significantly affected by GR.
16	Jhunjhunwala et al., (2014) Genome Biology. https://doi.org/10.1186/s13059-014-0436-9	https://www.ebi.ac.uk/ega/studies/EGAS00001000824	12 WGS + RNA-seq (HCC)	Samples obtained from commerical sources	11 HBV, 1 NBNC	1. Recurrent mutations in TP53, AXIN1 and CTNNB1 were detected as well as a rare find in LAMA2 (6/42 cases) and IDH1 (2/42 cases).<br>2. The activation of TERT was either due to viral integrations in its promoter or its translocation to another chromosomal region.

17	Ahn et al., (2014) Hepatology. https://doi.org/10.1002/hep.27198	unknown	231 WXS (HCC)	Asian (Korea)	167 HBV, 22 HCV, 42 NBNC	1. Nine significantly mutated genes and cellular pathways such as p53, Wnt, PIK3/Ras, cell cycle and chromatin remodeling account for ~80% of the mutations identified in the 231 tumors.<br><br>2. Genetic abberations in the cell cycle pathway genes (RB1, MYC, CCND1, RBL2) were associated with cancer-specific and recurrence-free survival.
18	Woo et al., (2014) PLoS ONE. https://doi.org/10.1371/journal.pone.0115152	unknown	12 WXS (HCC)	Asian (Korea)	HBV	1. Tumor specific genes such as CTNNB1, TTN, SETD2 and ALK have been identified.<br><br>2. The T>A transversions were present significantly and exclusively in tumor-specific variants.
19	Ouyang et al. (2014) BMC Medical Genomics. https://doi.org/10.1186/1755-8794-7-2	https://trace.ddbj.nig.ac.jp/DRASearch/submission?acc=SRA076160	4 WGS (HCC)	Asian (Korea)	HBV	1. Analysis of the mutational spectrum showed that C>T transition rates within the coding regions were the highest.<br><br>2. Altered pathways in primary tumor were Wnt, JAK-STAT, cell cycle and focal adhersion pathways while tight junction, focal adhesion and ErbB/MAPK pathways were affected in the metastases.
20	Kan et al., (2013) Genome Research. https://doi.org/10.1101/gr.154492.113	http://www.ebi.ac.uk/ena/data/view/ERP001196<br>http://gigadb.org/dataset/100034	88 WGS (HCC)	Asian (Hong Kong)	81 HBV, 7 NBNC	1. The study reveals recurrent mutations in TP53, CTNNB1 and AXIN1, two genes (JAK1, LRPB1) commonly mutated in other cancers as well as six genes previously not reported.<br><br>2. Pathways affected include Wnt, cytokine-induced JAK/STAT, G1/S cell cycle and apoptosis.
21	Toh et al., (2013) Carcinogenesis. https://doi.org/10.1093/carcin/bgs406	unknown	48 FLX-Seq (HCC)	Asian (Singapore)	48 HBV	1. Preferential integration of HBV into the TERT promoter (6/97 cases).<br><br>2. The 3'-end of the HBV X protein is the preferred HBV genomic region detected in the integration events.
22	Cleary et al., (2013) Hepatology. https://doi.org/10.1002/hep.26540	http://www.ncbi.nlm.nih.gov/projects/gap/cgi-bin/study.cgi?study_id=phs000627.v1.p1	87 WXS (HCC)	Samples obtained from Canada, North Carolina and CHTN	19 HCV, 38 HBV, 30 NBNC	1. 13 significantly mutated genes identified include CTNNB1, TP53, CPA2, IGSF3 and KEAP1 as well as four significantly mutated gene families.<br><br>2. Further validation of the MLL gene family revealed MLL4 (6/13 missense mutations) to be a potential driver gene of HCC.
23	Lin et al., (2013) Oncogene. https://doi.org/10.1038/onc.2013.424	https://trace.ddbj.nig.ac.jp/DRASearch/study?acc=SRP007560	55 RNA-seq (HCC)	Asian (Taiwan)	20 HBV, 18 HCV, 17 NBNC	1. Putative mRNA sequences filtered via Cufflinks de novo assembly identified, DUNQU1, a 101-amino-acid peptide encoded by 3 exons.<br><br>2. Analysis of alternative splicing in transcripts revealed three cancer-related events in FGFR2, EXOC7 and ADAM15.
24	Fujimoto et al., (2012) Nature Genetics. https://doi.org/10.1038/ng.2291	https://dcc.icgc.org/projects/LINC-JP	27 WGS (HCC)	Asian (Japan)	11 HBV, 14 HCV, 2 NBNC	1. TP53 and CTNNB1, as well as ATM, ARID1A, ERRF11, WWP1 mutations were detected in the tumors.<br><br>2. Gene-set enrichment analysis identified several genes associated with chromatin regulation.
25	Sung et al., (2012) Nature Genetics. https://doi.org/10.1038/ng.2295	http://www.ebi.ac.uk/ena/data/view/ERP001196<br>http://gigadb.org/dataset/100034	88 WGS (HCC)	Asian (Hong Kong)	81 HBV, 7 NBNC	1. 179 of the 399 HBV integration breakpoints were identified in known coding genes.<br><br>2. HBV integrations led to increased gene expression of TERT, MLL4 and CCNE1.
26	Guichard et al., (2012) Nature Genetics. https://doi.org/10.1038/ng.2256	https://www.ebi.ac.uk/ega/studies/EGAS00001000217	24 WXS (HCC)	European (France)	4 HCV, 1 HBV, 19 NBNC	1. 850 mutations corresponded to single-nucleotide variants, particularly C>T changes that occur more frequently in non-cirrhotic liver HCC tumors.<br><br>2. Major pathways with frequently altered genes identified include Wnt and p53 pathways as well as four recurrent mutations (ARID1A, RPS6KA3, NFE2L2 & IRF2) previously not reported.
27	Jiang et al., (2012) Genome Research. https://doi.org/10.1101/gr.133926.111	http://www.ncbi.nlm.nih.gov/projects/gap/cgi-bin/study.cgi?study_id=phs000384.v1.p1	4 WGS + RNA-seq (HCC)	Samples obtained from commerical sources	3 HBV, 1 NBNC	1. RNA-seq expression analysis revealed the impact of HBV integrations on adjacent transcription activation of MLL4 and ANGPT1 in different patients.<br><br>2. There is a strong bias of viral-fusion transcripts containing HBV genome sequences near its direct repeat 1 (DR1) region.
28	Huang et al., (2012) Nature Genetics. https://doi.org/10.1038/ng.2391	http://www.ncbi.nlm.nih.gov/bioproject/PRJNA167270	10 WXS (HCC)	Asian (China)	8 HBV, 2 NBNC	1. The comparison between matched samples of HBV-associated HCC individuals (primary tumor vs. portal vein tumor thromboses) reveals 65 mutations including TP53 and ARID1A.<br><br>2. ARID1A mutations were also identified in four HCC cell lines with high metastatic potential.
29	Totoki et al., (2011) Nature Genetics. https://doi.org/10.1038/ng.804	https://dcc.icgc.org/projects/LINC-JP	1 WGS (HCC)	Asian (Japan)	HCV	1. The study identifies somatic substitutions patterns predominantly from T>C and C>T transitions.<br><br>2. Somatic alterations include well known tumor suppressors TP53 and AXIN1 as well as five other genes found commonly mutated in other cancers.
30	Li et al., (2011) Nature Genetics. https://doi.org/10.1038/ng.903	unknown	139 WXS (HCC)	US (44 White, 15 Black, 9 Asian, 1 Hispanic, 1 Arabic, 8 Unknown), China (61 Asian)	43 HCV, 50 HBV, 2 HBV/HCV, 44 NBNC	1. Somatic mutations were found in five genes (CTNNB1, TP53, ARID2, DMXL1 and NLRP1).<br><br>2. 6 out of 9 of the samples containing ARID2 mutations also contained CTNNB1 mutations but none of them contained TP53 mutations.
			Total		Total	
			582 WGS; 1211 WXS; 778 RNA-seq; 48 FLX-seq		43.71% HBV; 21.13% HCV; 34.48% NBNC	

Table 2. Summary of Mutations in Liver Cancer identified through High-Throughput Genomics Data including their association with Gene Expression and Clinical Phenotype

No.	Gene	Point mutations	Indels	Structural variants	Copy number alterations (↓|↑)	Gene expression fold-change in TCGA-HCC dataset (T/N)	Median Exp	Histologic grade G1	Histologic grade G2	Histologic grade G3	Histologic grade G4	Histologic grade G_un	Total Cases	Survival Cases deceased	Survival Median survival days	References
1	*ALB*	•	•	•	↓	0.96	HIGH<br>LOW	5<br>5	21<br>20	11<br>16	1<br>2	48<br>42	86<br>85	9<br>6	268<br>228	Letouze et al., (2017) Nature Comm., 8, 1315.<br>Ng et al., (2017) Sci. Transl. Med., 9, eaan6446.<br>Fujimoto et al., (2016) Nature Genetics, 48, 500-9.<br>Schulze et al., (2015) Nature Genetics, 47, 505-11.<br>Ahn et al., (2014) Hepatology, 60, 1972-82.<br>Fernandez-Banet et al., (2014) Genomics, 103, 189-203.<br>Fujimoto et al., (2012) Nature Genetics, 44, 760-4.<br>Guichard et al., (2012) Nature Genetics, 44, 694-698.
2	*ARID2*	•	•	•	↓	0.97	HIGH<br>LOW	4<br>6	24<br>17	15<br>12	1<br>2	42<br>48	86<br>85	6<br>9	91<br>410	Letouze et al., (2017) Nature Comm., 8, 1315.<br>Ng et al., (2017) Sci. Transl. Med., 9, eaan6446.<br>Fujimoto et al., (2016) Nature Genetics, 48, 500-9.<br>Schulze et al., (2015) Nature Genetics, 47, 505-11.<br>Shirashi et al., (2014) PLoS ONE, 9, e114263.<br>Guichard et al., (2012) Nature Genetics, 44, 694-698.<br>Li et al., (2011) Nature Genetics, 43, 828-9.
3	*RB1*	•	•	•	↓	1.04	HIGH<br>LOW	3<br>7	23<br>18	18<br>9	1<br>2	41<br>49	86<br>85	8<br>7	91<br>482.5	Letouze et al., (2017) Nature Comm., 8, 1315.<br>Ng et al., (2017) Sci. Transl. Med., 9, eaan6446.<br>Fujimoto et al., (2016) Nature Genetics, 48, 500-9.<br>Schulze et al., (2015) Nature Genetics, 47, 505-11.<br>Ahn et al., (2014) Hepatology, 60, 1972-82.<br>Kan et al., (2013) Genome Research, 23, 1422-33.
4	*RPL22*	•	•	•	↓	1.01	HIGH<br>LOW	6<br>4	23<br>18	19<br>8	1<br>2	37<br>53	86<br>85	7<br>8	171<br>410	Letouze et al., (2017) Nature Comm., 8, 1315.<br>Fujimoto et al., (2016) Nature Genetics, 48, 500-9.<br>Schulze et al., (2015) Nature Genetics, 47, 505-11.
5	*BRD7*	•	•	•	↓	1.03	HIGH<br>LOW	7<br>3	24<br>17	17<br>10	1<br>2	37<br>53	86<br>85	9<br>6	228<br>290.5	Ng et al., (2017) Sci. Transl. Med., 9, eaan6446.<br>Fujimoto et al., (2016) Nature Genetics, 48, 500-9.<br>Shirashi et al., (2014) PLoS ONE, 9, e114263.
6	*RPS6KA3*	•	•	•		1.03	HIGH<br>LOW	3<br>7	17<br>24	17<br>10	-<br>3	49<br>41	86<br>85	7<br>8	228<br>290.5	Letouze et al., (2017) Nature Comm., 8, 1315.<br>Ng et al., (2017) Sci. Transl. Med., 9, eaan6446.<br>Fujimoto et al., (2016) Nature Genetics, 48, 500-9.<br>Schulze et al., (2015) Nature Genetics, 47, 505-11.<br>Ahn et al., (2014) Hepatology, 60, 1972-82.<br>Shirashi et al., (2014) PLoS ONE, 9, e114263.<br>Guichard et al., (2012) Nature Genetics, 44, 694-698.
7	*ARID1A*	•	•	•		1.05	HIGH<br>LOW	5<br>5	25<br>16	14<br>13	-<br>3	42<br>48	86<br>85	8<br>7	228<br>290.5	Letouze et al., (2017) Nature Comm., 8, 1315.<br>Fujimoto et al., (2016) Nature Genetics, 48, 500-9.<br>Schulze et al., (2015) Nature Genetics, 47, 505-11.<br>Fujimoto et al., (2012) Nature Genetics, 44, 760-4.<br>Guichard et al., (2012) Nature Genetics, 44, 694-698.<br>Huang et al., (2012) Nature Genetics, 44, 1117-21.
8	*CDKN2A*	•		•	↓	1.08	HIGH<br>LOW	5<br>5	21<br>20	12<br>15	1<br>2	47<br>43	86<br>85	7<br>8	323<br>268	Letouze et al., (2017) Nature Comm., 8, 1315.<br>Fujimoto et al., (2016) Nature Genetics, 48, 500-9.<br>Schulze et al., (2015) Nature Genetics, 47, 505-11.<br>Guichard et al., (2012) Nature Genetics, 44, 694-698.
9	*PTEN*	•	•	•		1.02	HIGH<br>LOW	3<br>7	20<br>21	16<br>11	-<br>3	47<br>43	86<br>85	10<br>5	91<br>410	Ng et al., (2017) Sci. Transl. Med., 9, eaan6446.<br>Fujimoto et al., (2016) Nature Genetics, 48, 500-9.<br>Schulze et al., (2015) Nature Genetics, 47, 505-11.<br>Shirashi et al., (2014) PLoS ONE, 9, e114263.
10	*ACVR2A*	•	•	•		1.02	HIGH<br>LOW	5<br>5	21<br>20	15<br>12	1<br>2	44<br>46	86<br>85	8<br>7	131<br>387.5	Letouze et al., (2017) Nature Comm., 8, 1315.<br>Ng et al., (2017) Sci. Transl. Med., 9, eaan6446.<br>Fujimoto et al., (2016) Nature Genetics, 48, 500-9.<br>Schulze et al., (2015) Nature Genetics, 47, 505-11.
11	*LRP1B*	•		•	↓	1.18	HIGH<br>LOW	8<br>2	23<br>18	12<br>15	-<br>3	43<br>47	86<br>85	10<br>5	483<br>101	Fujimoto et al., (2016) Nature Genetics, 48, 500-9.<br>Shirashi et al., (2014) PLoS ONE, 9, e114263.<br>Kan et al., (2013) Genome Research, 23, 1422-33.
12	*HNF4A*	•	•	•		1.09	HIGH<br>LOW	4<br>6	17<br>24	19<br>8	1<br>2	45<br>45	86<br>85	5<br>10	91<br>482.5	Fujimoto et al., (2016) Nature Genetics, 48, 500-9.<br>Shirashi et al., (2014) PLoS ONE, 9, e114263.
13	*NEAT1*	•	•	•		1.05	HIGH<br>LOW	2<br>8	19<br>22	14<br>13	1<br>2	50<br>40	86<br>85	5<br>10	91<br>410	Fujimoto et al., (2016) Nature Genetics, 48, 500-9.<br>Totoki et al., (2014) Nature Genetics, 46, 1267-73.
14	*CPS1*	•	•	•		1.01	HIGH<br>LOW	4<br>6	20<br>21	10<br>17	1<br>2	51<br>39	86<br>85	8<br>7	131<br>387.5	Fujimoto et al., (2016) Nature Genetics, 48, 500-9.

15	*TP53*	•	•			0.97	HIGH<br>LOW	2<br>8	21<br>20	17<br>10	2<br>1	44<br>46	86<br>85	6<br>9	108.5<br>460	Letouze et al., (2017) Nature Comm., 8, 1315.<br>Ng et al., (2017) Sci. Transl. Med., 9, eaan6446.<br>Fujimoto et al., (2016) Nature Genetics, 48, 500-9.<br>Schulze et al., (2015) Nature Genetics, 47, 505-11.<br>Ahn et al., (2014) Hepatology, 60, 1972-82.<br>Jhunjhunwala et al., (2014) Genome Biology, 15, 436.<br>Shirashi et al., (2014) PLoS ONE, 9, e114263.<br>Cleary et al., (2013) Hepatology, 58, 1693-702.<br>Kan et al., (2013) Genome Research, 23, 1422-33.<br>Fujimoto et al., (2012) Nature Genetics, 44, 760-4.<br>Huang et al., (2012) Nature Genetics, 44, 1117-21.<br>Li et al., (2011) Nature Genetics, 43, 828-9.
16	*CTNNB1*	•	•			1.11	HIGH<br>LOW	6<br>4	23<br>18	14<br>13	1<br>2	42<br>48	86<br>85	7<br>8	91<br>387.5	Letouze et al., (2017) Nature Comm., 8, 1315.<br>Ng et al., (2017) Sci. Transl. Med., 9, eaan6446.<br>Fujimoto et al., (2016) Nature Genetics, 48, 500-9.<br>Schulze et al., (2015) Nature Genetics, 47, 505-11.<br>Ahn et al., (2014) Hepatology, 60, 1972-82.<br>Jhunjhunwala et al., (2014) Genome Biology, 15, 436.<br>Cleary et al., (2013) Hepatology, 58, 1693-702.<br>Kan et al., (2013) Genome Research, 23, 1422-33.<br>Fujimoto et al., (2012) Nature Genetics, 44, 760-4.<br>Guichard et al., (2012) Nature Genetics, 44, 694-698.<br>Li et al., (2011) Nature Genetics, 43, 828-9.
17	*AXIN1*	•	•			0.96	HIGH<br>LOW	6<br>4	22<br>19	13<br>14	1<br>2	44<br>46	86<br>85	6<br>9	228<br>290.5	Letouze et al., (2017) Nature Comm., 8, 1315.<br>Ng et al., (2017) Sci. Transl. Med., 9, eaan6446.<br>Fujimoto et al., (2016) Nature Genetics, 48, 500-9.<br>Schulze et al., (2015) Nature Genetics, 47, 505-11.<br>Ahn et al., (2014) Hepatology, 60, 1972-82.<br>Fernandez-Banet et al., (2014) Genomics, 103, 189-203.<br>Jhunjhunwala et al., (2014) Genome Biology, 15, 436.<br>Kan et al., (2013) Genome Research, 23, 1422-33.<br>Guichard et al., (2012) Nature Genetics, 44, 694-698.
18	*APOB*	•	•			0.98	HIGH<br>LOW	4<br>6	19<br>22	12<br>15	1<br>2	50<br>40	86<br>85	5<br>10	892.5<br>268	Fujimoto et al., (2016) Nature Genetics, 48, 500-9.<br>Schulze et al., (2015) Nature Genetics, 47, 505-11.<br>Shirashi et al., (2014) PLoS ONE, 9, e114263.<br>Kan et al., (2013) Genome Research, 23, 1422-33.<br>Guichard et al., (2012) Nature Genetics, 44, 694-698.
19	*BAP1*	•	•			1.12	HIGH<br>LOW	5<br>5	22<br>19	15<br>12	1<br>2	43<br>47	86<br>85	7<br>8	91<br>410	Fujimoto et al., (2016) Nature Genetics, 48, 500-9.<br>Jhunjhunwala et al., (2014) Genome Biology, 15, 436.
20	*TERT*	•		•		0.97	HIGH<br>LOW	5<br>5	24<br>17	16<br>11	1<br>2	40<br>50	86<br>85	7<br>8	365<br>171	Fujimoto et al., (2016) Nature Genetics, 48, 500-9.
21	*CDKN1A*	•			↓	0.99	HIGH<br>LOW	3<br>7	23<br>18	14<br>13	2<br>1	44<br>46	86<br>85	8<br>7	410<br>46	Letouze et al., (2017) Nature Comm., 8, 1315.<br>Schulze et al., (2015) Nature Genetics, 47, 505-11.
22	*ANKRD30BL*		•	•		1.50	HIGH<br>LOW	-<br>-	-<br>-	-<br>-	-<br>-	-<br>-	-<br>-	-<br>-	-<br>-	Totoki et al., (2014) Nature Genetics, 46, 1267-73.
23	*ARID1B*	•	•			0.99	HIGH<br>LOW	5<br>5	26<br>15	13<br>14	-<br>3	42<br>48	86<br>85	9<br>6	228<br>290.5	Fujimoto et al., (2012) Nature Genetics, 44, 760-4.
24	*ASH1L*	•		•		1.01	HIGH<br>LOW	4<br>6	20<br>21	14<br>13	1<br>2	47<br>43	86<br>85	7<br>8	68.5<br>482.5	Fujimoto et al., (2016) Nature Genetics, 48, 500-9.
25	*COL6A6*	•	•			0.90	HIGH<br>LOW	7<br>3	21<br>20	14<br>13	1<br>2	43<br>47	86<br>85	9<br>6	555<br>91	Shirashi et al., (2014) PLoS ONE, 9, e114263.
26	*ERRFI1*	•	•			1.00	HIGH<br>LOW	3<br>7	26<br>15	10<br>17	-<br>3	47<br>43	86<br>85	9<br>6	365<br>171	Fujimoto et al., (2012) Nature Genetics, 44, 760-4.
27	*MLL*	•	•			-	HIGH<br>LOW	-<br>-	-<br>-	-<br>-	-<br>-	-<br>-	-<br>-	-<br>-	-<br>-	Fujimoto et al., (2012) Nature Genetics, 44, 760-4.
28	*MLL3*	•	•			-	HIGH<br>LOW	-<br>-	-<br>-	-<br>-	-<br>-	-<br>-	-<br>-	-<br>-	-<br>-	Fujimoto et al., (2012) Nature Genetics, 44, 760-4.
29	*MUC17*	•	•			1.05	HIGH<br>LOW	-<br>-	-<br>-	-<br>-	-<br>-	-<br>-	-<br>-	-<br>-	-<br>-	Fujimoto et al., (2016) Nature Genetics, 48, 500-9.

30	*SETDB1*	•	•			1.11	HIGH<br>LOW	4<br>6	16<br>25	16<br>11	1<br>2	49<br>41	86<br>85	4<br>11	91<br>410	Fujimoto et al., (2016) Nature Genetics, 48, 500-9.
31	*TBL1XR1*	•		•		1.00	HIGH<br>LOW	5<br>5	20<br>21	17<br>10	-<br>3	44<br>46	86<br>85	7<br>8	365<br>171	Fujimoto et al., (2016) Nature Genetics, 48, 500-9.
32	*MTAP*			•	↓	1.06	HIGH<br>LOW	5<br>5	25<br>16	14<br>13	1<br>2	41<br>49	86<br>85	7<br>8	38.5<br>387.5	Fujimoto et al., (2016) Nature Genetics, 48, 500-9.
33	*PER3*	•			↓	1.09	HIGH<br>LOW	6<br>4	22<br>19	13<br>14	2<br>1	43<br>47	86<br>85	8<br>7	171<br>410	Fujimoto et al., (2016) Nature Genetics, 48, 500-9.
34	*KEAP1*	•				1.03	HIGH<br>LOW	5<br>5	22<br>19	16<br>11	2<br>1	41<br>49	86<br>85	10<br>5	555<br>91	Letouze et al., (2017) Nature Comm., 8, 1315.Schulze et al., (2015) Nature Genetics, 47, 505-11.<br>Cleary et al., (2013) Hepatology, 58, 1693-702.
35	*NFE2L2*	•				1.05	HIGH<br>LOW	5<br>5	22<br>19	14<br>13	1<br>2	44<br>46	86<br>85	6<br>9	91<br>387.5	Letouze et al., (2017) Nature Comm., 8, 1315.Schulze et al., (2015) Nature Genetics, 47, 505-11.<br>Guichard et al., (2012) Nature Genetics, 44, 694-698.
36	*FLJ41941*	•				-	HIGH<br>LOW	-<br>-	-<br>-	-<br>-	-<br>-	-<br>-	-<br>-	-<br>-	-<br>-	Fujimoto et al., (2016) Nature Genetics, 48, 500-9.<br>Totoki et al., (2014) Nature Genetics, 46, 1267-73.
37	*JAK1*	•				0.98	HIGH<br>LOW	6<br>4	26<br>15	12<br>15	1<br>2	41<br>49	86<br>85	7<br>8	91<br>410	Jhunjhunwala et al., (2014) Genome Biology, 15, 436.<br>Kan et al., (2013) Genome Research, 23, 1422-33.
38	*LINC00665*	•				0.96	HIGH<br>LOW	8<br>2	21<br>20	16<br>11	-<br>3	41<br>49	86<br>85	8<br>7	365<br>171	Fujimoto et al., (2016) Nature Genetics, 48, 500-9.<br>Totoki et al., (2014) Nature Genetics, 46, 1267-73.
39	*MALAT1*	•				0.93	HIGH<br>LOW	3<br>7	21<br>20	13<br>14	2<br>1	47<br>43	86<br>85	5<br>10	91<br>555	Fujimoto et al., (2016) Nature Genetics, 48, 500-9.<br>Totoki et al., (2014) Nature Genetics, 46, 1267-73.
40	*MED16*	•				1.05	HIGH<br>LOW	5<br>5	21<br>20	17<br>10	2<br>1	41<br>49	86<br>85	8<br>7	482.5<br>131	Fujimoto et al., (2016) Nature Genetics, 48, 500-9.<br>Totoki et al., (2014) Nature Genetics, 46, 1267-73.
41	*RNA5-8SP2*	•				1.32	HIGH<br>LOW	-<br>-	-<br>-	-<br>-	-<br>-	-<br>-	-<br>-	-<br>-	-<br>-	Fujimoto et al., (2016) Nature Genetics, 48, 500-9.<br>Totoki et al., (2014) Nature Genetics, 46, 1267-73.
42	*RNU2-2P*	•				-	HIGH<br>LOW	-<br>-	-<br>-	-<br>-	-<br>-	-<br>-	-<br>-	-<br>-	-<br>-	Fujimoto et al., (2016) Nature Genetics, 48, 500-9.<br>Shirashi et al., (2014) PLoS ONE, 9, e114263.
43	*RP4-704D21.2*	•				-	HIGH<br>LOW	-<br>-	-<br>-	-<br>-	-<br>-	-<br>-	-<br>-	-<br>-	-<br>-	Fujimoto et al., (2016) Nature Genetics, 48, 500-9.<br>Fujimoto et al., (2012) Nature Genetics, 44, 760-4.
44	*WDR74*	•				1.04	HIGH<br>LOW	4<br>6	18<br>23	17<br>10	-<br>3	47<br>43	86<br>85	7<br>8	555<br>171	Fujimoto et al., (2016) Nature Genetics, 48, 500-9.<br>Kan et al., (2013) Genome Research, 23, 1422-33.
45	*MED1*			•		-	HIGH<br>LOW	-<br>-	-<br>-	-<br>-	-<br>-	-<br>-	-<br>-	-<br>-	-<br>-	Totoki et al., (2014) Nature Genetics, 46, 1267-73.
46	*AC020926.1*			•		-	HIGH<br>LOW	-<br>-	-<br>-	-<br>-	-<br>-	-<br>-	-<br>-	-<br>-	-<br>-	Totoki et al., (2014) Nature Genetics, 46, 1267-73.
47	*AF14691.4*			•		-	HIGH<br>LOW	-<br>-	-<br>-	-<br>-	-<br>-	-<br>-	-<br>-	-<br>-	-<br>-	Totoki et al., (2014) Nature Genetics, 46, 1267-73.

48	*ANGPT1*	•				1.02	HIGH<br>LOW	7<br>3	27<br>14	16<br>11	-<br>3	36<br>54	86<br>85	7<br>8	91<br>365	Jhunjhunwala et al., (2014) Genome Biology, 15, 436.
49	*ANKRD36B P2*			•		0.95	HIGH<br>LOW	5<br>5	23<br>18	12<br>15	-<br>3	46<br>44	86<br>85	5<br>10	555<br>171	Totoki et al., (2014) Nature Genetics, 46, 1267-73.
50	*APC*	•				0.99	HIGH<br>LOW	5<br>5	20<br>21	12<br>15	1<br>2	48<br>42	86<br>85	5<br>10	68.5<br>482.5	Guichard et al., (2012) Nature Genetics, 44, 694-698.
51	*ATAD3B*	•				1.08	HIGH<br>LOW	5<br>5	23<br>18	12<br>15	2<br>1	44<br>46	86<br>85	9<br>6	171<br>365	Cleary et al., (2013) Hepatology, 58, 1693-702.
52	*BRD9*	•				1.10	HIGH<br>LOW	4<br>6	22<br>19	17<br>10	1<br>2	42<br>48	86<br>85	6<br>9	61<br>387.5	Cleary et al., (2013) Hepatology, 58, 1693-702.
53	*BRE*	•				0.92	HIGH<br>LOW	5<br>5	20<br>21	12<br>15	-<br>3	49<br>41	86<br>85	8<br>7	555<br>171	Shirashi et al., (2014) PLoS ONE, 9, e114263.
54	*CCND1*			•	↑	1.00	HIGH<br>LOW	4<br>6	22<br>19	15<br>12	2<br>1	43<br>47	86<br>85	5<br>10	410<br>171	Fujimoto et al., (2016) Nature Genetics, 48, 500-9.
55	*CCNE1*			•		0.97	HIGH<br>LOW	5<br>5	23<br>18	13<br>14	2<br>1	43<br>47	86<br>85	7<br>8	482.5<br>131	Jhunjhunwala et al., (2014) Genome Biology, 15, 436.
56	*CDKN1B*	•				1.05	HIGH<br>LOW	5<br>5	26<br>15	13<br>14	-<br>3	42<br>48	86<br>85	6<br>9	46<br>410	Ahn et al., (2014) Hepatology, 60, 1972-82.
57	*CEBPB*			•		0.99	HIGH<br>LOW	3<br>7	21<br>20	13<br>14	2<br>1	47<br>43	86<br>85	10<br>5	131<br>505.5	Fernandez-Banet et al., (2014) Genomics, 103, 189-203.
58	*COL11A1*	•				-	HIGH<br>LOW	5<br>5	29<br>12	13<br>14	-<br>3	39<br>51	86<br>85	7<br>8	91<br>365	Kan et al., (2013) Genome Research, 23, 1422-33.
59	*COL6A5*	•				0.90	HIGH<br>LOW	-<br>-	-<br>-	-<br>-	-<br>-	-<br>-	-<br>-	-<br>-	-<br>-	Shirashi et al., (2014) PLoS ONE, 9, e114263.
60	*CPA2*	•				0.15	HIGH<br>LOW	-<br>-	-<br>-	-<br>-	-<br>-	-<br>-	-<br>-	-<br>-	-<br>-	Cleary et al., (2013) Hepatology, 58, 1693-702.
61	*EPS15*	•				0.99	HIGH<br>LOW	4<br>6	26<br>15	11<br>16	1<br>2	44<br>46	86<br>85	8<br>7	228<br>290.5	Kan et al., (2013) Genome Research, 23, 1422-33.
62	*FAM5C*	•				-	HIGH<br>LOW	-<br>-	-<br>-	-<br>-	-<br>-	-<br>-	-<br>-	-<br>-	-<br>-	Kan et al., (2013) Genome Research, 23, 1422-33.
63	*GJA1*	•				1.23	HIGH<br>LOW	5<br>5	22<br>19	14<br>13	1<br>2	44<br>46	86<br>85	8<br>7	131<br>505.5	Cleary et al., (2013) Hepatology, 58, 1693-702.
64	*GXYLT1*	•				1.00	HIGH<br>LOW	6<br>4	21<br>20	17<br>10	1<br>2	41<br>49	86<br>85	6<br>9	91<br>365	Fujimoto et al., (2012) Nature Genetics, 44, 760-4.

65	*IRF2*	•				1.08	HIGH<br>LOW	5<br>5	21<br>20	16<br>11	1<br>2	43<br>47	86<br>85	7<br>8	68.5<br>387.5	Guichard et al., (2012) Nature Genetics, 44, 694-698.
66	*KRAS*	•				0.99	HIGH<br>LOW	2<br>8	21<br>20	16<br>11	1<br>2	46<br>44	86<br>85	6<br>9	46<br>410	Guichard et al., (2012) Nature Genetics, 44, 694-698.
67	*KRTAP5-11*	•				0.57	HIGH<br>LOW	-<br>-	-<br>-	-<br>-	-<br>-	-<br>-	-<br>-	-<br>-	-<br>-	Fujimoto et al., (2016) Nature Genetics, 48, 500-9.
68	*MACROD2*			•		0.98	HIGH<br>LOW	5<br>5	26<br>15	13<br>14	-<br>3	42<br>48	86<br>85	8<br>7	365<br>171	Fujimoto et al., (2016) Nature Genetics, 48, 500-9.
69	*MERTK*			•		1.13	HIGH<br>LOW	5<br>5	18<br>23	15<br>12	1<br>2	47<br>43	86<br>85	4<br>11	61<br>387.5	Fernandez-Banet et al., (2014) Genomics, 103, 189-203.
70	*OTOP1*	•				1.62	HIGH<br>LOW	-<br>-	-<br>-	-<br>-	-<br>-	-<br>-	-<br>-	-<br>-	-<br>-	Fujimoto et al., (2012) Nature Genetics, 44, 760-4.
71	*PIK3CA*	•				1.04	HIGH<br>LOW	6<br>4	25<br>16	15<br>12	-<br>3	40<br>50	86<br>85	8<br>7	228<br>290.5	Guichard et al., (2012) Nature Genetics, 44, 694-698.
72	*SLC10A1*	•				0.93	HIGH<br>LOW	3<br>7	21<br>20	13<br>14	1<br>2	48<br>42	86<br>85	7<br>8	460<br>108.5	Kan et al., (2013) Genome Research, 23, 1422-33.
73	*TAF1L*	•				0.92	HIGH<br>LOW	-<br>-	-<br>-	-<br>-	-<br>-	-<br>-	-<br>-	-<br>-	-<br>-	Jhunjhunwala et al., (2014) Genome Biology, 15, 436.
74	*TMEM170A*	•				1.01	HIGH<br>LOW	6<br>4	24<br>17	14<br>13	-<br>3	42<br>48	86<br>85	9<br>6	91<br>482.5	Cleary et al., (2013) Hepatology, 58, 1693-702.
75	*TRPC6*	•				1.04	HIGH<br>LOW	6<br>4	23<br>18	16<br>11	-<br>3	41<br>49	86<br>85	7<br>8	68.5<br>505.5	Fujimoto et al., (2016) Nature Genetics, 48, 500-9.
76	*TTLL2*	•				1.11	HIGH<br>LOW	4<br>6	20<br>21	14<br>13	-<br>3	48<br>42	86<br>85	8<br>7	91<br>410	Cleary et al., (2013) Hepatology, 58, 1693-702.
77	*UBR3*	•				0.99	HIGH<br>LOW	5<br>5	21<br>20	13<br>14	-<br>3	47<br>43	86<br>85	4<br>11	1029.5<br>131	Fujimoto et al., (2012) Nature Genetics, 44, 760-4.
78	*USH2A*	•				1.15	HIGH<br>LOW	5<br>5	21<br>20	13<br>14	1<br>2	46<br>44	86<br>85	9<br>6	268<br>228	Shirashi et al., (2014) PLoS ONE, 9, e114263.
79	*USP25*	•				1.05	HIGH<br>LOW	5<br>5	22<br>19	13<br>14	1<br>2	45<br>45	86<br>85	7<br>8	91<br>365	Fujimoto et al., (2012) Nature Genetics, 44, 760-4.
80	*VCX*	•				0.97	HIGH<br>LOW	6<br>-	21<br>-	15<br>1	1<br>-	43<br>4	86<br>5	8<br>-	555<br>-	Ahn et al., (2014) Hepatology, 60, 1972-82.
81	*VPS45*	•				1.05	HIGH<br>LOW	4<br>6	23<br>18	11<br>16	1<br>2	47<br>43	86<br>85	7<br>8	198<br>290.5	Fujimoto et al., (2016) Nature Genetics, 48, 500-9.

82	*WWP1*	•				1.02	HIGH<br>LOW	3<br>7	17<br>24	16<br>11	1<br>2	49<br>41	86<br>85	4<br>11	61<br>387.5	Fujimoto et al., (2012) Nature Genetics, 44, 760-4.
83	*ZIC3*	•				0.40	HIGH<br>LOW	-<br>-	-<br>-	-<br>-	-<br>-	-<br>-	-<br>-	-<br>-	-<br>-	Fujimoto et al., (2012) Nature Genetics, 44, 760-4.
84	*ZNF208*	•				0.73	HIGH<br>LOW	8<br>2	20<br>21	12<br>15	-<br>3	46<br>44	86<br>85	8<br>7	460<br>131	Shirashi et al., (2014) PLoS ONE, 9, e114263.
85	*ZNF226*	•				0.94	HIGH<br>LOW	5<br>5	21<br>20	17<br>10	1<br>2	42<br>48	86<br>85	6<br>9	482.5<br>131	Fujimoto et al., (2012) Nature Genetics, 44, 760-4.

The table indicates the nature of the mutation (SNV, indels, structural variants or copy number alterations) in the coding regions. The fold-change of the gene is obtained from the TCGA microarray analysis on HCC patient samples. Histologic grade refers to degree of tumor grade: G1 to G4, and G_un indicates cases with unidentified histologic grading. The cases are segregated into HIGH or LOW based on their median gene expression (Median Exp). SNVs and indel mutations are indicated by the yellow box (●), structural variants by the blue box (●) and copy number alterations by the grey box (↓|↑).

Table 3. Summary of HBV Viral Integration Events Occuring in HCC Patients identified through High-Throughput Genomics Data.

		Host region				HBV integration in host sites							Histologic grade						Survival		
No.	Gene	Promoter	CDS	3'UTR	Intron	X	S	Precore/core	Polymerase	Viral sequence inserted	Gene expression fold-change in TCGA-HCC dataset (T/N)	Exp	G1	G2	G3	G4	G_un	Total Cases	Cases deceased	Median survival days	References
1	*CCNE1*	√	√		√	√	√	√		X protein, Precore/core protein, S	0.97	HIGH<br>LOW	5<br>5	23<br>18	13<br>14	2<br>1	43<br>47	86<br>85	7<br>8	482.5<br>131	Dong et al., (2015) PLoS ONE, 10, e0123175.<br>Fujimoto et al., (2016) Nature Genetics, 48, 500-9.<br>Jhunjhunwala et al., (2014) Genome Biology, 15, 436.<br>Sung et al., (2012) Nature Genetics, 44, 765-69.
2	*TERT*	√	√		√	√		√	√	Polymerase, X protein, Precore/core protein	0.97	HIGH<br>LOW	5<br>5	24<br>17	16<br>11	1<br>2	40<br>50	86<br>85	7<br>8	365<br>171	Fujimoto et al., (2012) Nature Genetics, 44, 760-4.<br>Fujimoto et al., (2016) Nature Genetics, 48, 500-9.<br>Jhunjhunwala et al., (2014) Genome Biology, 15, 436.<br>Shirashi et al., (2014) PLoS ONE, 9, e114263.<br>Sung et al., (2012) Nature Genetics, 44, 765-69.<br>Toh et al., (2013) Carcinogenesis, 34, 787-98.
3	*CDK15*				√	√	√	√	√	S, Polymerase, X protein, Precore/core protein	1.38	HIGH<br>LOW	5<br>5	25<br>16	15<br>12	1<br>2	40<br>50	86<br>85	8<br>7	228<br>363	Shirashi et al., (2014) PLoS ONE, 9, e114263.
4	*ROCK1*	√			√	√	√			X protein, S	1.00	HIGH<br>LOW	4<br>6	23<br>18	16<br>11	0<br>3	43<br>47	86<br>85	6<br>9	91<br>410	Sung et al., (2012) Nature Genetics, 44, 765-69.
5	*FN1*				√	√		√	√	Precore/core protein, X protein, polymerase	1.00	HIGH<br>LOW	5<br>5	19<br>22	16<br>11	2<br>1	44<br>46	86<br>85	7<br>8	555<br>91	Sung et al., (2012) Nature Genetics, 44, 765-69.
6	*APOA2*		√			√	√		√	Polymerase, X protein, S	1.07	HIGH<br>LOW	3<br>7	17<br>24	15<br>12	3<br>0	48<br>42	86<br>85	8<br>7	91<br>1694	Dong et al., (2015) PLoS ONE, 10, e0123175.
7	*MLL4*		√		√	√			√	Polymerase, X protein	-	HIGH<br>LOW	0<br>0	0<br>0	0<br>0	0<br>0	0<br>0	0<br>0	0<br>0	-<br>-	Fujimoto et al., (2016) Nature Genetics, 48, 500-9.
8	*ANGPT1*				√	√		√		X protein, Precore/core protein	1.02	HIGH<br>LOW	7<br>3	27<br>14	16<br>11	0<br>3	36<br>54	86<br>85	7<br>8	91<br>365	Jhunjhunwala et al., (2014) Genome Biology, 15, 436.<br>Jiang et al., (2012) Genome Research, 22, 593-601.
9	*SENP5*				√	√		√		X protein, Precore/core protein	1.05	HIGH<br>LOW	4<br>6	20<br>21	19<br>8	1<br>2	42<br>48	86<br>85	8<br>7	228<br>290.5	Sung et al., (2012) Nature Genetics, 44, 765-69.
10	*PRC1*		√			√		√		Precore/core protein, X protein	1.14	HIGH<br>LOW	4<br>6	22<br>19	18<br>9	1<br>2	41<br>49	86<br>85	6<br>9	91<br>365	Dong et al., (2015) PLoS ONE, 10, e0123175.
11	*UPF2*		√			√		√		Precore/core protein, X protein	1.01	HIGH<br>LOW	5<br>5	18<br>23	18<br>9	1<br>2	44<br>46	86<br>85	5<br>10	61<br>482.5	Dong et al., (2015) PLoS ONE, 10, e0123175.
12	*EML4*				√	√			√	Polymerase, X protein	1.00	HIGH<br>LOW	5<br>5	16<br>25	20<br>7	1<br>2	44<br>46	86<br>85	5<br>10	61<br>482.5	Fujimoto et al., (2016) Nature Genetics, 48, 500-9.
13	*ADAM5P*				√	√				X protein	-	HIGH<br>LOW	0<br>0	0<br>0	0<br>0	0<br>0	0<br>0	0<br>0	0<br>0	-<br>-	Fujimoto et al., (2012) Nature Genetics, 44, 760-4.

14	*FAM178A*		√			√				X protein	-	HIGH<br>LOW	0<br>0	0<br>0	0<br>0	0<br>0	0<br>0	0<br>0	0<br>0	-<br>-	Dong et al., (2015) PLoS ONE, 10, e0123175.
15	*FAM18B2*				√	√				X protein	-	HIGH<br>LOW	0<br>0	0<br>0	0<br>0	0<br>0	0<br>0	0<br>0	0<br>0	-<br>-	Fujimoto et al., (2012) Nature Genetics, 44, 760-4.
16	*FRAS1*				√	√				X protein	0.92	HIGH<br>LOW	5<br>5	16<br>25	17<br>10	1<br>2	47<br>43	86<br>85	6<br>9	1052<br>131	Fujimoto et al., (2012) Nature Genetics, 44, 760-4.<br>Shirashi et al., (2014) PLoS ONE, 9, e114263.
17	*GRXCR1*				√	√				X protein	0.77	HIGH<br>LOW	0<br>0	0<br>0	0<br>0	0<br>0	0<br>0	0<br>0	0<br>0	-<br>-	Fujimoto et al., (2016) Nature Genetics, 48, 500-9.
18	*LASS4*				√	√				X protein	-	HIGH<br>LOW	0<br>0	0<br>0	0<br>0	0<br>0	0<br>0	0<br>0	0<br>0	-<br>-	Fujimoto et al., (2016) Nature Genetics, 48, 500-9.
19	*NKAIN3*				√	√				X protein	1.61	HIGH<br>LOW	0<br>0	0<br>0	0<br>0	0<br>0	0<br>0	0<br>0	0<br>0	-<br>-	Fujimoto et al., (2016) Nature Genetics, 48, 500-9.
20	*TEKT3*				√	√				X protein	1.39	HIGH<br>LOW	6<br>4	21<br>20	14<br>13	1<br>2	44<br>46	86<br>85	8<br>7	91<br>460	Fujimoto et al., (2016) Nature Genetics, 48, 500-9.
21	*MDS1*				√		√			S	-	HIGH<br>LOW	0<br>0	0<br>0	0<br>0	0<br>0	0<br>0	0<br>0	0<br>0	-<br>-	Fujimoto et al., (2016) Nature Genetics, 48, 500-9.
22	*MYH1*				√		√			S	1.42	HIGH<br>LOW	0<br>0	0<br>0	0<br>0	0<br>0	0<br>0	0<br>0	0<br>0	-<br>-	Dong et al., (2015) PLoS ONE, 10, e0123175.
23	*HEATR6*		√						√	Polymerase	1.04	HIGH<br>LOW	6<br>4	23<br>18	17<br>10	0<br>3	40<br>50	86<br>85	9<br>6	228<br>290.5	Fujimoto et al., (2016) Nature Genetics, 48, 500-9.
24	*MED13L*				√				√	Polymerase	1.03	HIGH<br>LOW	4<br>6	21<br>20	16<br>11	0<br>3	45<br>45	86<br>85	7<br>8	68.5<br>482.5	Dong et al., (2015) PLoS ONE, 10, e0123175.
25	*ZNF318*		√						√	Polymerase	1.09	HIGH<br>LOW	5<br>5	17<br>24	18<br>9	1<br>2	45<br>45	86<br>85	6<br>9	91<br>410	Fujimoto et al., (2016) Nature Genetics, 48, 500-9.

The table indicate the genes and where the integration events occur. The fold-change of the gene is obtained from the TCGA microarray analysis on HCC patient samples. Histologic grade refers to degree of tumor grade: G1 to G4, and G_un indicates cases with unidentified histologic grading. The cases are segregated into HIGH or LOW based on their median gene expression (Median_Exp).

Figure 1. Summary of NGS databases in liver cancer showing its current and potential research direction

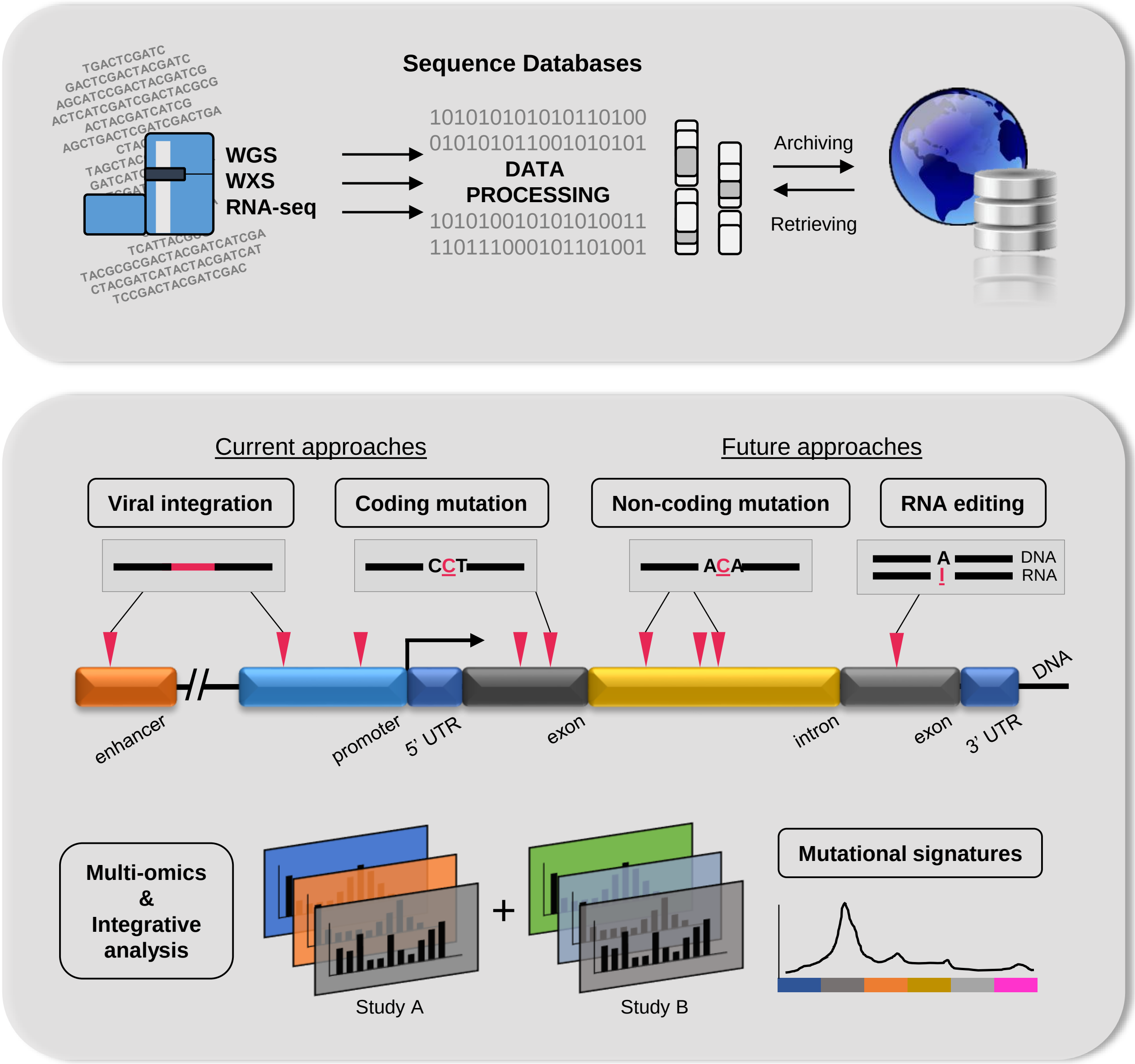

Figure 2. Reported Genes with Somatic Mutations that are significantly involved in KEGG Pathways

KEGG Orthology

Genes	Prostate cancer (hsa05215)	Endometrial cancer (hsa05213)	Glioma (hsa05214)	Melanoma (hsa05218)	Chronic myeloid leukemia (hsa05220)	Pathways in cancer (hsa05200)	Colorectal cancer (hsa05210)	Pancreatic cancer (hsa05212)	Viral carcinogenesis (hsa05203)	Bladder cancer (hsa05219)	Non-small cell lung cancer (hsa05223)	Hepatitis B (hsa05161)	HTLV-I infection (hsa05166)	PI3K-Akt signaling pathway (hsa04151)	Signaling pathways regulating pluripotency of stem cells (hsa04550)
CTNNB1	●	●	○	○	○	●	●	●	○	○	○	○	●	○	●
CDKN2A	○	○	●	●	●	●	○	●	●	●	●	○	●	○	○
CDKN1B	●	○	○	○	●	●	○	○	●	○	○	●	○	●	○
RB1	●	○	●	●	●	●	○	○	●	●	●	●	●	○	○
PIK3CA	●	●	●	●	●	●	●	●	●	○	●	●	●	●	●
PTEN	●	●	●	●	○	●	○	○	○	○	○	●	○	●	○
CCND1	●	●	●	●	●	●	●	●	●	●	●	●	●	●	○
CDKN1A	●	○	●	●	●	●	○	○	●	●	○	●	○	●	○
KRAS	●	●	●	●	●	●	●	●	●	●	●	●	●	●	●
APC	○	●	○	○	○	●	●	○	○	○	○	○	●	○	●
JAK1	○	○	○	○	○	●	○	●	●	○	○	●	●	●	●
CCNE1	●	○	○	○	○	●	○	○	●	○	○	●	●	●	○
TP53	●	●	●	●	●	●	●	●	●	●	●	●	●	●	○
AXIN1	○	●	○	○	○	●	●	○	○	○	○	○	○	○	●

Affected cases in each study

Genes	Ng et al., (2017)	Fujimoto et al., (2016)	Schulze et al., (2015)	Jhunjhunwala et al., (2014)	Ahn et al., (2014)	Kan et al., (2013)	Cleary et al., (2013)	Fujimoto et al., (2012)	Guichard et al., (2012)	Huang et al., (2012)	Li et al., (2011)
Total cases	98	300	236	12	231	88	87	27	24	110	139
CTNNB1	24	72	88	1	53	14	20	3	11		28
CDKN2A		17	7		14				2		
CDKN1B	3					1					
RB1	6	17	9		18	3					
PIK3CA											
PTEN	3	13	4		5						
CCND1		6	1		11						
CDKN1A			5		5						
KRAS					2						
APC			4		5				1		
JAK1			1			8					
CCNE1											
TP53	53	79	56	7	72	31	17	13	3	30	39
AXIN1	17	14	23	3	16	4			5		

Legend
- ○ Not involved in pathway
- ● Involved in pathway

Fold-change: 0 — 1 — >1

FDR values: 0 — <0.05 — 1

KEGG Orthology (KO)
- Cancers
- Signal Transduction
- Infectious Disease
- Cellular Processes

Personal Cover
Click here to access/download;Personal Cover;NGSReviewCoverLetter-v3.pdf

National Cancer Centre SINGAPORE

August 21, 2018

Laurie Goodman
Editor-in-Chief
Editorial Team
GigaScience
Oxford University Press

Submission of Review Article: 'Advances in Genomic Hepatocellular Carcinoma Research' by Weitai Huang, Anders Martin Jacobsen Skanderup, Caroline G. LEE

Dear Professor Goodman,

We would like to submit the following review manuscript entitled "Advances in Genomic Hepatocellular Carcinoma Research" by Huang, et al. for publication consideration as a Review Article in *GigaScience*.

We reviewed the availability of high-throughput data from the Next Generation Sequencing (NGS) of liver cancer patients within public repositories. Big data that is publicly available for hepatocellular carcinoma (HCC) has provided us with invaluable resources to better facilitate the identification of promising biomarkers or therapeutic targets. The current NGS resources and links to all publicly available NGS liver cancer datasets of approximately 489 whole genomes and 1100 whole exomes are consolidated in our review. Key somatic alterations and HBV integrations discussed in our review are most commonly reported in multiple high-impact publications. Many of these genes are also recurrent in liver cancer but not previously highlighted in other journal articles. This review presents a well consolidated information on publicly available data resources, as well as discusses critical genes that have been reported across journals and the future directions of HCC research using NGS datasets.

Notably, we have identified significant pathways associated with our consolidated list of gene candidates. While many of these genes are critical in cancer-related pathways, we present a good opportunity for HCC research towards establishing the remaining genes as critical drivers of HCC.

Although NGS studies in HCC have been previously reviewed, many advances have been made in the past two years in the field of HCC. The lack of a comprehensive and updated NGS resource is achieved in our review.

In summary, our review offers comprehensive insights into the important NGS resources and genes reported with somatic mutations and/or HBV integrations associated with HCC patients. Significantly, important insights about the pathways associated with our gene candidates consolidated from the literature was also gleaned from this study.

This manuscript has not been previously published, and has not been submitted for publication elsewhere while under consideration. In addition, we declare there is no conflict of interest that would prejudice the impartiality of this review.

We hope that this manuscript appropriate for publication as a Review Article in *GigaScience*.

Thank you.

11, Hospital Drive, Singapore 169610.
Phone: 65-6436-8353 • **Fax :** 65-6372-0161•
e-mail : dmslgl@nccs.com.sg or bchleec@nus.edu.sg

A member of SingHealth

Yours Sincerely,

Caroline Lee

Caroline G.L. Lee, PhD.
Associate Professor, Department of Biochemistry, National University of Singapore, Singapore
Principal Investigator, Division of Medical Sciences, National Cancer Center, Singapore
Associate Professor, Duke-NUS Graduate Medical School, Singapore
