# Supplementary material for: Advances in genomic hepatocellular carcinoma research
Source: Gigascience. 2018 Dec 6;7(12):giy135. doi: 10.1093/gigascience/giy135 (PMC6335342; doi:10.1093/gigascience/giy135)
Supplement: Response_to_Reviewer_Comments_Original_Submission.pdf [file giy135_response_to_reviewer_comments_original_submission.pdf]

Author's Response To Reviewer Comments

Close

Editor's comments:

Editor: Please add ORCID IDs for all authors in a list here. If you don’t have an ORCID ID you can register at https://orcid.org/
Author response: We have added the email addresses and ORCIDs accordingly

Editor: Please move all URLs to the reference list and only cite the Ref number here.
Author response: We have referenced the URLs in the reference list and modified the citations

Editor: Please add any abbreviations used more than once in the main text here in alphabetical order.
Author response: We have included the abbreviations used in the main text in the Abbreviations section under Declarations

Editor: (Author contributions) Please change the format here to a table as per our new Authorship Guidelines here:
https://academic.oup.com/gigascience/pages/authorship_guidelines
Author response: We have used the CRediT taxonomy for author contributions

Close
