# Supplementary material for: Advances in genomic hepatocellular carcinoma research
Source: Gigascience. 2018 Dec 6;7(12):giy135. doi: 10.1093/gigascience/giy135 (PMC6335342; doi:10.1093/gigascience/giy135)
Supplement: Response_to_Reviewer_Comments_Revision_1.pdf [file giy135_response_to_reviewer_comments_revision_1.pdf]

Author's Response To Reviewer Comments

Close

Point-by-point Response Letter

Reviewer 1, Summary
Reviewer comment
In this work, Huang et al. review and summarize most of the NGS studies related to HCC in the last few years.

The study is nicely conducted and well written, and will be very useful to easily access and integrate the high number of data sets generated by many teams around the world.
I have a few comments below.

Author response:
We would like to thank our reviewer for his appreciation of our work and for providing constructive suggestions to improve the manuscript.

Reviewer 1, Comment 1
Reviewer comment
The following references are missing and should be added :
- Zhang et al., Gastroenterology 2017, 49 WGS of aflatoxin B1 -related HCC: https://www.ncbi.nlm.nih.gov/pubmed/28363643
- Ng et al., Sci Transl Med 2017, WES of 98 HCC from Taiwan with a high contribution of aristolochic acid:
https://www.ncbi.nlm.nih.gov/pubmed/29046434
- Letouzé et al., Nat Commun 2017, WGS of 45 liver cancers from Europe with diverse etiological backgrounds:
https://www.ncbi.nlm.nih.gov/pubmed/29101368
- Chaudhary et al., Clin Cancer Res 2018, Meta-analysis of 1,494 HCC:
https://www.ncbi.nlm.nih.gov/pubmed/30242023

Author response:
We have cited and discussed about the above articles in the text of the manuscript as well as added the information to Table 1 and Table 2 accordingly.

Reviewer 1, Comment 2
Reviewer comment
The precise criteria used by the authors to include a gene in Table 2 should be specified, especially if the authors present this list as a reference of HCC driver genes. I would suggest to order genes according to a criteria (e.g. alteration frequency in HCC) to see the main drivers in the first lines.

Also, the reason why the authors discuss in detail ALB, ARID2, RB1, BRD7 and RPL22 rather than other genes is not clear to me.

Finally, although several genes harbor both mutations, SVs and CNAs, it may be worth to present separately genes that are primarily affected by SVs and CNAs (e.g. FGF19/CCND1, CCNE1, CDKN2A…). Otherwise they are a bit "drowned" in the sea of candidate drivers

from mutations.

Author response:
Noting that different types of mutations (SNVs, indels, SVs and CNAs) can be caused by different mechanisms, we are interested to identify HCC driver genes affected by more than one type of mutation.

In this order, it has brought to our attention that ALB, ARID2, RB1, BRD7 and RPL22 represent a set of HCC driver genes that are (1) affected by different mechanisms including SVs (5 out of 23) and CNAs (5 out of 10), (2) reported in multiple independent studies and (3) gives more appreciation to HCC driver genes found to harbor SVs and CNAs as compared to the well discussed HCC driver genes (TP53, CTNNB1 and AXIN1).

To help readers appreciate genes primarily affected by SVs and CNAs, we have added colour-tagged boxes to quickly differentiate them from SNVs and indels.

Reviewer 1, Comment 3
Reviewer comment
It has been shown that very highly expressed genes (like ALB or APOB) display a striking accumulation of indels, likely due to the collision of the transcription and replication machineries (Letouzé et al., Nat Commun 2017).

The authors should mention this alternate hypothesis to explain the recurrence of alterations in highly expressed liver genes.

Author response:
We thank this reviewer for highlighting this alternate hypothesis and we have included in the revised manuscript on (Page 7, lines 151-153).

Reviewer 1, Comment 4
Reviewer comment
HBV insertions in CCNA2 were also reported (Wang et al., Nature 1990 and Fujimoto et al., Nat Genet 2016) and should be mentioned.

Author response:
We have addressed these findings under the Future, “AAV2 viral integration events” section, (Page 13, lines 315-316).

Reviewer 1, Comment 5
Reviewer comment
Fujimoto et al. identified 7 signatures but 10 have been identified in a meta-analysis (Letouzé et al., Nat Commun 2017) and another signature related to cisplatin treatment was recently described (Boot et al., Genome Res 2018). I would suggest to mention these studies in the "mutational signature" paragraph, as well as the in-depth analyses of the aflatoxin B1 (Zhang et al., Gastroenterology 2017) and aristolochic acid (Ng et al., Sci Transl Med 2017) signatures.

Author response:
We have further elaborated in the “Mutational signature” paragraph with regards to these relevant findings important for our review (Page 11, lines 255-263).

Close
