# Supplementary material for: Advances in genomic hepatocellular carcinoma research
Source: Gigascience. 2018 Dec 6;7(12):giy135. doi: 10.1093/gigascience/giy135 (PMC6335342; doi:10.1093/gigascience/giy135)
Supplement: Reviewer_1_Report_(Original_Submission) -- Eric Letouze — 8/10/2018 Reviewed [file giy135_reviewer_1_report_(original_submission).pdf]

Reviewer Report

Title: **Advances in Genomic Hepatocellular Carcinoma Research**

Version: **Original Submission** **Date:** 10/8/2018

Reviewer name: **Eric Letouze**

Reviewer Comments to Author:

In this work, Huang et al. review and summarize most of the NGS studies related to HCC in the last few years.The study is nicely conducted and well written, and will be very useful to easily access and integrate the high number of data sets generated by many teams around the world.I have a few comments below.1. The following references are missing and should be added :- Zhang et al., Gastroenterology 2017, 49 WGS of aflatoxin B1-related HCC: https://www.ncbi.nlm.nih.gov/pubmed/28363643- Ng et al., Sci Transl Med 2017, WES of 98 HCC from Taiwan with a high contribution of aristolochic acid: https://www.ncbi.nlm.nih.gov/pubmed/29046434- Letouzé et al., Nat Commun 2017, WGS of 45 liver cancers from Europe with diverse etiological backgrounds: https://www.ncbi.nlm.nih.gov/pubmed/29101368- Chaudhary et al., Clin Cancer Res 2018, Meta-analysis of 1,494 HCC: https://www.ncbi.nlm.nih.gov/pubmed/302420232. The precise criteria used by the authors to include a gene in Table 2 should be specified, especially if the authors present this list as a reference of HCC driver genes. I would suggest to order genes according to a criteria (e.g. alteration frequency in HCC) to see the main drivers in the first lines.Also, the reason why the authors discuss in detail ALB, ARID2, RB1, BRD7 and RPL22 rather than other genes is not clear to me.Finally, although several genes harbor both mutations, SVs and CNAs, it may be worth to present separately genes that are primarily affected by SVs and CNAs (e.g. FGF19/CCND1, CCNE1, CDKN2A…). Otherwise they are a bit "drowned" in the sea of candidate drivers from mutations.3. It has been shown that very highly expressed genes (like ALB or APOB) display a striking accumulation of indels, likely due to the collision of the transcription and replication machineries (Letouzé et al., Nat Commun 2017). The authors should mention this alternate hypothesis to explain the recurrence of alterations in highly expressed liver genes.4. HBV insertions in CCNA2 were also reported (Wang et al., Nature 1990 and Fujimoto et al., Nat Genet 2016) and should be mentioned.5. Fujimoto et al. identified 7 signatures but 10 have been identified in a meta-analysis (Letouzé et al., Nat Commun 2017) and another signature related to cisplatin treatment was recently described (Boot et al., Genome Res 2018). I would suggest to mention these studies in the "mutational signature" paragraph, as well as the in-depth analyses of the aflatoxin B1 (Zhang et al., Gastroenterology 2017) and aristolochic acid (Ng et al., Sci Transl Med 2017) signatures.

Level of Interest

Please indicate how interesting you found the manuscript: Choose an item.

Quality of Written English

Please indicate the quality of language in the manuscript: Choose an item.

Declaration of Competing Interests

Please complete a declaration of competing interests, considering the following questions:

- Have you in the past five years received reimbursements, fees, funding, or salary from an organisation that may in any way gain or lose financially from the publication of this manuscript, either now or in the future?
- Do you hold any stocks or shares in an organisation that may in any way gain or lose financially from the publication of this manuscript, either now or in the future?
- Do you hold or are you currently applying for any patents relating to the content of the manuscript?
- Have you received reimbursements, fees, funding, or salary from an organization that holds or has applied for patents relating to the content of the manuscript?
- Do you have any other financial competing interests?
- Do you have any non-financial competing interests in relation to this paper?

If you can answer no to all of the above, write 'I declare that I have no competing interests' below. If your reply is yes to any, please give details below.

I declare that I have no competing interests

I agree to the open peer review policy of the journal. I understand that my name will be included on my report to the authors and, if the manuscript is accepted for publication, my named report including any attachments I upload will be posted on the website along with the authors' responses. I agree for my report to be made available under an Open Access Creative Commons CC-BY license (http://creativecommons.org/licenses/by/4.0/). I understand that any comments which I do not wish to be included in my named report can be included as confidential comments to the editors, which will not be published.

I agree to the open peer review policy of the journal

To further support our reviewers, we have joined with Publons, where you can gain additional credit to further highlight your hard work (see: https://publons.com/journal/530/gigascience). On publication of this paper, your review will be automatically added to Publons, you can then choose whether or not to claim your Publons credit. I understand this statement. Yes
